# Isolable fluorinated triphenylmethyl cation salts of [HCB_11_Cl_11_]^−^: demonstration of remarkable hydride affinity†

**DOI:** 10.1039/d1sc05936j

**Published:** 2022-04-04

**Authors:** S. Olivia Gunther, Chun-I Lee, Ellen Song, Nattamai Bhuvanesh, Oleg V. Ozerov

**Affiliations:** Department of Chemistry, Texas A&M University 3255 TAMU College Station Texas 77842 USA ozerov@chem.tamu.edu

## Abstract

Significantly fluorinated triarylmethyl cations have long attracted attention as potentially accessible highly reactive carbocations, but their isolation in a convenient form has proved elusive. We show that abstraction of chloride with a cationic silylium reagent leads to the facile formation of di-, tetra-, and hexafluorinated trityl cations, which could be isolated as analytically pure salts with the [HCB_11_Cl_11_]^−^ counterion and are compatible with (halo)arene solvents. The F_6_Tr^+^ cation carrying six *meta*-F substituents was computationally predicted to possess up to 20% higher hydride affinity than the parent triphenylmethyl cation Tr^+^. We report that indeed F_6_Tr^+^ displays reactivity unmatched by Tr^+^. F_6_Tr^+^ at ambient temperature abstracts hydrides from the C–H bonds in tetraethylsilane, mesitylene, methylcyclohexane, and catalyzes Friedel–Crafts alkylation of arenes with ethylene, while Tr^+^ does none of these.

## Abstract 2

Hexakis(*meta*-fluoro)substituted triphenylmethyl cation (F_6_Tr^+^) was isolated with a carborane partner anion. Its significantly higher hydride affinity compared to the parent (Tr^+^) allows it to abstract hydrides from a variety of C(sp^3^)–H bonds.

## Graphical abstract

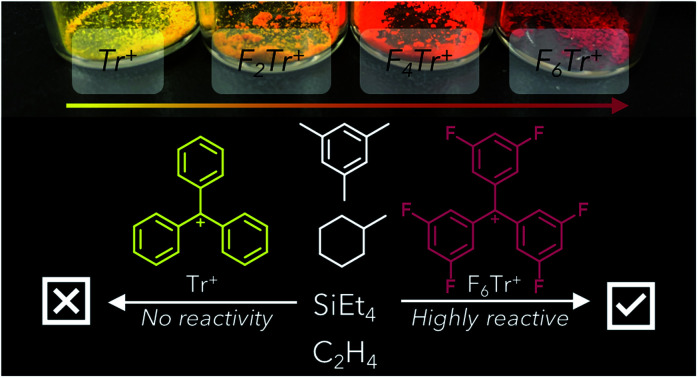

## Introduction

**Fig. 1:**
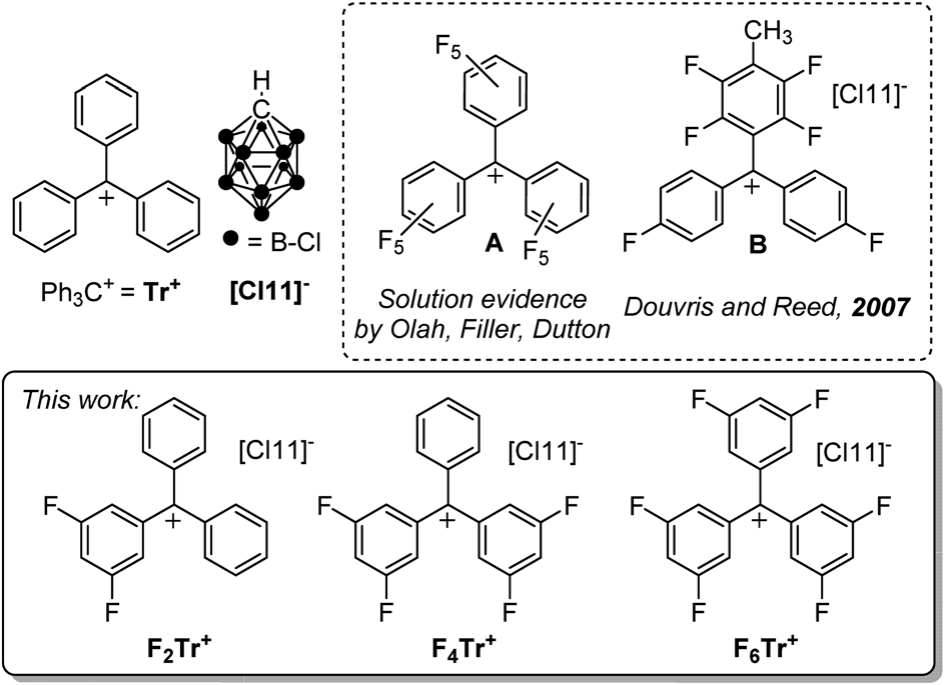
The parent trityl cation Tr^+^, selected literature examples of fluorinated trityls, and the fluorinated trityl salts prepared and studied in this work.

The triphenylmethyl or trityl cation (Ph_3_C^+^ or Tr^+^) is a textbook example of a carbocation that is isolable owing to the high degree of benzylic conjugation and the steric protection afforded to the central carbon by the three phenyl substituents.^1^ In organometallic chemistry and catalysis, salts of Tr^+^ are frequently used to study the thermodynamics and kinetics of hydride transfer,^2,3,4^ or to generate reactive main-group and transition-metal cations through hydride or alkyl anion abstraction.^5,6,7^Tr^+^ can also serve as a convenient one-electron oxidant.^8^ Trityl cation derivatives bearing stabilizing electron-donating groups can even exist in aqueous solutions, with a rich history of use as indicators and dyes.^9^ The trityl cation versions bearing electron-withdrawing substituents have proven more challenging to obtain. Fluorinated trityl cations, up to (C_6_F_5_)_3_C^+^ (A, Fig. 1), have been of particular fundamental interest,^10–12^ including as isoelectronic analogs of the widely used borane (C_6_F_5_)_3_B,^13,14^ and more recently have been studied by Horn and Mayr^15^ and Dutton *et al.*^16^ The more reactive A or other *ortho*- and/or *meta*-fluorinated trityl cations were not isolated in those studies, but were generated *in situ*, or their intermediacy was indicated by kinetic studies. While generation of fluorinated trityl cations in oleum and other superacidic media,^10–12,16^ or by *in situ* abstraction of halides with element halide Lewis acids^15^ is possible, these media and counteranions are not fully compatible with either the more electron-deficient trityl cations themselves or with their potential use in the synthesis of other reactive main-group or transition metal cations. Thus, the full extent of the reactivity of the fluorinated trityl cations can only be accessed when paired with more robust weakly coordinating anions in weakly coordinating solvents.^17^ The only example of an isolated trityl-type cation fluorinated in the *ortho*-/*meta*-positions is B (Fig. 1), obtained by Douvris and Reed in an undefined yield, and not studied further.^18^ The perchlorotrityl cation has also been isolated.^19^

Our group has been attracted to the highly reactive carbo- and main-group cations in the context of our work on silylium and alumenium cation-catalyzed activation of aliphatic C–F bonds,^20–23^ which permitted exhaustive defluorination of perfluoroalkyl groups under mild conditions. The chemistry of abstraction of fluoride from certain fluoroarenes with silylium cations has led to innovative reactivity, as well.^24,25^ Trialkylsilylium cations are typically generated by hydride abstraction from trialkylsilanes (R_3_SiH) using Tr^+^,^26^ but our theoretical analysis suggested that the parent Tr^+^ only barely has the thermodynamic hydride affinity (HA) to abstract hydrides from even the relatively electron-rich SiH bonds in trialkylsilanes. Given the perceived challenge^10,16^ in the isolation of the fully fluorinated (C_6_F_5_)_3_C^+^, we decided to first explore the partially fluorinated derivatives. Here, we report the isolation of analytically pure di-, tetra-, and hexafluorosubstituted trityl cation salts, and the remarkable contrast in the hydride abstraction reactivity with the parent Tr^+^.

## Results and discussion

### Theoretical HA analysis

Wilson and Dutton calculated gas-phase and CH_2_Cl_2_ solvent continuum HA values for a series of symmetric polychloro- and polyfluorosubstituted trityl cations.^27^ They discussed the fit to the known experimental values provided by the various computational methods and settled on the use of B3LYP/aug-cc-pVTZ//B3LYP/def2-TZVPP.^28,29^

The Wilson–Dutton calculations showed that replacement of H with F in the *para*-position has an essentially zero effect on HA, whereas introduction of each *ortho*- or a *meta*-fluorine increases HA by *ca.* 2.4–2.7 kcal mol^−1^ (CH_2_Cl_2_ continuum) or *ca.* 3.5 kcal mol^−1^ (gas phase). This is in line with the more negative p*K*_R+_ values for the various *ortho*- and *meta*-fluorinated trityls compared to Tr^+^ or the *para*-F substituted trityls, determined by Filler *et al.*^10^ The *ortho*- and *para*-CF positions are conjugated to the central carbon by resonance and the *para*-CF has been identified as a site of alternative nucleophilic attack on (C_6_F_5_)_3_C^+^ related to its decomposition pathways.^10,16^ We decided to avoid fluorination in the *ortho*- or *para*-positions and focus on *meta*-fluorination. The Wilson–Dutton HA values for F_6_Tr^+^ (213.0 and 108.3 kcal mol^−1^) were 11% and 17% higher than for Tr^+^ (191.4 and 92.5 kcal mol^−1^) in the gas phase and CH_2_Cl_2_ continuum, respectively.

In 2011,^30^ we analyzed the HA and FA values for a series of cations relevant to the silylium-catalyzed HDF using the M05-2X functional with the basis sets 6-311+G(d) for F, and 6-31++G(d,p) for C and H.^31^ Utilizing the DFT approach from our 2011 paper, we calculated the gas-phase and the chlorobenzene solvent continuum HA values for F_6_Tr^+^ to be 229.4 and 135.0 kcal mol^−1^, representing a 13% and a 20% increase *vs.*Tr^+^. These relative increases are similar to those in the Wilson–Dutton work.^27^ The substantial increase suggests that the HA of F_6_Tr^+^ is thermodynamically sufficient to abstract a hydride from a range of Si–H containing molecules, and rivals the HA values calculated (also in PhCl) for Me_3_C^+^ (126.6 kcal mol^−1^), PhCH_2_^+^ (137.8 kcal mol^−1^), and Me_2_CH^+^ (138.9 kcal mol^−1^).^30^ Without assessing quantitative accuracy, we nonetheless surmised that F_6_Tr^+^ might be able to abstract hydrides from tertiary and possibly secondary and benzylic C(sp^3^)–H bonds.

### Synthesis and characterization of F_*x*_Tr^+^ salts

We envisioned the synthesis of fluorinated trityl cations partnered with the exceptionally robust and weakly coordinating [HCB_11_Cl_11_]^−^ anion ([Cl11], Fig. 1)^18,32–35^*via* abstraction of a chloride anion from the corresponding F_2_TrCl, F_4_TrCl, and F_6_TrCl.^36,37^ Na[Cl11] can abstract a chloride from TrCl^38^ and from F_2_TrCl in *o*-C_6_H_4_Cl_2_ at ambient temperature, giving a 97% isolated yield of F_2_Tr[Cl11] after workup. Attempts to use Na[Cl11] to generate F_4_Tr[Cl11] and F_6_Tr[Cl11] were unsuccessful and we moved to a more powerful^39–41^ chloride abstractor [(Me_3_Si)_2_OTf][Cl11] (Si[Cl11]).^38,42^

**Fig. 2:**
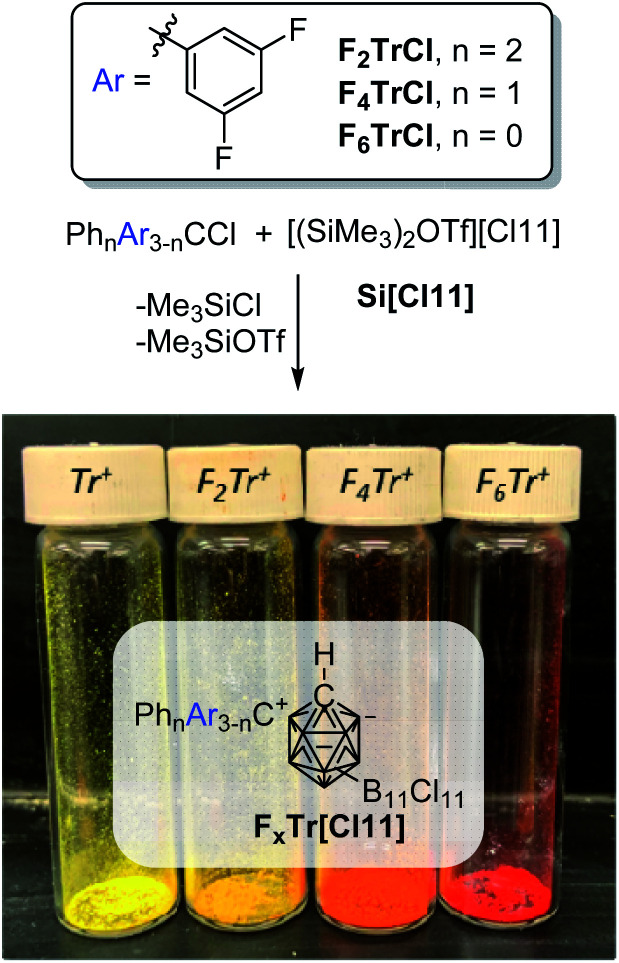
Synthesis of fluorinated trityl cation salts and their appearance.

Indeed, treatment of F_2_TrCl with Si[Cl11] in a 2 : 1 C_6_D_6_/*o*-C_6_H_4_Cl_2_ solvent mixture at ambient temperature (Fig. 2) resulted in rapid color change. Analysis of the resultant solution by NMR spectroscopy after 10 min revealed the expected formation of equimolar amounts of Me_3_SiCl and Me_3_SiOTf and 96% yield of F_2_Tr^+^ (^19^F NMR evidence, *δ* −104.6 ppm). The analogous reactions with F_4_TrCl and F_6_TrCl also proceeded smoothly. The resultant F_4_Tr[Cl11] and especially F_6_Tr[Cl11] are less soluble than F_2_Tr[Cl11] or Tr[Cl11], and precipitate readily out of fluorobenzene, allowing isolation of analytically pure solids in 96% and 70% yields.

**Fig. 3:**
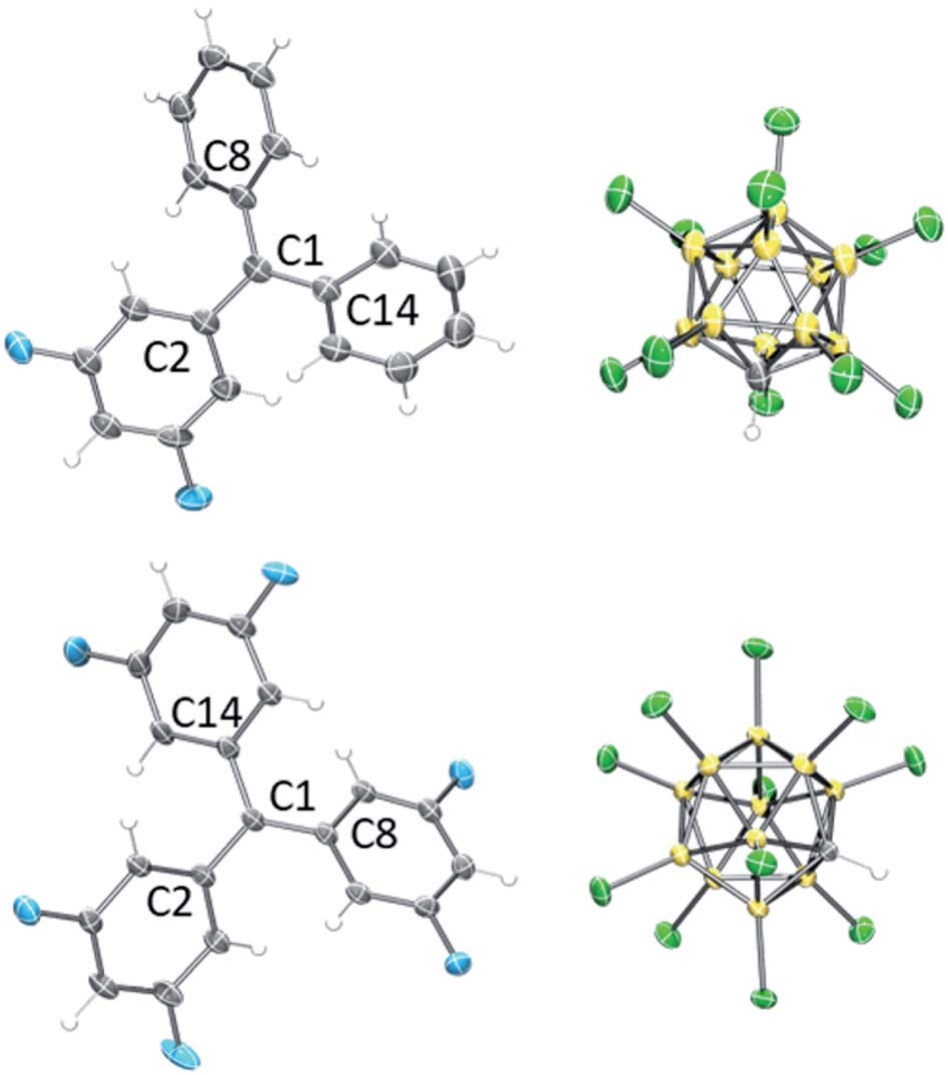
POV-Ray rendition of the ORTEP (50% probability ellipsoids) drawing of F_2_Tr[Cl11] (top) and F_6_Tr[Cl11] (bottom). Only one cation and one anion from each asymmetric unit is shown. Solvent and disorder are omitted for clarity.

The ^13^C NMR chemical shifts of the central carbons of F_2_Tr[Cl11], F_4_Tr[Cl11], and F_6_Tr[Cl11] in the 208–210 ppm range,^43^ as well as the ^1^H and ^19^F NMR spectral data did not suggest any significant interaction of the cations with the [Cl11]^−^ anion, the arene or CD_2_Cl_2_ solvents, or the Me_3_SiCl/Me_3_SiOTf by-products. Single-crystal X-ray diffractometry (Fig. 3) revealed that the central carbons in F_2_Tr[Cl11] and F_6_Tr[Cl11] possessed a planar environment in all the crystallographically independent cations (the sums of C–C–C angles are *ca.* 360°), and the aryl groups splay out in a pinwheel pattern about the central carbon. The closest approach of any chlorine to the central carbon in F_2_Tr[Cl11] is at least 3.7 Å, and 3.25 Å in F_6_Tr[Cl11], consistent with the well-separated, ionic nature of the F*_x_*Tr[Cl11] salts.

**Fig. 4:**
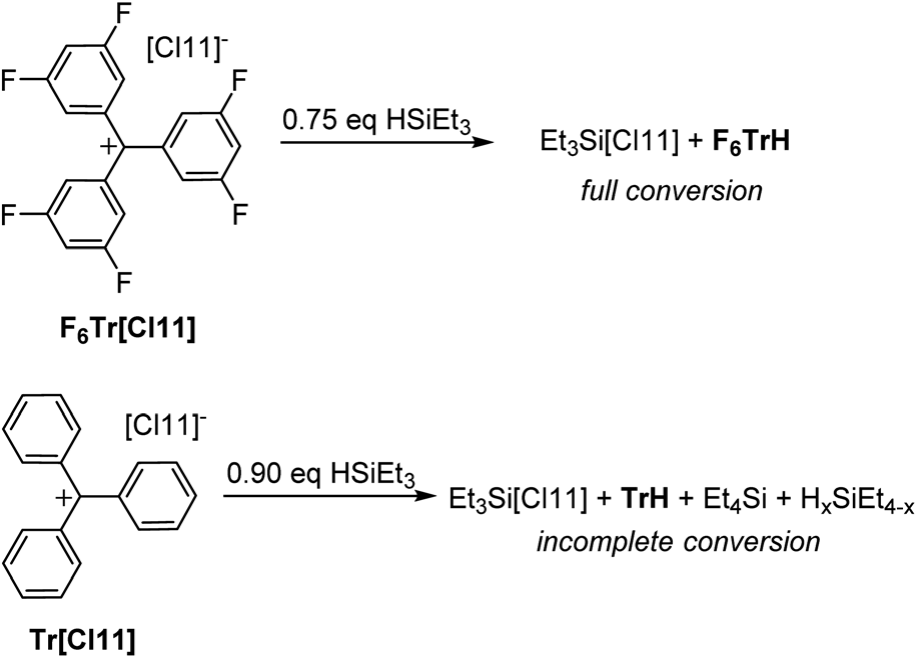
Reactions of Tr^+^ and F_6_Tr^+^ with a substoichiometric amount of HSiEt_3_.

### Reactivity of F_6_Tr^+^*vs.* Tr^+^ with Et_3_Si–H

It was previously shown that Tr^+^ is not capable of abstracting the full hydride equivalent from Et_3_SiH in non-coordinating solvents and that two equivalents of R_3_SiH are needed for complete formation of TrH.^44^ Our observations are similar: treatment of either F_6_Tr[Cl11] or Tr[Cl11] with two equivalents of Et_3_SiH in a C_6_D_6_/*o*-C_6_H_4_Cl_2_ solvent mixture led to the quantitative formation of F_6_TrH or TrH, respectively. The fate of the “Et_3_Si^+^” species in arene solvents is not straightforward, as has been studied in detail^45^ by Heinekey and coworkers: the presence of varying amounts of Et_4_Si betrays complexity arising from the H/Et redistribution in the Si species and/or reactions with the arenes.

The reaction of Tr[Cl11] with a substoichiometric (0.9 equiv.) amount of Et_3_SiH did not lead to the complete disappearance of the Si–H moiety (16% of the original Si–H intensity remained) and only 82% of the possible TrH was observed (Fig. 4). In contrast, the reaction of F_6_Tr[Cl11] with substoichiometric (0.75 equiv.) amount of Et_3_SiH led to the production of the expected quantity of F_6_TrH, the complete disappearance of the Si–H signals, and without the concomitant observation of Et_4_Si.

### H–D exchange

In the reactions of F_6_Tr[Cl11] with Et_3_SiH, significant H/D scrambling was observed among the neutral aromatic compounds present in solution: C_6_D_6_, *o*-C_6_H_4_Cl_2_, and F_6_TrH (but the C(sp^3^)–H bond in F_6_TrH was never deuterated). The extent of H–D exchange was analyzed *via*^1^H, ^13^C, or ^19^F^46^ NMR spectroscopy (see ESI† for details). The mechanism of the H/D exchange likely involves the generation of superacidic protonated arenes *in situ*,^47^ which should enable rapid H/D exchange *via* H^+^/D^+^ shuttling (Fig. S4†).^47,48^ The product of addition of either Et_3_Si^+^ or F_6_Tr^+^ to a neutral arene can be alternatively viewed as a protonated arene.^47^ It is also possible that analogous cations are accessed *via* reactions involving the minor components of the mixture. The Oestreich group recently examined this type of H/D exchange catalysis in greater detail.^49^

### Abstraction of hydride from C–H bonds

**Fig. 5:**
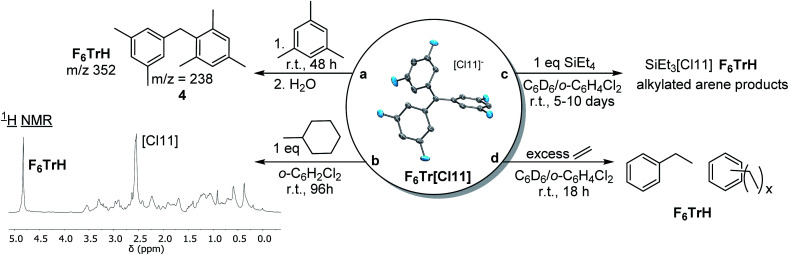
Reactions of F_6_Tr[Cl11] resulting in the abstraction of a hydride from C(sp^3^)–H bonds.

Given the computational prediction of the enhanced hydride affinity of F_6_Tr^+^*vs.*Tr^+^, we wished to examine their reactivity towards benzylic and aliphatic C–H bonds. As expected, no reaction was observed between Tr[Cl11] and (1) 1 equiv. of mesitylene or (2) 1 equiv. of methylcyclohexane in *o*-C_6_H_4_Cl_2_ after 1 week at ambient temperature. In contrast, the reaction of F_6_Tr[Cl11] with mesitylene (as solvent) resulted in 66% yield (NMR evidence) or F_6_TrH after 48 h. We propose that hydride abstraction from mesitylene by F_6_Tr[Cl11] generates a 3,5-dimethylbenzyl cation, which rapidly undergoes Friedel–Crafts^20,21^ addition to mesitylene. GC-MS analysis of the mixture after quenching with water showed the presence of a *m*/*z* signal at 238, consistent with compound 4 (Fig. 5). Treatment of F_6_Tr[Cl11] in *o*-C_6_H_4_Cl_2_ with 1 equiv. of methylcyclohexane resulted in the >95% yield (NMR evidence) of F_6_TrH after 96 h. The aliphatic region of the ^1^H NMR spectrum presented a large number of overlapping aliphatic signals, indicating a complex mixture (Fig. 5b).

The methylcyclohexyl cation presumed to be formed initially may undergo isomerization^50^ and Friedel–Crafts addition to *o*-C_6_H_4_Cl_2_, with many potential products. Abstraction of a hydride from alkanes, with generation of rearranged tertiary carbocations, was previously reported by the Reed group using Me[HCB_11_Me_5_Br_6_].^51,52^ The key difference between Reed's “Me^+^” reagents and the F_6_Tr^+^ reported here is that the latter can be prepared in bulk analytical purity and is stable in haloarene solutions.

Abstraction of a hydride from the β-position in trialkylaluminums with Tr^+^ has been used to generate reactive alumenium (R_2_Al^+^) cations.^6,22,53^ The analogous abstraction of β-hydride from alkylsilanes by Tr^+^ is not known, and we have confirmed that no reaction takes place between Tr[Cl11] and Et_4_Si in C_6_D_6_/*o*-C_6_H_4_Cl_2_. However, an analogous reaction of Et_4_Si with F_6_Tr[Cl11] resulted in the formation of 82% F_6_TrH after 96 h (and complete disappearance of Et_4_Si after 10 d). The major Si product appeared to be “Et_3_Si”, but instead of the stoichiometric complement of free ethylene, we observed ethane and other aliphatic resonances. Ethane may result from the protonolysis of Et_4_Si by the highly Brønsted acidic cations generated in the reaction (extensive H/D exchange was concomitantly observed), a process reported on by Oestrich and co-workers.^54^ As a control experiment, we examined the reaction of F_6_Tr[Cl11] with 6.3 equiv. of ethylene in C_6_D_6_/*o*-C_6_H_4_Cl_2_. Within 18 h at ambient temperature, all ethylene had been consumed, with the concomitant generation of ethylbenzene (1.8 equiv.) and other alkylarenes, and quantitative production of F_6_TrH. It is reasonable to propose that F_6_Tr[Cl11] abstracts a hydride from the benzylic positions of ethylbenzene or other alkylarenes generated through Friedel–Crafts alkylation. In complete contrast, no reaction occurred between Tr[Cl11] and ethylene under analogous conditions.

## Conclusion

Introduction of six *meta*-F substituents in F_6_Tr^+^ brought about remarkable contrast with the reactivity of the parent triphenylmethyl (Tr^+^) cation, understood primarily through the greatly enhanced hydride affinity of especially the hexafluorinated F_6_Tr^+^. Interestingly, while F_6_Tr^+^ catalyzes the Friedel–Crafts alkylation of arenes with ethylene, and generates alkyl cations *via* hydride abstraction which then readily engage in Friedel–Crafts addition, F_6_Tr^+^ itself is stable in combination with (halo)arene solvents and dichloromethane. This shows that fluorinated trityl cations represent a promising class of reagents for achieving the extremes of hydride affinity while minimizing reactivity with other potential substrates.

## Data availability

Data for this manuscript are available in the ESI.†

## Author contributions

S. O. G. and C. I. L. performed the syntheses and obtained the characterization data. E. S. performed the DFT calculations. N. B. carried out the X-ray diffraction studies on the crystals grown by S. O. G. S. O. G. and O. V. O. wrote the manuscript with assistance of the other co-authors. O. V. O. directed the overall effort.

## Conflicts of interest

There are no conflicts to declare.

## Supplementary Material

SC-013-D1SC05936J-s001

SC-013-D1SC05936J-s002

SC-013-D1SC05936J-s003
