# Supplementary material for: Isolable fluorinated triphenylmethyl cation salts of [HCB11Cl11]−: demonstration of remarkable hydride affinity
Source: Chem Sci. 2022 Apr 4;13(17):4972–6. doi: 10.1039/d1sc05936j (PMC9067617; doi:10.1039/d1sc05936j)
Supplement: SC-013-D1SC05936J-s001 [file SC-013-D1SC05936J-s001.pdf]

Electronic Supplementary Material (ESI) for Chemical Science.
This journal is © The Royal Society of Chemistry 2022

Electronic Supplementary Information

Isolable Fluorinated Triphenylmethyl Cation Salts of $[HCB_{11}Cl_{11}]^{-}$: Demonstration of Remarkable Hydride Affinity

S. Olivia Gunther, Chun-I Lee, Ellen Song, Nattamai Bhuvanesh, and Oleg V. Ozerov

Department of Chemistry, Texas A&M University, 3255 TAMU, College Station, TX 77842

ozerov@chem.tamu.edu

Table of Contents

I. General Considerations..3

II. X-Ray Structural Determination Details..5

III. Computational Details 10

IV. Synthesis of triarylmethanols and -methylchlorides 11

V. Synthesis of cations..15

VII. Spectroscopic data for relevant reagents in solvent mixtures, H/D exchange, and how to run a heterogenous reaction in a J. Young NMR tube..........................19

VIII. Reactivity Studies..23

IX. NMR, UV-Vis, and Mass Spectra. 30

X. References 108

I. General Considerations

Unless specified otherwise, manipulations for the synthesis of organic precursors were performed under an Ar atmosphere using standard Schlenk line and glovebox techniques. For the synthesis of salts or further reactivity studies, manipulations were performed in a glovebox under an Ar atmosphere free of donor solvents. Toluene, THF, diethyl ether, pentane, cyclohexane, and C_6D_6 were dried over NaK/Ph_2CO/18-crown-6, distilled or vacuum transferred and stored over molecular sieves in an Ar-filled glovebox. 3,5-difluorobromobenzene, CH_2Cl_2, $CDCl_3$, 1,2-orthodichlorobenzene (*o*-$C_6H_4Cl_2$), fluorobenzene (PhF), hexafluorobenzene (C_6F_6), 1,2-orthodifluorobenzene (*o*-$C_6H_4F_2$), methylcyclohexane, and all silanes were dried with and then distilled from CaH_2 and stored over molecular sieves in an Ar-filled glove box. Benzophenone was freshly sublimed before use.

The abbreviation **[Cl11]**$^-$ is used to refer to the undeca-chlorinated carborane monoanion, $[HCB_{11}Cl_{11}]^-$. Ph_3CCl (**TrCl**) and tris(3,5-difluorophenyl)methylchloride (**F$_6$TrCl**) were recrystallized from isooctane and toluene in a -35 °C glovebox freezer before use. Cs$[HCB_{11}H_{11}]$ was purchased from Katchem spol. s.r.o. (Czech Republic) and its chlorination (using the $SnCl_5$ method) and isolation of **Tr[Cl11]** and **Na[Cl11]** were carried out according to literature procedures.[1] **[(SiMe$_3$)$_2$OTf][Cl11]** has been previously reported[2] but was synthesized via a modified procedure for the analogous -Et compound, **[(SiEt$_3$)$_2$OTf][Cl11]**.[3] Syntheses and characterization data for (3,5-dichlorophenyl)diphenylmethanol (**F$_2$TrOH**),[4] bis(3,5-difluorophenyl)(phenyl)methanol (**F$_4$TrOH**),[5] bis(3,5-difluorophenyl)(phenyl)methylchloride (**F$_4$TrCl**)**Error! Bookmark not defined.** have been previously reported. **F$_6$TrCl** was prepared *via* a modified procedure reported for the corresponding bromide compound, **F$_6$TrBr**.**Error! Bookmark not defined.** All other chemicals were used as received from commercial vendors.

NMR spectra were recorded on a Varian Inova 400 (^{1}H NMR, 399.535 MHz), Bruker Avance Neo 400 (^{1}H NMR, 400.200 MHz; ^{13}C NMR, 100.630 MHz; ^{19}F NMR, 376.564 MHz; ^{11}B NMR, 128.400 MHZ, and ^{1}H NMR, 400.09 MHz; ^{13}C NMR, 100.603 MHz, respectively), NMRS 500 (^{1}H NMR, 499.703 MHz; ^{13}C NMR, 125.697 MHz; ^{19}F NMR, 469.854 MHz), and a Varian Inova 500 spectrometer (^{1}H NMR, 499.703 MHz, ^{13}C NMR 125.580 MHz, ^{19}F NMR, 469.854 MHz). Chemical shifts are reported in δ (ppm). For ^{1}H and ^{13}C NMR spectra, the residual solvent peak was used as an internal reference (^{1}H NMR: δ 7.15 for C_6D_6, 7.24 for $CDCl_3$, 5.32 for CD_2Cl_2); ^{13}C NMR: δ 128.06 for C_6D_6, 77.16 for $CDCl_3$, 29.84 for $(CD_3)_2CO$, 53.84 for CD_2Cl_2). For ^{19}F NMR, spectra were referenced externally to δ = -78.5 ppm by using CF_3COOH. Ultraviolent-visible (UV-vis) spectra were collected on a UV-2450 UV-Vis spectrophotometer (Shimadzu, Japan).

II. X-Ray Structural Determination Details

Data Collection and Structural Determination for **F_2Tr[Cl11]**×**0.75(*o*-$C_6H_4Cl_2$)**, CCDC 2079762.

A Leica MZ 75 microscope was used to identify a suitable colorless block with very well-defined faces with dimensions (max, intermediate, and min) 0.127 x 0.043 x 0.038 mm^3 from a representative sample of crystals of the same habit. The crystal mounted on a nylon loop was then placed in a cold nitrogen stream (Oxford) maintained at 110 K. A BRUKER Venture X-ray (kappa geometry) diffractometer was employed for crystal screening, unit cell determination, and data collection. The goniometer was controlled using the APEX3 software suite.[6] The sample was optically centered with the aid of a video camera such that no translations were observed as the crystal was rotated through all positions. The X-ray radiation employed was generated from a Cu-Iμs X-ray tube (K_α = 1.5418Å with a potential of 50 kV and a current of 1.0mA). 45 data frames were taken at widths of 1°. These reflections were used to determine the unit cell. The unit cell was verified by examination of the *h k l* overlays on several frames of data. No super-cell or erroneous reflections were observed. After careful examination of the unit cell, an extended data collection procedure (18 sets) was initiated using omega and phi scans.

Integrated intensity information for each reflection was obtained by reduction of the data frames with the program APEX3.[6] The integration method employed a three-dimensional profiling algorithm, and all data were corrected for Lorentz and polarization factors, as well as for crystal decay effects. Finally, the data was merged and scaled to produce a suitable data set. The absorption correction program SADABS[7] was employed to correct the data for absorption effects. Systematic reflection conditions and statistical tests of the data suggested the space group *P*-1. A solution was obtained readily (Z=4; Z'=2) using XT/XS in APEX3.[6,8] Hydrogen atoms were placed in idealized positions and were set riding on the respective parent atoms. All non-hydrogen atoms

were refined with anisotropic thermal parameters. The residual electron density peaks close to one of the $CHB_{11}Cl_{11}$ molecules suggested disorder and was modeled between two positions with an occupancy ratio of 0.87:0.13. Also, elongated thermal ellipsoids on one of the dichlorobenzene molecules suggested disorder and was modeled between two positions with an occupancy ratio of 0.87:0.13. Appropriate restraints and constraints were added to keep the bonds, angles, and thermal ellipsoids meaningful. Final Formula: $C_{19}H_{13}F_2 \cdot 0.75(C_6H_4Cl_2) \cdot HCB_{11}Cl_{11}$. Absence of additional symmetry or voids were confirmed using PLATON (ADDSYM).[9] The structure was refined (weighted least squares refinement on F^2) to convergence.[8,10]

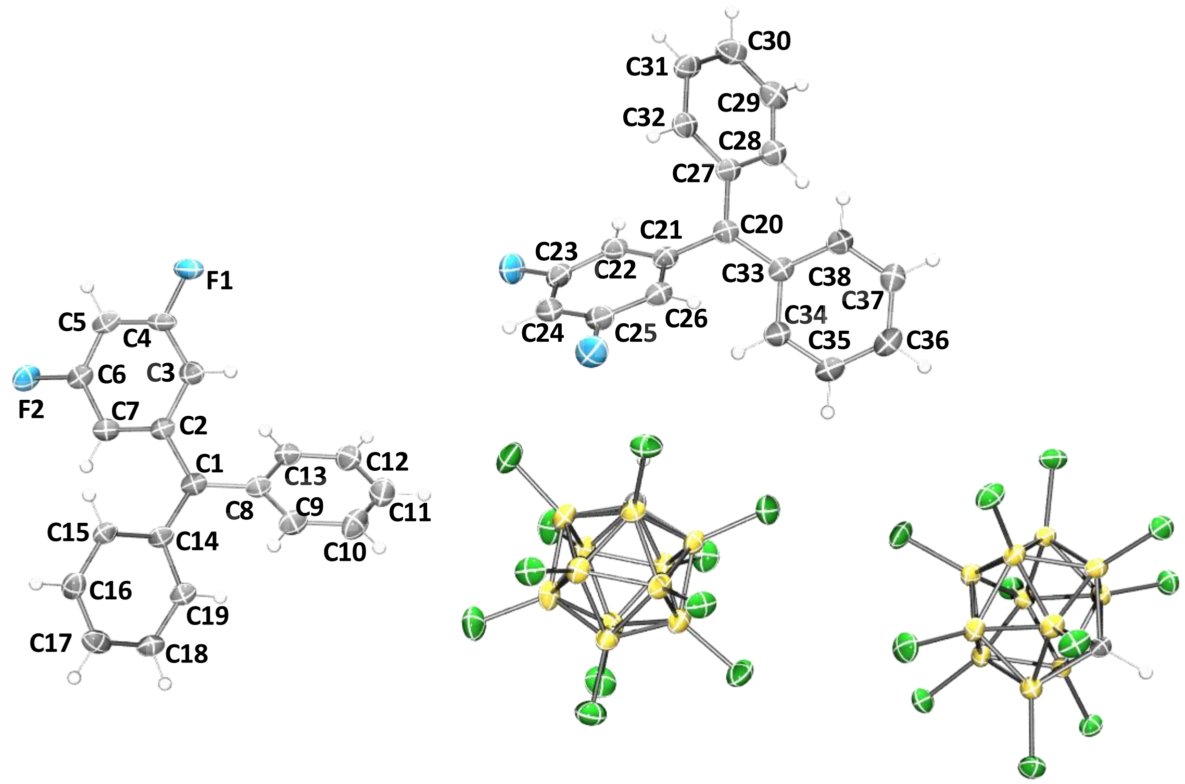

Figure S1. POV-Ray[11] rendition of the ORTEP[12] drawing (50% probability ellipsoids) of **F_2Tr[Cl11]**. The asymmetric unit contains two crystallographically independent cations, two anions, and two molecules of 1,2-dichlorobenzene. Disorder and solvent (1,2-dichlorobenzene) omitted for clarity.

Data Collection and Structural Determination for **F_6Tr[Cl11]**, CCDC 2079761

A Leica MZ 75 microscope was used to identify a suitable red block with very well-defined faces with dimensions (max, intermediate, and min) 0.217 x 0.182 x 0.124 mm^3 from a representative sample of crystals of the same habit. The crystal mounted on a nylon loop was then placed in a cold nitrogen stream (Oxford) maintained at 110 K. A BRUKER Quest X-ray (fixed-Chi geometry) diffractometer with a PHOTON III detector was employed for crystal screening, unit cell determination, and data collection. The goniometer was controlled using the APEX3 software suite.[6] The sample was optically centered with the aid of a video camera such that no translations were observed as the crystal was rotated through all positions. The X-ray radiation employed was generated from a Mo-Iμs X-ray tube (K_{α} = 0.71073Å). 45 data frames were taken at widths of 1°. These reflections were used to determine the unit cell. The unit cell was verified by examination of the *h k l* overlays on several frames of data. No super-cell or erroneous reflections were observed. After careful examination of the unit cell, an extended data collection procedure (17 sets) was initiated using omega and phi scans.

Integrated intensity information for each reflection was obtained by reduction of the data frames with the program APEX3.[6] The integration method employed a three-dimensional profiling algorithm, and all data were corrected for Lorentz and polarization factors, as well as for crystal decay effects. Finally, the data was merged and scaled to produce a suitable data set. The absorption correction program SADABS[7] was employed to correct the data for absorption effects. Systematic reflection conditions and statistical tests of the data suggested the space group C2. A solution was obtained readily (Z=6; Z'=1.5) using XT/XS in APEX3.[6,8] Hydrogen atoms were placed in idealized positions and were set riding on the respective parent atoms. All non-hydrogen

atoms were refined with anisotropic thermal parameters. One of the $CHB_{11}Cl_{11}$ molecules was located in a symmetry position. For this model, B6B and C6B was restrained at the same position, and with the same thermal ellipsoid; Cl6B occupancy was constrained at 0.5. Elongated thermal ellipsoids on Cl4B, and Cl5B suggested disorder and were modeled between two positions each with an occupancy ratio of 0.5:0.5. Final formula: $C_{20}H_{10}B_{11}Cl_{11}F_6$. Absence of additional symmetry and voids were confirmed using PLATON (ADDSYM).[9] The structure was refined (weighted least squares refinement on F^2) to convergence.[8,10]

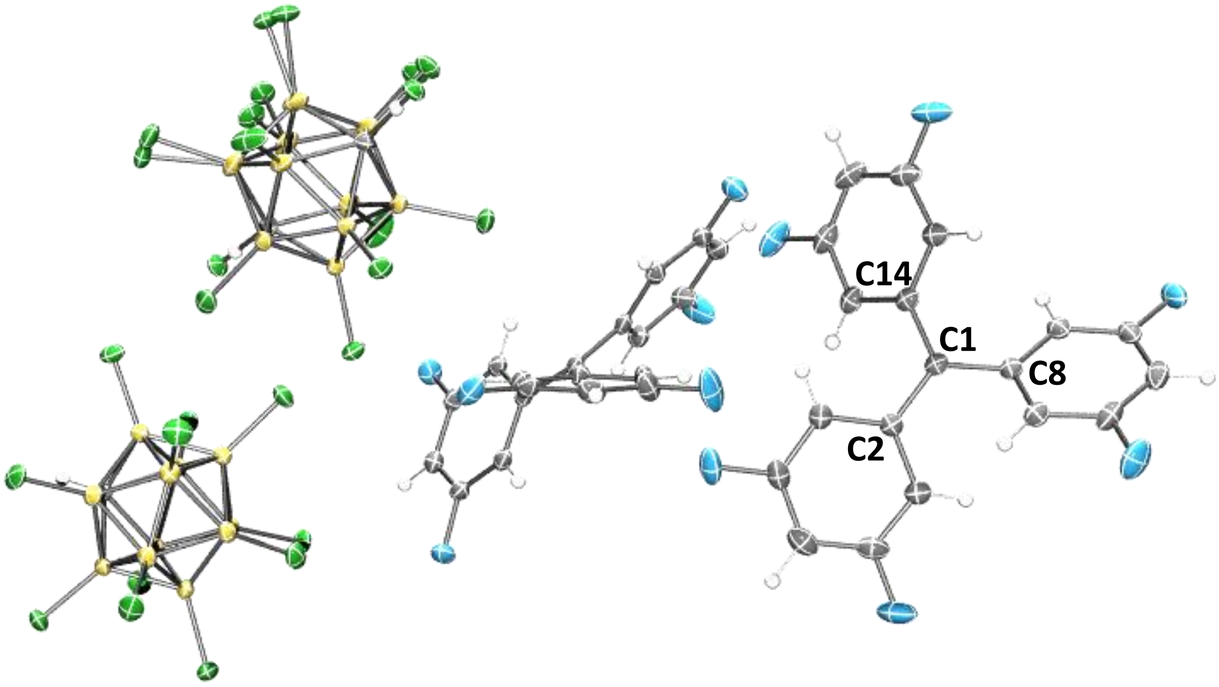

Figure S2. POV-Ray[10] rendition of the ORTEP[11] drawing (50% probability ellipsoids) of **F_6Tr[Cl11]**. The asymmetric unit contains 1.5 cations and 1.5 anions. Disorder in one of the anions removed for clarity.

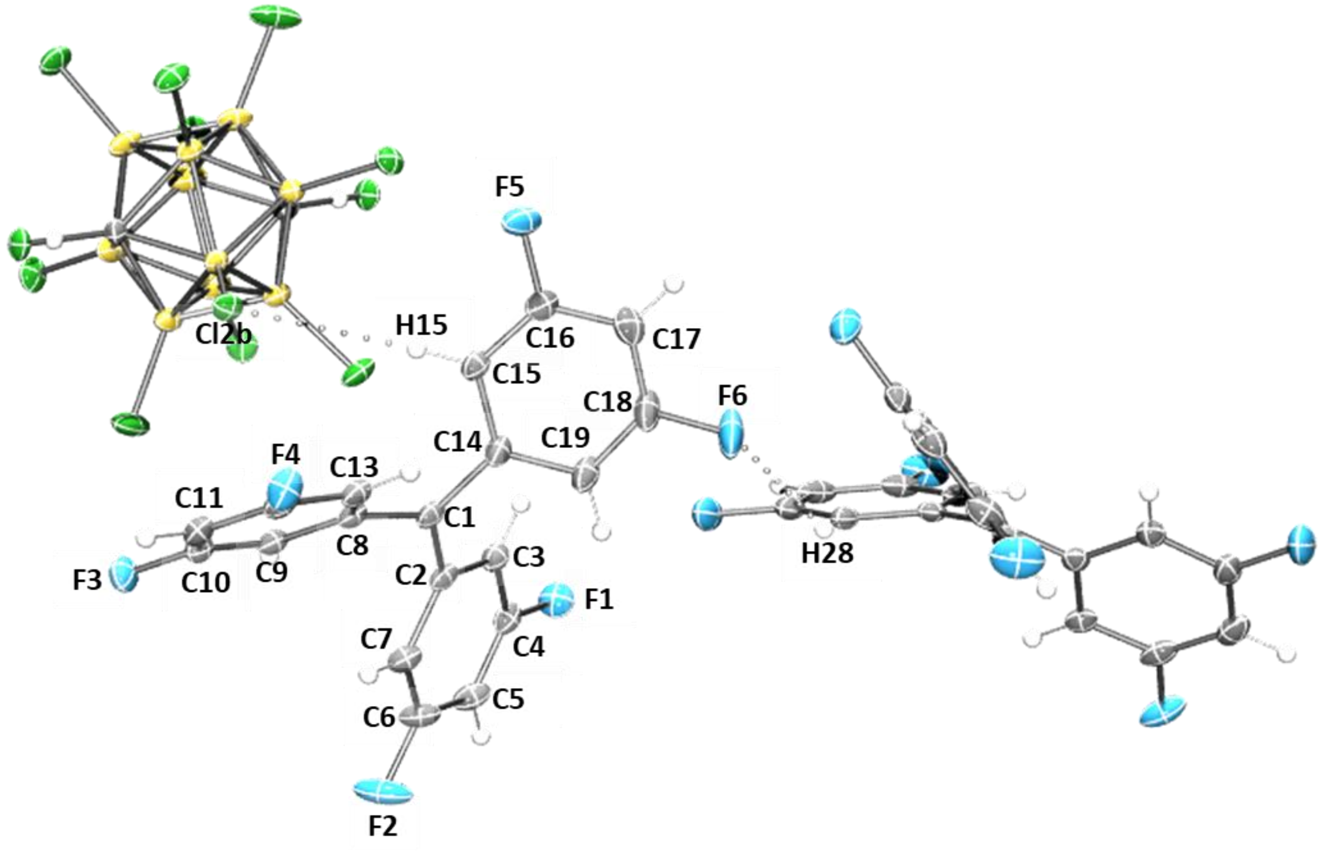

Figure S3. POV-Ray[10] rendition of the ORTEP[11] drawing (50% probability ellipsoids) of **F_6Tr[Cl11]**. The asymmetric unit contains 1.5 cations and 1.5 anions. Close contacts are observed between Cl2b-H15 (**Cl2b-C15:** 3.01 Å) and F6-H28 (**F6-C28:** 3.23 Å). One carborane anion removed for clarity.

III. Computational Details

Density Functional Theory (DFT) calculations to determine hydride and fluoride affinity in the gas phase and solution phase were performed using the Gaussian (09) program package. The M05-2X functional and ultrafine integration grid was used, with the basis sets 6-311+G(d) for F, and 6-31++G(d,p) for C and H. [13-14] These are the same methods used in our previously reported study.[15] As a starting point for the gas-phase optimizations, geometric parameters were adapted from the X-ray structure of **F_6Tr^+** in the solid state. Solution phase calculations were performed using the Polarized Continuum Model (PCM) with chlorobenzene as the solvent. In the gas phase, the hydride affinity of **F_6Tr^+** was calculated to be **229.4 kcal mol^{-1}** and the fluoride affinity was determined to be **183.1 kcal mol^{-1}** (**Scheme S1**). Solution phase calculations showed the hydride affinity of **F_6Tr^+** to be **135.0 kcal mol^{-1},** and the fluoride affinity to be **77.4 kcal mol^{-1}** (**Scheme S1**).

$$F_6Tr\text{-}H \longrightarrow F_6Tr^+ + H^- \qquad \Delta H = HA$$

$$F_6Tr\text{-}F \longrightarrow F_6Tr^+ + F^- \qquad \Delta H = FA$$

Scheme S1. Definitions of hydride affinity (HA) and fluoride affinity (FA).

IV. Synthesis of triarylmethanols and -methylchlorides

Synthesis of F_2TrOH. The title compound was synthesized according to a modified procedure reported by Johnson.**Error! Bookmark not defined.** In an Ar-filled glove box, magnesium powder (954 mg, 39.2 mmol), 1,2-dibromoethane (25 μL, 0.29 mmol) and THF (60 mL) were transferred to a 250 mL Schlenk flask. After stirring for 10 min, 3,5-difluorobromobenzene (3.00 mL, 26.1 mmol) was slowly added in 3 portions in 15 min intervals over the course of 45 min. During that time, the solution color turned from colorless to yellow. The solution temperature increased significantly, and boiling was observed. (**Note**: *Pause the addition of 3,5-difluorobromobenzene if the solution begins to boil too violently*.) After all 3,5-difluorobromobenzene had been added, the solution was left to stir at ambient temperature overnight. The solution was then filtered through Celite to remove excess magnesium. A solution of benzophenone (3.77 g, 20.7 mmol) in THF (10 mL) was added to the flask dropwise over 1 min. The solution was again left to stir at ambient temperature overnight. The flask was brought out of glovebox and quenched with 1 M HCl(aq) (80 mL). The solution color turned from dark brown to yellow. The organic layer was separated, and the water layer was extracted with hexanes (100 mL×2). The organic layer was combined and dried with anhydrous $MgSO_4$. The solution was filtered through Celite and dried in vacuo to yield a yellow oil. The oil was triturated by pentane and sonicated. White powder formed and precipitated, and the solution was filtered through a fritted funnel. The white powder was washed with cold pentane and dried in vacuo. Yield: 4.85 g (79%). The ^{1}H, ^{13}C, and ^{19}F NMR spectral data were in agreement with those reported in the literature.**Error! Bookmark not defined.**

Synthesis of F_2TrCl. The title compound was synthesized *via* a general procedure reported by Mayr.**Error! Bookmark not defined.** **F_2TrOH** (158 mg, 0.533 mmol) was dissolved in acetyl chloride (ca. 10 mL) in a 25 mL PTFE-valved gas-tight flask. The flask was heated to 65 °C overnight and then

allowed to cool to ambient temperature. After removing all volatiles *in vacuo*, the residues were redissolved in hexanes then filtered through Celite. The volatiles of the filtrate were removed *in vacuo* and the crude product was recrystallized from hexanes to yield a white solid (105 mg, 0.334 mmol, 63% first crop). ^{1}H NMR (400 MHz, C_6D_6): δ 7.20 (m, 4H), 6.95 (m, 4H), 6.91 (m, 2H) 6.35 (tt, J = 8.6, 2.3 Hz 3H). ^{13}C{^{1}H} NMR (101 MHz, C_6D_6): δ 162.8 (dd, $J_{F\text{-}C}$ = 249, 13 Hz), 150.0 (t, $J_{F\text{-}C}$ = 8.8 Hz), 144.6, 129.7, 128.4, 128.2, 113.4 (m), 103.6 (t, $J_{F\text{-}C}$ = 26 Hz), 80.2 (m, Ar_3C). ^{19}F NMR (376 MHz, C_6D_6) δ -109.7 (t, J = 8.5 Hz, 2F). HRMS (+APCI) m/z: $[M]^+$, calcd. For $C_{19}H_{13}F_2^+$ 279.0980; Found 279.0973.

Synthesis of F_4TrOH. The title compound was synthesized *via* a general procedure reported by Mayr.**Error! Bookmark not defined.** In an Ar-filled glove box, magnesium powder (1.82 g, 75.0 mmol), 1,2-dibromoethane (45 μL, 0.52 mmol) and THF (60 mL) were transferred to a 250 mL Schlenk flask. After stirring 10 min, 3,5-difluorobromobenzene (5.76 mL, 50.0 mmol) was slowly added in 3 portions in 15 min intervals over the course of 45 min. During that time, the solution color turned from colorless to yellow. The solution temperature increased significantly, and boiling was observed. (***Note****: Pause the addition of 3,5-difluorobromobenzene if the solution begins to boil too violently*.) After addition all of the 3,5-difluorobromobenzene, the solution was left to stir at ambient temperature overnight. The solution was then filtered through Celite to remove excess magnesium. Ethyl benzoate (3.00 mL, 21.0 mmol) was added to the flask dropwise over 1 min. The solution was left to stir at ambient temperature overnight. The flask was brought out of glovebox and quenched with 1 M HCl(aq) (80 mL). The solution color turned from dark brown to yellow. The organic layer was separated, and the water layer was extracted with hexanes (100 mL×2). The organic layer was combined and dried with anhydrous $MgSO_4$. The solution was filtered through Celite and dried in vacuo to yield a yellow oil. The oil was triturated by pentane

and sonicated. White powder formed and precipitated, and the solution was filtered through a fritted funnel. The white powder was washed with cold pentane and dried in vacuo. Yield: 3.24 g (46%). The ^{1}H and ^{13}C NMR spectral data were in agreement with those reported in the literature.**Error! Bookmark not defined.** ^{19}F NMR (470 MHz, C_6D_6) δ -110.2.

Synthesis of F_4TrCl. The title compound was synthesized *via* a general procedure previously reported by Mayr.**Error! Bookmark not defined.** **F_4TrOH** (196 mg, 0.612 mmol) was dissolved in acetyl chloride (ca. 10 mL) in a 25 mL s-valved gas-tight flask. The flask was heated to 65 °C overnight and then allowed to cool to ambient temperature. After removing all volatiles *in vacuo,* the residue was redissolved in hexanes and filtered through Celite. The volatiles of the filtrate were removed *in vacuo* and the crude product was recrystallized from hexanes to yield a white solid (56 mg, 0.160 mmol, 26%). The ^{1}H and ^{13}C NMR spectral data were in agreement with those reported in the literature.[**Error! Bookmark not defined.**] ^{1}H NMR (400 MHz, C_6D_6): δ 7.03 (m, 2H), 6.91 (m, 3H), 6.76 (m, 4H), 6.32 (tt, J = 8.5, 2.3 Hz, 2H). $^{13}C\{^{1}H\}$ NMR (101 MHz, (C_6D_6): δ 162.8 (dd, $J_{F\text{-}C}$ = 249.4, 12.7 Hz), 148.5 (t, $J_{F\text{-}C}$ = 8.6 Hz), 143.1 (s), 129.3, 128.8, 128.5, 113.1 (m), 104.0 (t, $J_{F\text{-}C}$ = 25.4 Hz), 78.6 (m, Ar_3C). ^{19}F NMR (470 MHz, C_6D_6) δ -108.3 (t, J = 8.5 Hz, 4F).

Synthesis of F_6TrCl. The title compound was synthesized in an analogous fashion to the bromide derivative, previously reported by Mayr.**Error! Bookmark not defined.** In an Ar-filled glovebox, 3,5-difluorobromobenzene (9.64 g, 50.0 mmol) was dissolved in 100 mL Et_2O in a 250 mL Schlenk flask. The flask was taken outside the glovebox, connected to a Schlenk line while maintain an Ar atmosphere, and placed in a -78 °C dry ice/acetone cooling bath. n-BuLi (20.0 mL of 2.5 M solution in hexanes, 50.0 mmol) was slowly added to the solution over the course of 10 min via syringe. The mixture was left to stir at -78 °C. After 3 h, diethyl carbonate (2.00 mL, 16.5 mmol) was added to the mixture over the course of 1 min via syringe and allowed to warm to -25 °C (a

cooling bath made with dry ice/ethanol : ethylene glycol = 3:7). After 3 h, 2 M HCl(aq) was added to quench the reaction. The organic layer was extracted with hexanes (100 mL×2) and dried over anhydrous $MgSO_4$. The hexanes solution was filtered through Celite and dried in vacuo to yield a yellow viscous oil. The oil was found to be **F_6TrOH** in 85% purity (^{19}F NMR), but no attempts were made to purify further. The crude oil was further dried in vacuo at 60 °C for 2 h. Acetyl chloride (25 mL, 350 mmol) was added to dissolve the oil and transferred to a 100 mL PTFE-cap gas-tight flask. The solution was placed in a 65 °C oil bath. After 1 d, the flask was allowed to cool to ambient temperature, and all volatiles were removed in vacuo to yield off-white solid. Pentane was added to the flask and sonicated. The solution was filtered through a fritted funnel, and the white powder was washed with cold pentane and dried in vacuo. Yield: 4.33 g (68%). **F_6TrCl**: ^{1}H NMR (400 MHz, C_6D_6): 6.57 (m, 6H), 6.24 (tt, J = 8.5, 2.3 Hz, 1H). ^{13}C{^{1}H} NMR (101 MHz, (C_6D_6): δ 162.8 (dd, $J_{F\text{-}C}$ = 250.1, 12.7 Hz), 147.0 (t, $J_{F\text{-}C}$ = 8.6 Hz), 112.8 (m), 104.5 (t, $J_{F\text{-}C}$ = 25.4 Hz), 77.0 (s). ^{19}F NMR (376 MHz, C_6D_6): -107.5 (t, J = 8.2 Hz, 6F). **F_6TrOH**: ^{1}H NMR (400 MHz, C_6D_6): δ 6.54 (m, 6H), 6.32 (tt, J = 8.6, 2.3 Hz, 3H), 1.76 (s, 1H, OH). ^{13}C{^{1}H} NMR (101 MHz, C_6D_6: δ 163.1 (dd, $J_{F\text{-}C}$ = 250.0, 12.6 Hz), 148.7 (t, $J_{F\text{-}C}$ = 8.1 Hz), 111.0 (m), 103.8 (t, $J_{F\text{-}C}$ = 25.4 Hz), 80.3. ^{19}F NMR (376 MHz, C_6D_6): δ -107.9 (t, J = 8.3 Hz, 6F).

V. Synthesis of cations

Reactions of Na[Cl11] with F_2-, F_4-, and F_6TrCl. Na[Cl11] was mixed with the appropriate F_xTrCl precursor in a J. Young NMR tube with *o*-$C_6H_4Cl_2$ (ca. 600 μL) in the presence of C_6F_6 (10 μL, internal standard). Conversion was determined based on the disappearance of **F_xTrCl** after the addition of **Na[Cl11]**. The results are tabulated in the following table. **F_2Tr[Cl11]** was successfully synthesized using this method and its isolation method is reported below. Using **F_4TrCl** or **F_6TrCl** resulted in incomplete conversion, and the low solubility of **F_4Tr[Cl11]** and especially **F_6Tr[Cl11]** led to their precipitation out of the reaction mixture, preventing separation from the NaCl by-product.

F_xTrCl	Amount (mmol)	Na[Cl11] added (mmol)	Conversion after 24 h	F_xTr^+ in solution after 24 h	Notes
F_2TrCl	0.048	0.048	94%	81%<br>0.039 mmol	Soluble in *o*-$C_6H_4Cl_2$
F_4TrCl	0.046	0.046	79%	11%<br>0.005 mmol	Somewhat soluble in *o*-$C_6H_4Cl_2$
F_6TrCl	0.057	0.057	81%	2%<br>0.001 mmol	Nearly insoluble in *o*-$C_6H_4Cl_2$<br>22% unidentified side products

Synthesis of [$(SiMe_3)_2$OTf][Cl11]. Tr[Cl11] (299 mg, 0.39 mmol) was dissolved in the minimum amount of PhF (ca. 6 mL) and transferred to a Teflon stopped Schlenk flask containing Me_3SiOTf (ca. 1 mL). The flask was brought out of the glovebox, degassed over the course of 3 freeze-pump-thaw cycles and refilled with $HSiMe_3$ (ca. 1 atm). Immediate loss of color and a warming of the solution occurred, suggesting consumption of Tr^+. The solution was stirred for 2 h at ambient temperature before concentrating in vacuo by 50%. The flask was transferred to a glovebox and the remaining solution was transferred to a 25 mL Schlenk flask. After the addition of pentane (ca.

5 mL), a white milky solid fell out of solution. Stirring the suspension resulted in a well-defined white powder and a clear, colorless supernatant. The supernatant was decanted, and the solid white product was washed thrice more with pentane and allowed to dry *in vacuo* for about 30 seconds, just until the solid looked dry. An off-white powder was isolated (250 mg, 0.30 mmol, 77%). Powders were stored in a -35 °C glovebox freezer and were used within 1 month. Over time, powders become more yellow. Best results are obtained when the reagent is used immediately after isolation. ^{1}H NMR (500 MHz, C_6D_6/*o*-$C_6H_4Cl_2$): δ 2.78 (br s, 1H, carborane), 0.21 (s, $J_{Si–H}$ = 6.9 Hz, 18H, $(SiMe_3)_2OTf$). ^{13}C{^{1}H} NMR (101 MHz, C_6D_6/*o*-$C_6H_4Cl_2$): δ 0.33. ^{19}F NMR (C_6D_6/*o*-$C_6H_4Cl_2$): δ -74.4 (s, 3F, -OTf).

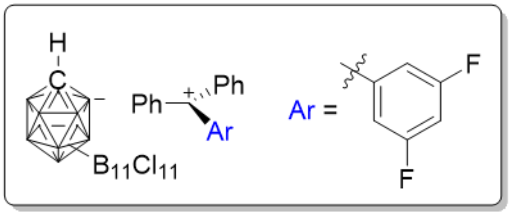

Synthesis of F_2Tr[Cl11] from Na[Cl11]. F_2TrCl (15 mg, 0.048 mmol) was dissolved in the minimum amount of *o*-$C_6H_4Cl_2$ (ca. 0.5 mL) in a J. Young NMR tube. **Na[Cl11]** (26 mg, 0.048 mmol) was added to the tube along with C_6F_6 (10 µL, 0.087 mmol) and the solution turned deep red. After 24 h, the contents of the NMR tube were filtered through a pipette of Celite. Pentane (ca. 2 mL) was added to the red solution to precipitate out a yellow solid. The solid was washed twice more with pentane (ca. 2 mL) and dried under reduced pressure to afford the title compound (37 mg, 97%). Crystals suitable for X-ray diffraction were grown from a solution of *o*-$C_6H_4Cl_2$ layered with pentane in a glovebox at ambient temperature for about one week. ^{1}H NMR (400 MHz, CD_2Cl_2): δ 8.41 (t, *J* = 7.3 Hz, 2H), 7.98 (t, *J* = 7.9 Hz, 4H), 7.76 (d, *J* = 7.60 Hz, 4H), 7.70 (t, *J* = 7.6 Hz, 1H), 7.25 (m, 2H), 3.17 (br, s). ^{13}C NMR (101 MHz, CD_2Cl_2): δ 209.1 (C^+), 163.4

(qC, F-ring), 146.3 (C-H), 139.8 (qC), 132.2 (C-H), 144.4 (C-H), 116.6 (C-H), 124.2 (C-H), 47.6 (carborane). ^{19}F NMR (376 MHz, CD_2Cl_2): δ -104.5 (m, 2F). ^{11}B NMR (128 MHz, CD_2Cl_2): δ -2.4 (1B), -10.1 (5B), -13.1 (5B). λ_{max} = 419, 462 nm. EA (%) calcd for $C_{20}H_{14}B_{11}Cl_{11}F_2$: C, 29.98; H, 1.76; N, 0.00; found: C, 30.30 H, 1.90; N, < 0.10.

***In situ* formation of F_2Tr[Cl11] with [(Me_3Si)$_2$OTf][Cl11]. F_2TrCl** (18 mg, 0.56 mmol) in a 2:1 C_6D_6/*o*-$C_6H_4Cl_2$ solution in a J. Young NMR tube. **[($SiMe_3$)$_2$OTf][Cl11]** (46 mg, 0.056 mmol, 1 eq), the solution immediately turned from colorless to a deep red, homogenous solution. A ^{1}H and ^{19}F NMR spectrum was taken after 10 min to reveal formation of Me_3SiOTf and Me_3SiCl (**Figure S25**) and **F_2Tr[Cl_{11}]** (**Figure S26**).

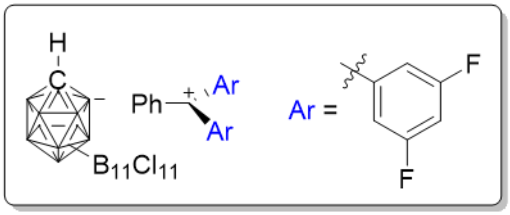

Synthesis of F_4Tr[Cl11]. [($SiMe_3$)$_2$OTf][Cl11] (40 mg, 0.12 mmol) was dissolved in the minimum amount of PhF (ca. 1 mL). **F_4TrCl** (94 mg, 0.12 mmol) was added and an immediate color change from colorless to bright red was observed and a bright orange/red precipitant formed. The reaction was allowed to stir at ambient temperature overnight. Pentane was added to the suspension to crash out an orange solid. After decanting the yellowish supernatant, the solid was washed with pentane (3 × 2 mL) and dried under reduced pressure to afford the title compound as a microcrystalline orange solid (93 mg, 96% yield. ^{13}C NMR chemical shifts were indirectly determined from 2D HSQC and HMBC experiments. ^{1}H NMR (400 MHz, CD_2Cl_2): δ 8.55 (tt, *J* = 7.5, 1.3 Hz, 1H), 8.05 (m, 2H), 7.86 (dd, *J* = 8.5, 1.3 Hz, 2H), 7.79 (tt, *J* = 7.9, 2.3 Hz, 2H), 7.29 (m, 4H), 3.17 (br s, 1H). ^{13}C NMR (101 MHz, CD_2Cl_2): δ 208.7 (C^+), 163.8 (CF), 150.3 (qC),

149.8 (CH), 146.1 (CH), 142.1 (qC), 132.7 (CH), 125.3 (CH), 118.5 (CH), 47.6 (carborane). ^{19}F NMR (376 MHz, CD_2Cl_2): δ -103.0 (m). ^{11}B NMR (128 MHz, CD_2Cl_2): δ -2.5 (1B), -10.1 (5B), -13.1 (5B). λ_{max} = 419, 488 nm. EA (%) calcd for $C_{20}H_{12}B_{11}Cl_{11}F_4$: C, 28.69; H, 1.44; N, 0.00; found: C, 28.38 H, 1.66; N, < 0.10.

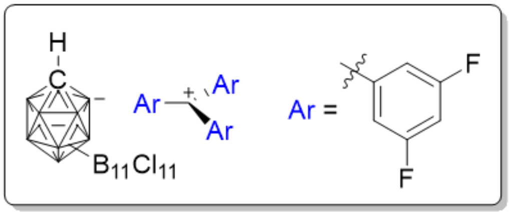

Synthesis of F_6Tr[Cl11]. [$(SiMe_3)_2$OTf][Cl11] (196 mg, 0.24 mmol) was dissolved in the minimum amount of PhF. **F_6TrCl** (102 mg, 0.26 mmol) was added and an immediate color change from colorless to deep mauve was observed. Over the course of two hours, a purple-red precipitate formed. After stirring for two hours at ambient temperature, pentane (ca. 5 mL) was added and more purple-red precipitant formed, leaving a yellowish supernatant. The microcrystalline solid was isolated on a frit and washed twice more with pentane before drying to afford the title compound (142 mg, 70% yield). ^{13}C chemical shifts were indirectly determined via 2D HSQC and HMBC experiments. ^{1}H NMR (400 MHz, CD_2Cl_2): δ 7.93 (tt, J = 7.7, 2.1 Hz, 3H, *p*-H), 7.40 (m, 6H, *o*-H), 3.20 (br, s H–C carborane). ^{13}C NMR (101 MHz, CD_2Cl_2): δ 210.0 (C^+), 164.0 (*m*-C–F), 126.7 (*o*-C), 125.9 (C-1), 121.1 (*p*-C), 47.7 (carborane). ^{19}F NMR (376 MHz, CD_2Cl_2): δ -101.1 (m, 6F). ^{11}B NMR (128 MHz, CD_2Cl_2): δ -2.6 (1B), -10.1 (5B), -13.1 (5B). λ_{max} = 421, 523 nm. EA (%) calcd for $C_{20}H_{10}B_{11}Cl_{11}F_6$: C, 27.51; H, 1.15; N, 0.00; found: C, 27.09 H, 1.14; N, < 0.10.

VII. Spectroscopic data for relevant reagents in solvent mixtures, H/D exchange, and how to run a heterogenous reaction in a J. Young NMR tube.

Spectroscopic data for relevant reagents in solvent mixture. Reactivity studies were carried out in C_6D_6/*o*-$C_6H_4Cl_2$ solvent mixtures. The relevant spectroscopic data can be found in the table below reference spectra. ^{1}H and ^{13}C NMR spectra were referenced to the residual benzene signal (^{1}H NMR: 7.16 ppm; ^{13}C NMR: 128.06 ppm). All resonances for F_6TrH can be observed in this solvent mixture. When H/D exchange is observed, the extend of deuterium incorporation on the aromatic rings in **F_6TrH** is determined by ^{1}H NMR spectroscopy, using the benzylic proton (**F_6Tr*H***) as an internal standard.

Reagent	^{1}H NMR (δ)	^{13}C{^{1}H} NMR (δ)	^{19}F NMR (δ)
C_6H_{12}	1.38 (s)	27.3 (s)	-
$HSiEt_3$	3.78 (br s, $J_{Si–H}$ = 177.6 Hz)<br>0.96 (t, *J* = 7.9 Hz)<br>0.54 (dq, *J* = 7.9, 2.9 Hz)<br>(**Figure S46**)	8.4, 2.9<br>(**Figure S47**)	-
$SiEt_4$	0.92 (t, *J* = 7.8 Hz)<br>0.47 (q, *J* = 7.8 Hz)<br>(**Figure S44**)	7.8, 3.4<br>(**Figure S45**)	-
F_6TrH	6.40 (t, *J* = 8.6 Hz, 3H), 6.33 (d, *J* = 6.3 Hz, 6H), 4.82 (s, 1H)<br>(**Figure S51**)	163.5 (dd, *J* = 250.0, 12.7 Hz), 145.3 (t, *J* = 8.5 Hz), 112.4 (m), 103.5 (t, *J* = 25.2 Hz), 55.4 (s).<br>(**Figure S52**)	-108.8 (t, *J* = 7.5 Hz)<br>(**Figure S53**)

Table S1. ^{1}H and ^{13}C NMR chemical shifts of compounds relevant to the following reactivity studies.

H/D Exchange Observations for F_6TrH – In reactivity experiments utilizing **F_6Tr[Cl11]** in the presence of a deuterated aromatic solvent, H/D exchange was observed between neutral aromatic species that contain C–H bonds. When using C_6D_6/*o*-$C_6H_4Cl_2$ solvent mixtures, new C–D coupling can be observed for *o*-$C_6H_4Cl_2$ by ^{13}C NMR spectroscopy. The intensity of the protio-benzene ^{1}H NMR resonances increased and the intensity of the protio-*o*-dichlorobenzene ^{1}H NMR resonances decreased.

All ^{1}H NMR resonances for **F_6TrH** are unobscured in C_6D_6/*o*-$C_6H_4Cl_2$ solvent mixtures and the extent of deuterium incorporation can be quantified by integrating the aromatic resonances against the benzylic C–H (**F_6Tr*H***) resonance. A combination of ^{1}H and ^{19}F NMR internal integration standards were used to rule out the generation of **F_6Tr*D***. By ^{19}F NMR spectroscopy, up to three signals were observed between -107.7 and -108.4 ppm for **F_6TrH**, each separated by ca. 0.2 ppm. We hypothesize that this observation was a result of the three unique isotopomeric environment about the arylfluorides: HFH, HFD, and DFD (**Figure S4**). This sort of isotope effect in deuterated fluoroarenes has been observed by Salamanca *et. al*. in their studies of D-labeled fluoroarenes.[16]

To further support the hypothesis, a known quantity of PhF was added to a reaction mixture containing Et_3Si^+ and **F_6Tr^+** (**R2**). A ^{19}F NMR spectrum was recorded to reveal three signals for PhF in an appropriate molar ratio with the **F_6TrH** signals (**Figure S4**). Remarkably, in less than 20 min, the majority of the HFH signals for PhF had disappeared. By ^{1}H NMR spectroscopy, **F_6TrH** was found to be 72% deuterated.

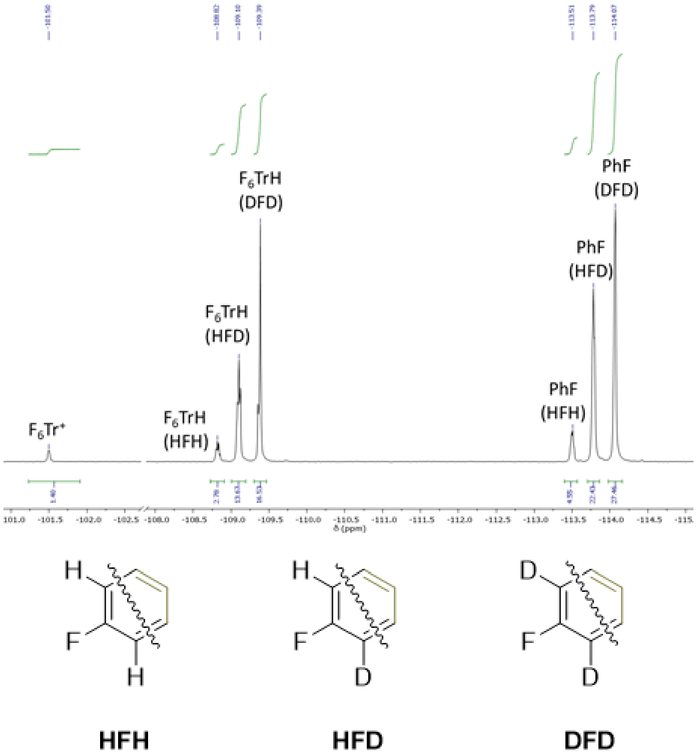

Figure S4. ^{19}F NMR spectrum and Chemdraw representations of the HFH, HFD, and DFD chemical environments.

We hypothesize that H/D exchange is being facilitated by a H^+/D^+ mechanism (**Figure S5**). It is not at present clear whether the silylium cations are more efficient at this than F_6Tr^+ itself. At ambient temperature, no H/D exchange was observed between C_6D_6 and *o*-$C_6H_4Cl_2$ at 0.4 mol% loading of **F_6Tr[Cl11]** alone (**R16**, below). However, upon heating the mixture at 45 °C for 24 h, 75% of *o*-$C_6H_4Cl_2$ was deuterated, according to ^1H NMR spectroscopy. It is possible that these observations are simply a consequence of the low solubility of **F_6Tr[Cl11]**. It should also be noted that we did not detect the formation of F_6Tr-Ar (**S2**, **Figure S5**) species that might result from the loss of proton from **S1**.

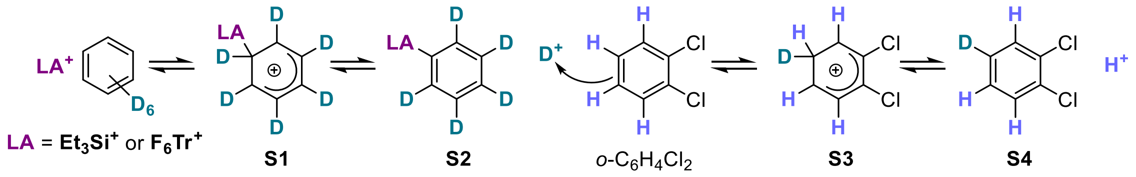

Figure S5. Proposed mechanism for the generation of o-$C_6H_3DCl_2$ in situ by Et_3Si^+ or F_6Tr^+. The counter anion, **[Cl11]**, is omitted for clarity.

Agitating heterogenous NMR scale reactions – Reactions with **F_6Tr[Cl11]** are heterogenous and need to be agitated. An apparatus consisting of a simple motor and various Kinex/Lego pieces mounted on a ring stand is used in these instances to constantly rotate the samples throughout the experiment (**Figure S6**).

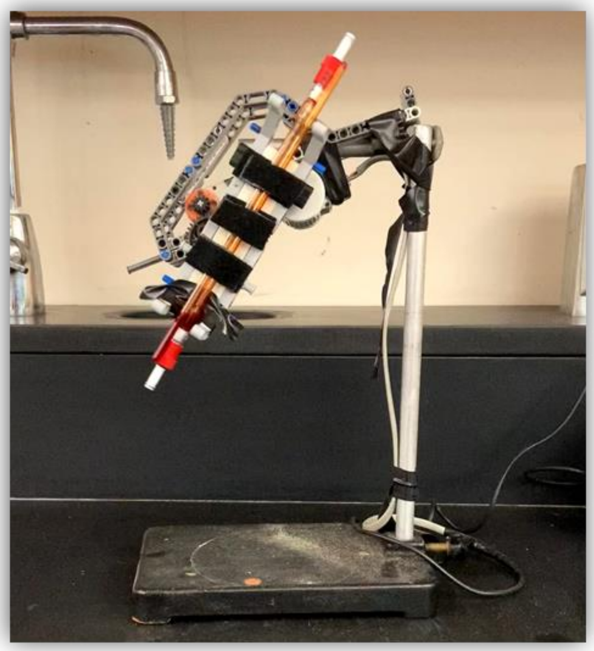

Figure S6. An apparatus used to rotate NMR tubes.

VIII. Reactivity Studies

R1. Synthesis of F_6TrH. **$F_6Tr[Cl11]$** (10 mg, 0.011 mmol, 0.06 eq) and **F_6TrCl** (70 mg, 0.206 mmol, 1 eq) were added to a 5 mL PTFE-valved gas-tight flask with a stir bar along with PhF (ca. 2 mL). The flask was degassed over the course of three freeze-pump-thaw cycles and was refilled with $HSiMe_3$ (1 atm, ca. 0.206 mmol, 1.3 eq). The solution turned from deep red to pale orange. After 2 hours, the reaction flask was degassed over 3 freeze-pump-thaw cycles and refilled again with more $HSiMe_3$ after which the solution turned yellow. The reaction was allowed to continue stirring at room temperature overnight. After 18 hours, the solution was colorless. The volatiles were removed in vacuo. Hexanes (ca. 2 mL) was added to the remaining white solid and the suspension was filtered through Celite. The hexanes were removed in vacuo to afford a low melting white solid (43 mg, 64% yield). ^{1}H NMR (500 MHz, C_6D_6): δ 6.33 (t, J = 8.7 Hz, 3H, p-H), 6.25 (br d, J = 6.2 Hz, 6H, o-H), 4.61 (s, 1H, Ar_3CH). ^{19}F NMR (470 Hz, C_6D_6): δ -109.1 (t, J = 7.7 Hz). $^{13}C\{^{1}H\}$ NMR (126 MHz, C_6D_6): δ 163.5 (dd, J = 249.7, 12.9 Hz), 145.2 (t, J = 8.6 Hz), 112.3 (m), 103.1 (dt, J = 25.3, 5.8 Hz), 55.3 (s). HRMS (-APCI) m/z: $[M-H]^-$, calcd. For $C_{19}H_9F_6^-$ 351.0614; Found 351.0613.

R2. Reaction of $F_6Tr[Cl11]$ with < 1 eq $HSiEt_3$. A 5 mL volumetric flask was used to make a stock solution of $HSiEt_3$ (299 mg, 0.51 M) and cyclohexane (105 mg, 0.25 M) in a 2:1 C_6D_6/*o*-$C_6H_4Cl_2$ solvent mixture. In a J. Young NMR tube, **$F_6Tr[Cl11]$** (17 mg, 0.020 mmol) was mixed with 30 μL of the stock solution, containing $HSiEt_3$ (0.015 mmol, 0.75 eq) and C_6H_{12} (internal standard, 0.008 mmol, 0.38 eq). Additional 2:1 C_6D_6/*o*-$C_6H_4Cl_2$ solvent mixture was added until the total volume was ca. 0.5 mL. By ^{1}H NMR spectroscopy, 0.015 mmol (75%, with respect to starting **F_6Tr^+**) of **F_6TrH** was generated and no Si-H resonances were observed. 0.015 mmol of Et_3Si^+ (quantitative, with respect to amount of $HSiEt_3$) was observed (**Figure S54**). By ^{19}F NMR

spectroscopy, three resonances in the range of **F_6TrH** were observed, a result of H/D exchange with C_6D_6 (**Figure S57**). ^{1}H NMR spectroscopy was used to determine deuterium incorporation and it was found that ca. 1% of the **F_6TrH** rings were deuterated. After 24 h, PhF (14.3 mg, 0.149 mmol) was added to the NMR tube as an internal ^{19}F NMR integration standard. A ^{19}F NMR spectrum was recorded 15 min after addition and three signals were observed for PhF as a result of H/D exchange (**Figure S58**). Using this integration standard, it was confirmed that 0.015 mmol (75%) of **F_6TrH** was formed by ^{19}F NMR spectroscopy. At this point, using ^{1}H NMR spectroscopy, 72% of the aromatic positions on the F_6TrH rings were deuterated (**Figure S59**). Red solids remained in the NMR tube, presumably unreacted **F_6Tr^+**.

R3. Reaction of Tr[Cl11] with < 1 eq $HSiEt_3$. A 5 mL volumetric flask was used to make a stock solution of $HSiEt_3$ (299 mg, 0.51 M) and cyclohexane (105 mg, 0.25 M) in a 2:1 C_6D_6/ *o*-$C_6H_4Cl_2$ solvent mixture. In a J. Young NMR tube, **Tr[Cl11]** (23 mg, 0.029 mmol) was mixed with 53 μL of the stock solution, containing $HSiEt_3$ (0.026 mmol, 0.9 eq) and C_6H_{12} (0.013 mmol, 0.45 eq). A ^{1}H NMR spectrum taken after one hour showed 0.021 mmol (74%) of TrH was formed, 0.0042 mmol (16%) of Si–H remained, and 0.0043 mmol (22%) of Et_4Si was observed (**Figure S60**). The major Si–Et containing product is likely $Et_3Si^+(HSiEt_3)_x$ in equilibrium which has been observed previously.[17] The volatiles were vacuum transferred to another J. Young NMR tube. Three neutral ethyl- containing compounds were observed, but their concentrations were too low to confidently identify.

R4. Reaction of F_6Tr[Cl11] with 2 eq $HSiEt_3$ – A 5 mL volumetric flask was used to make a stock solution of $HSiEt_3$ (299 mg, 0.51 M) and cyclohexane (105 mg, 0.25 M) in 2:1 C_6D_6/ *o*-$C_6H_4Cl_2$. In a J. Young NMR tube, **F_6Tr[Cl11]** (22 mg, 0.025 mmol) was mixed with 100 μL of the stock solution, containing $HSiEt_3$ (0.051 mmol, 2.04 eq) and C_6H_{12} (internal standard, 0.025

mmol, 1 eq) along with *o*-$C_6H_4F_2$ (26 mg, 0.23 mmol). A ^{1}H NMR spectrum was recorded after 10 min to reveal 0.025 mmol (>98%) of **F_6TrH**, 0.0072 mmol of Et_4Si (19%), and to 0.023 mmol (93%) of Si–H, presumably a hydride bridged silylium species similar to $Et_3Si^+(HSiEt)_x$ (**Figure S61**). A ^{19}F NMR spectrum also indicated that 0.025 mmol (>98%) of **F_6TrH** had formed (**Figure S62**). No H/D exchange was observed after 10 min by ^{19}F or ^{1}H NMR spectroscopy.

R5. Reaction of Tr[Cl_{11}] with 2 eq $HSiEt_3$. A 5 mL volumetric flask was used to make a stock solution of $HSiEt_3$ (299 mg, 0.51 M) and cyclohexane (105 mg, 0.25 M) in 2:1 C_6D_6/ *o*-$C_6H_4Cl_2$. In a J. Young NMR tube, **Tr[Cl11]** (33 mg, 0.043 mmol) was mixed with 170 μL of the stock solution containing $HSiEt_3$ (0.087 mmol, 2.02 eq), C_6H_{12} (internal standard, 0.043 mmol, 1.0 eq). By ^{1}H NMR spectroscopy, 0.037 mmol (86%) of Ph_3CH was generated alongside 0.014 mmol (22%) of Et_4Si and 82% of a major product, $Et_3Si^+(HSiEt)_x$ (**Figure S63**). The Si–H–Si ^{1}H NMR resonance overlaps with the carborane C–H ^{1}H NMR resonance.

R6. Reaction of F_6Tr[Cl11] with excess mesitylene. A stock solution of PhF (internal standard, 99.5 mg, 1.04 mmol, 0.21 M) in mesitylene was made in a 5 mL volumetric flask. A J. Young tube was charged with **F_6Tr[Cl11]** (15 mg, 0.01 mmol) and 500 μL of the above stock solution. The NMR tube was rotated at room temperature for the duration of the experiment (see **Figure S6**). The reaction was monitored by ^{19}F NMR spectroscopy for 2 days to track the appearance of **F_6TrH,** and the results are tabulated in **Table S2** (**Figure S64**). After 48 h, the reaction was quenched with a small amount of water and a GC/MS sample was run to reveal masses consistent with the product of Friedel-Crafts addition of $C_9H_{11}^+$ to another molecule of C_9H_{12} after removal of a proton by water, $C_{18}H_{22}$ (GC/MS: m/z = 238).

Time (h)	F_6TrH formed (%)
1	2
24	10
48	66

Table S2. % **F_6TrH** generation after 1, 24, and 48 h with respect to the internal standard, PhF.

R7. Reaction of Tr[Cl11] with excess mesitylene. In a 20 mL vial, **Tr[Cl11]** (13 mg, 0.017 mmol) was mixed with ca. 0.6 mL of mesitylene. The suspension was allowed to stir at ambient temperature. Over the course of two week, no change was observed by ^{1}H NMR spectroscopy (**Figure S68**).

R8. Reaction of F_6Tr[Cl11] and 1 eq methylcyclohexane. A 5 mL volumetric flask was used to make a stock solution of methylcyclohexane (25 mg, 0.052 M) in *o*-$C_6H_4Cl_2$. In a J Young NMR tube, **F_6Tr[Cl11]** (21 mg, 0.024 mmol) was mixed with 451 μL of the stock solution containing methylcyclohexane (0.024 mmol, 1 eq) and C_6F_6 (internal ^{19}F NMR standard) in *o*-$C_6H_4Cl_2$. More *o*-$C_6H_4Cl_2$ was added until the volume was ca. 0.5 mL. The NMR tube was rotated at room temperature for the duration of the experiment (see **Figure S6**). After 96 h, *o*-$C_6H_4F_2$ (20 mg, 0.024 mmol) was added to the NMR tube as an internal integration standard. By ^{19}F NMR spectroscopy, it was found that 0.024 mmol (>98%) of **F_6TrH** had formed (**Figure S70**). By ^{1}H NMR spectroscopy, many new aliphatic resonances were observed (**Figure S69**).

R9. Reaction of Tr[Cl_{11}] with 1 eq methylcyclohexane. A 5 mL volumetric flask was used to make a stock solution of methylcyclohexane (46 mg, 0.092 M) and C_6F_6 (86 mg, 0.092 M) in *o*-$C_6H_4Cl_2$. In a J Young NMR tube, **Tr[Cl11]** (18 mg, 0.023 mmol) was mixed with 250 μL of the stock solution containing methylcyclohexane (0.023 mmol, 1 eq) and C_6F_6 (0.023 mmol, 1 eq).

Additional *o*-$C_6H_4Cl_2$ was added until the total volume was ca. 0.5 mL. The reaction was monitored by ^{1}H NMR spectroscopy and no changes were observed after one week at room temperature (**Figure S71**).

R10. Reaction of F_6Tr[Cl11] with 1 eq Et_4Si. A 2 mL volumetric flask was used to make a stock solution of $SiEt_4$ (56 mg, 0.24 M), C_6F_6 (80 mg, 0.21 M), and cyclohexane (18 mg, 0.11 M) in a 2:1 C_6D_6/*o*-$C_6H_4Cl_2$ solvent mixture. In a J. Young NMR tube, **F_6Tr[Cl11]** (15 mg, 0.017 mmol) was added to 72 μL of the stock solution, containing $SiEt_4$ (0.017 mmol, 1 eq), C_6F_6 (0.015 mmol, 0.88 eq), and cyclohexane (0.008 mmol, 0.47 eq). Additional 2:1 C_6D_6/*o*-$C_6H_4Cl_2$ solvent mixture was added until the total volume was ca. 0.5 mL. The NMR tube was rotated at room temperature for the duration of the experiment (see **Figure S6**). A ^{1}H NMR spectrum was recorded after 5 days to reveal 0.015 mmol (85%) of **F_6TrH** alongside 0.012 mmol of Et_3Si^+ (71%). 0.002 mmol of Et_4Si remained (12%) at this point in addition to new aliphatic resonances (**Figure S72**). Analogous resonances were observed in **R10**. By ^{19}F NMR spectroscopy, 0.014 mmol (82%) of **F_6TrH** was observed as multiple resonances for different isotopomers (**Figure S73**). ^{1}H NMR spectroscopy was used to determine that 21% of the aromatic positions on **F_6TrH** were deuterated. Red solids remained in the tube, presumably unreacted **F_6Tr^+** starting material. After 10 days, no $SiEt_4$ remained, suggesting that the reaction had proceeded to completion, however a significant amount of ethane (1:0.44 **F_6TrH:C_2H_6**) and a large increase in the intensity of the C–H carborane resonance relative to the internal standard was observed (**Figure S74**). By ^{19}F NMR spectroscopy after 10 days, the concentration of the DFD resonance for **F_6TrH** increased (**Figure S75**). By ^{1}H NMR spectroscopy, it was determined that 71% of the aromatic positions on **F_6TrH** were deuterated at this point.

R11. Reaction of Tr[Cl11] with 1 eq Et_4Si. A 2 mL volumetric flask was used to make a stock solution of $SiEt_4$ (56 mg, 0.24 M), C_6F_6 (80 mg, 0.21 M), and cyclohexane (18 mg, 0.11 M) in 2:1 C_6D_6/*o*-$C_6H_4Cl_2$. In a J. Young NMR tube, **Tr[Cl11]** (20 mg, 0.026 mmol) was mixed with 109 μL of the stock solution, containing $SiEt_4$ (0.026 mmol, 1 eq), C_6F_6 (0.023 mmol, 0.88 eq), and cyclohexane (0.012 mmol, 0.46 eq). Additional 2:1 C_6D_6/*o*-$C_6H_4Cl_2$ solvent mixture was added until the total volume was ca. 0.5 mL. Over 5 days at ambient temperature, no change was observed by ^{1}H NMR spectroscopy (**Figure S76**).

R12. Reaction of F_6Tr[Cl11] and ethylene. **F_6Tr[Cl11]** (17 mg, 0.019 mmol) and C_6F_6 (10 μL, 0.087 mmol, 4.58 eq) were dissolved in a 2:1 C_6H_6/ *o*-$C_6H_4Cl_2$ mixture in a J. Young tube. The NMR tube was degassed over 3 freeze-pump-thaw cycles and refilled with ethylene (1 atm, ca. 0.12 mmol, ca. 6.3 eq). The NMR tube was rotated at room temperature for the duration of the experiment. After 18 hours, all ethylene had been consumed by ^{1}H NMR spectroscopy (**Figure S77**). 0.019 mmol (>98%) of **F_6TrH** was observed by ^{19}F NMR spectroscopy (**Figure S78**). The major product after 18 hours was ethylbenzene (0.035 mmol, ca. 1.8 eq) with ^{1}H NMR chemical shifts of 2.45 and 1.07 ppm and ^{13}C{^{1}H} NMR chemical shifts of 28.9 and 15.3 ppm (**Figure S79**).

R13. Reaction of Tr[Cl11] with ethylene. **Tr[Cl11]** (22 mg, 0.029 mmol) was dissolved in a 2:1 C_6D_6/ *o*-$C_6H_4Cl_2$ mixture in a J. Young tube, degassed over 3 freeze-pump-thaw cycles, and refilled with ethylene (1 atm, ca. 0.12 mmol, ca. 4.1 eq). No changes were observed by ^{1}H NMR spectroscopy after 1 week at room temperature. The procedure was repeated in 2:1 C_6H_6/*o*-$C_6H_4Cl_2$ and the results were identical (**Figure S80**).

R14. Reaction of F_6Tr[Cl11] with [Et_4N][Cl]. **F_6Tr[Cl11]** (11.6 mg, 0.013 mmol, 1 eq) and [Et_4N][Cl] (10 mg, 0.060 mmol, 4.6 eq) were added to a J. Young NMR tube with *o*-$C_6H_4Cl_2$ (ca.

600 μL). After shaking the heterogeneous mixture for 10 min, the contents of the tube turned from red to colorless. Analysis by ^{19}F NMR spectroscopy revealed two products, **F_6TrCl** (90%) and **F_6TrOH** (10 %), presumably from residual water contained in the ammonium salt (**Figure S81**).

R15. Reaction of F_6Tr[Cl11] and H_2O. **F_6Tr[Cl11]** (15 mg, 0.017 mmol) was added to an NMR tube with C_6D_6 (ca. 500 μL) and a drop of water. A quick shake turned the red suspension into a pale-yellow solution. By ^{19}F NMR spectroscopy, the only product observed was **F_6TrOH** (**Figure S82**).

R16. Evidence for H/D exchange catalyzed by F_6Tr[Cl11]. F_6Tr[Cl11] (10 mg, 0.011 mmol, 0.4%) was added to a solution of C_6D_6 (454 mg, 5.40 mmol, 2 eq), *o*-$C_6H_4Cl_2$ (396 mg, 2.69 mmol, 1 eq), and C_6F_6 (internal standard, 15 mg, 0.081 mmol) in a J. Young NMR tube. ^{1}H and $^{13}C\{^{1}H\}$ NMR spectra were recorded after 24 hours at ambient temperature that showed no change in H/D distribution (10.9 mmol H total). After 24 h at 45 °C, a ^{1}H NMR spectrum revealed that 8.07 mmol of H (75%) had been distributed to benzene and only 2.81 mmol of H (25%) remained in *o*-$C_6H_4Cl_2$ (ca. 0.703 mmol, 26%) (**Figure S83**). A $^{13}C\{^{1}H\}$ NMR spectrum taken at the same time showed evidence of deuterium incorporation into the *o*-$C_6H_4Cl_2$ (**Figure S84**). No species were observed by ^{19}F NMR spectroscopy

IX. NMR, UV-Vis, and Mass Spectra.

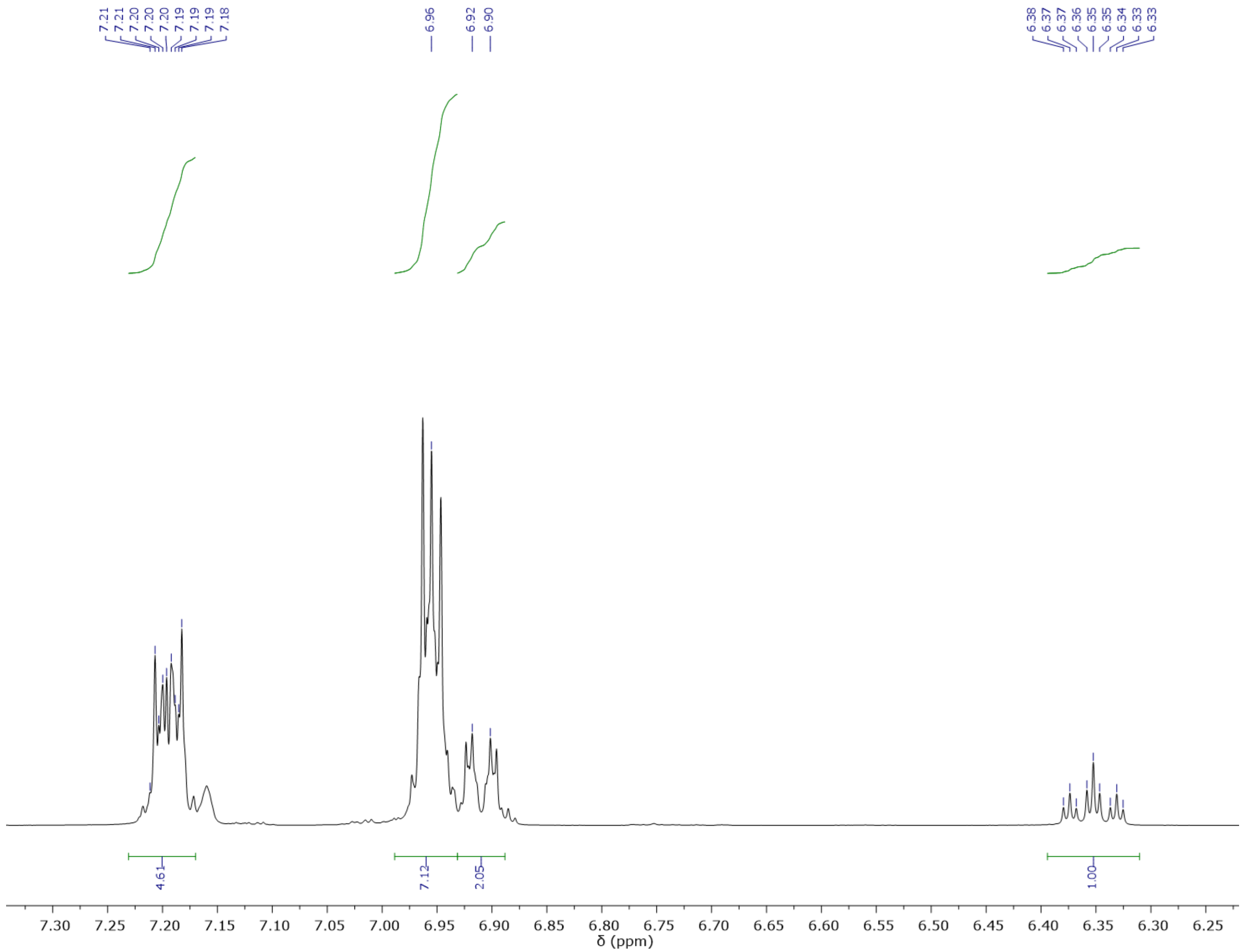

Figure S7. ^{1}H NMR (400 MHz, C_6D_6) spectrum of **F_2TrCl**.

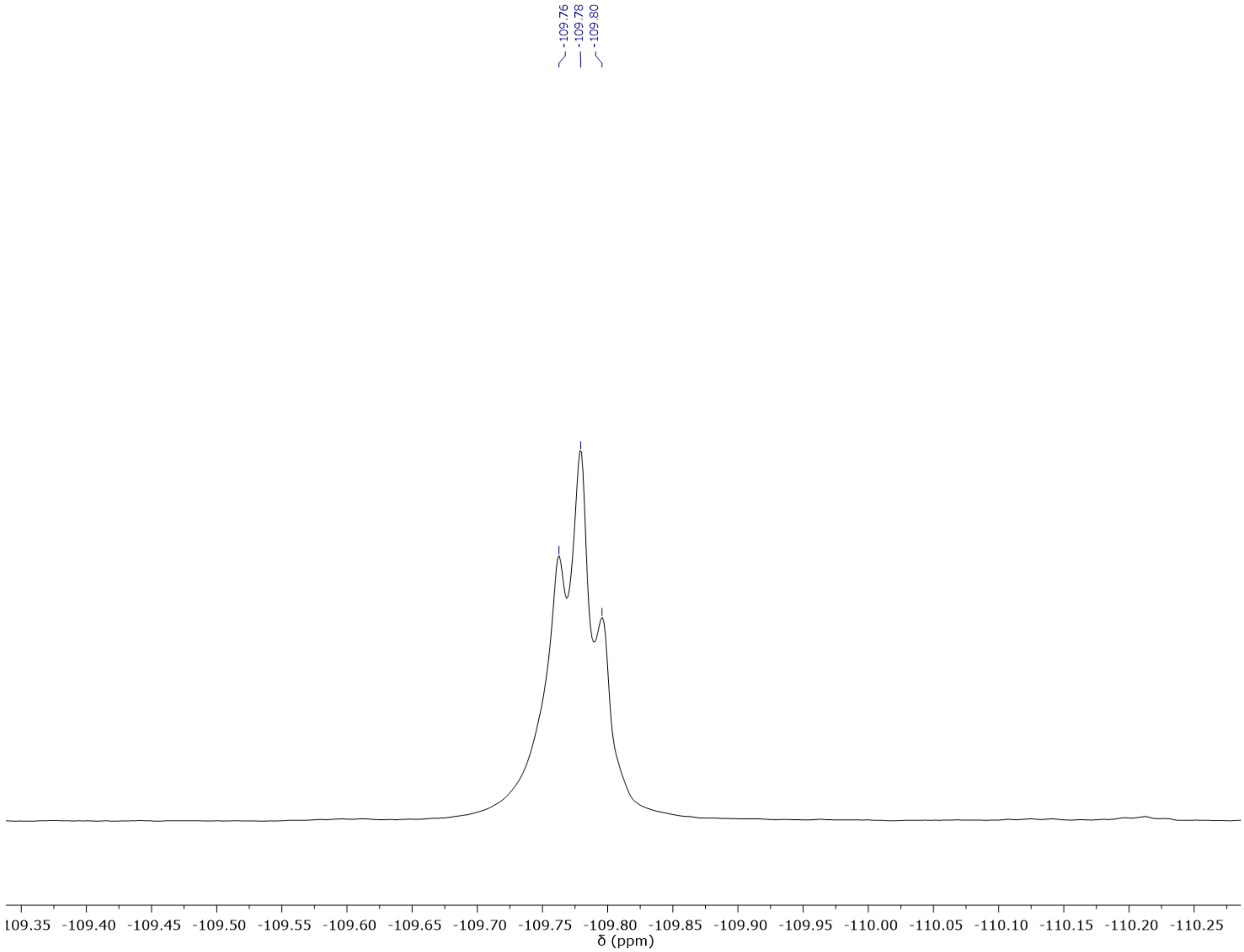

Figure S8. ^{19}F NMR (470 MHz, C_6D_6) of **F_2TrCl**.

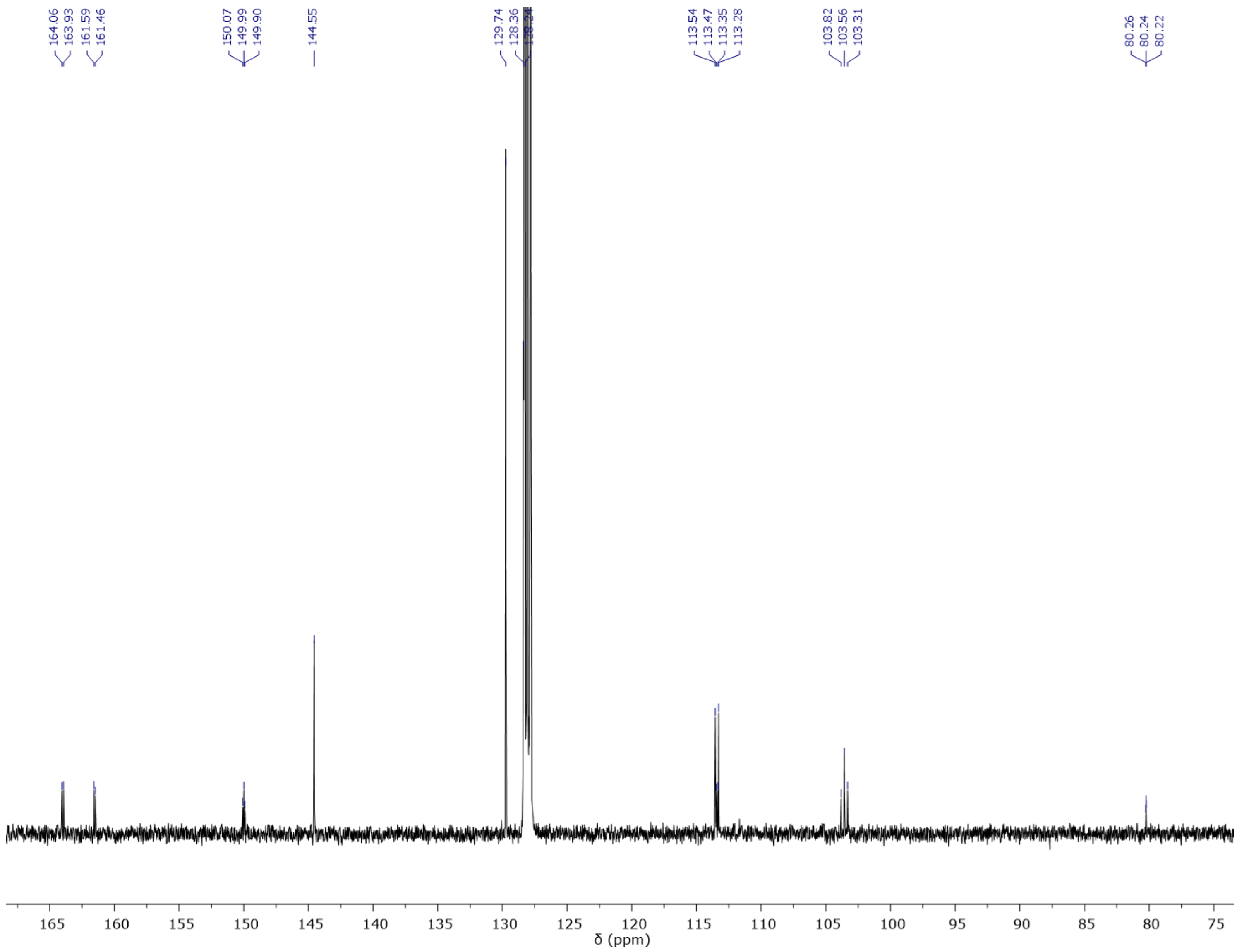

Figure S9. $^{13}C\{^{1}H\}$ NMR (101 MHz, C_6D_6) of **F_2TrCl**.

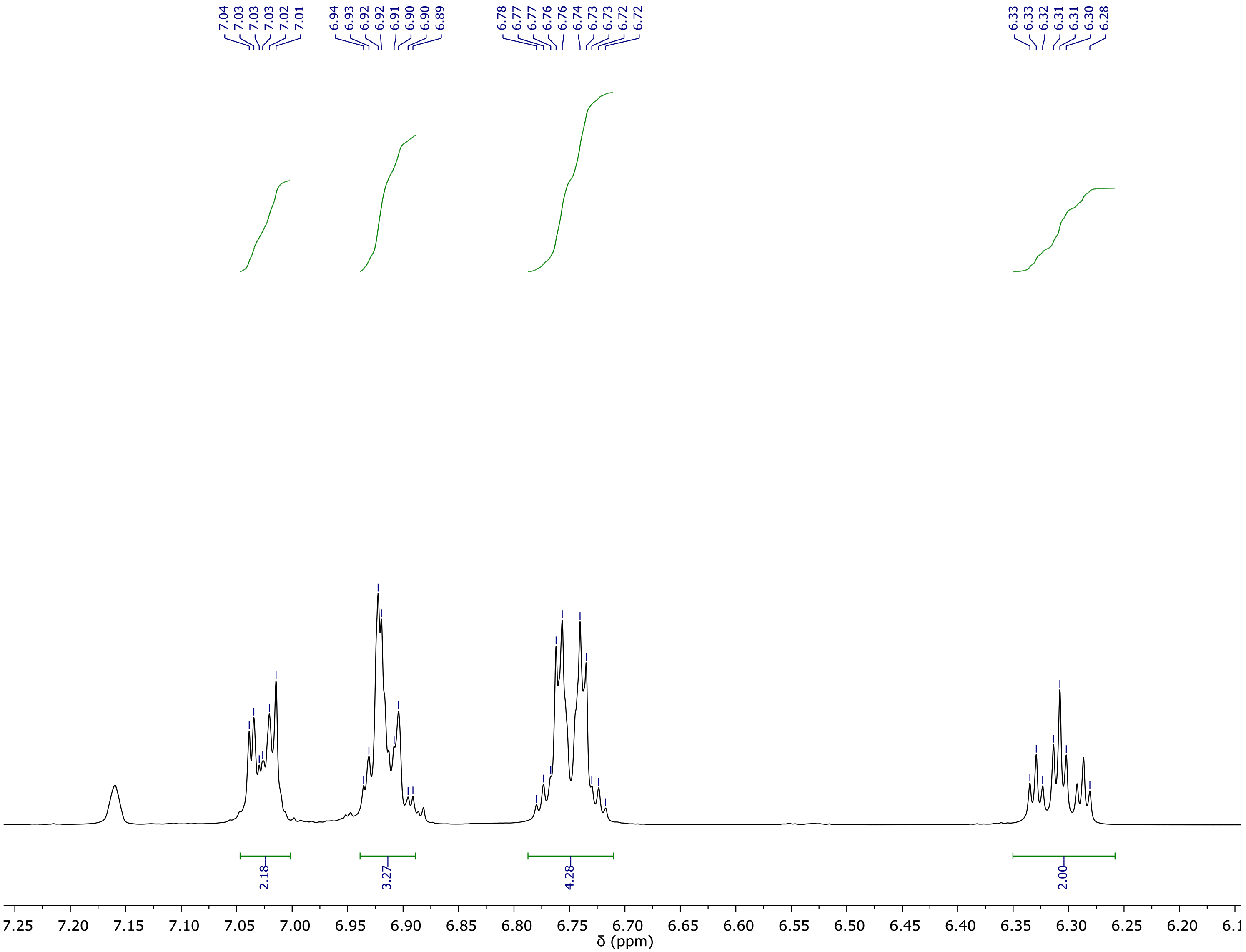

Figure S10. ^{1}H NMR (400 MHz, C_6D_6) spectrum of **F_4TrCl**.

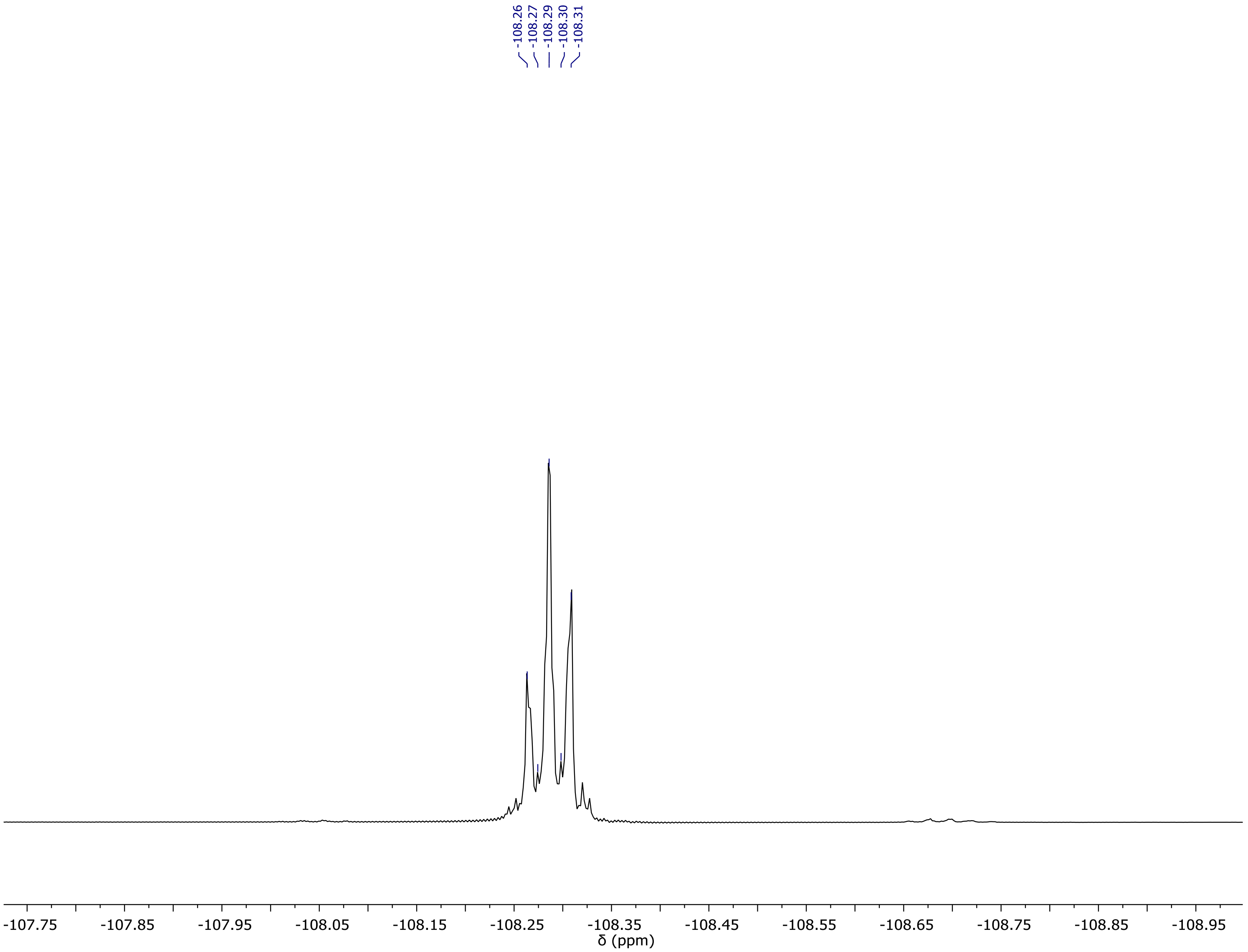

Figure S11. ^{19}F NMR (376 MHz, C_6D_6) spectrum of **F_4TrCl**.

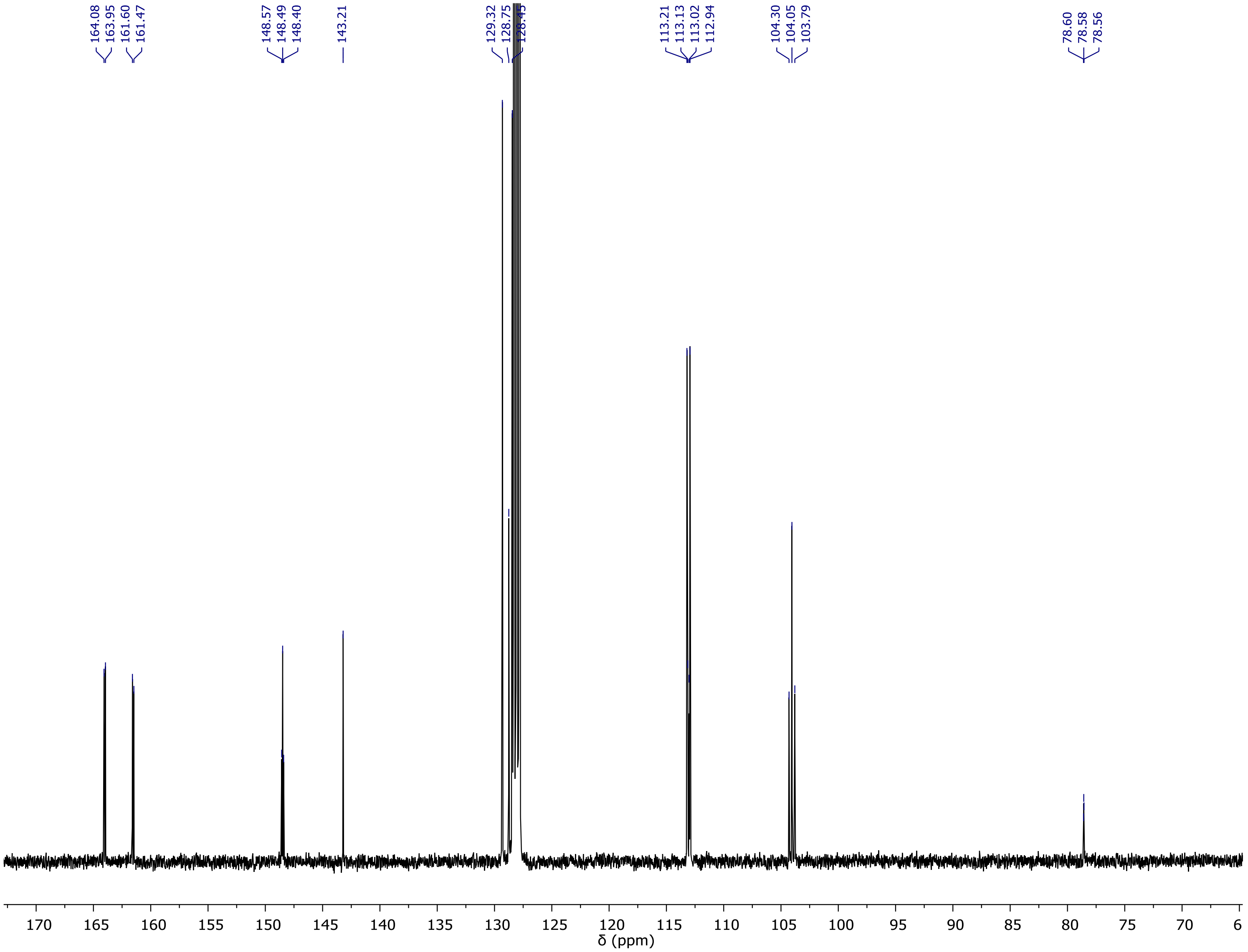

Figure S12. $^{13}C\{^{1}H\}$ NMR (101 MHz, C_6D_6) spectrum of **F_4TrCl**.

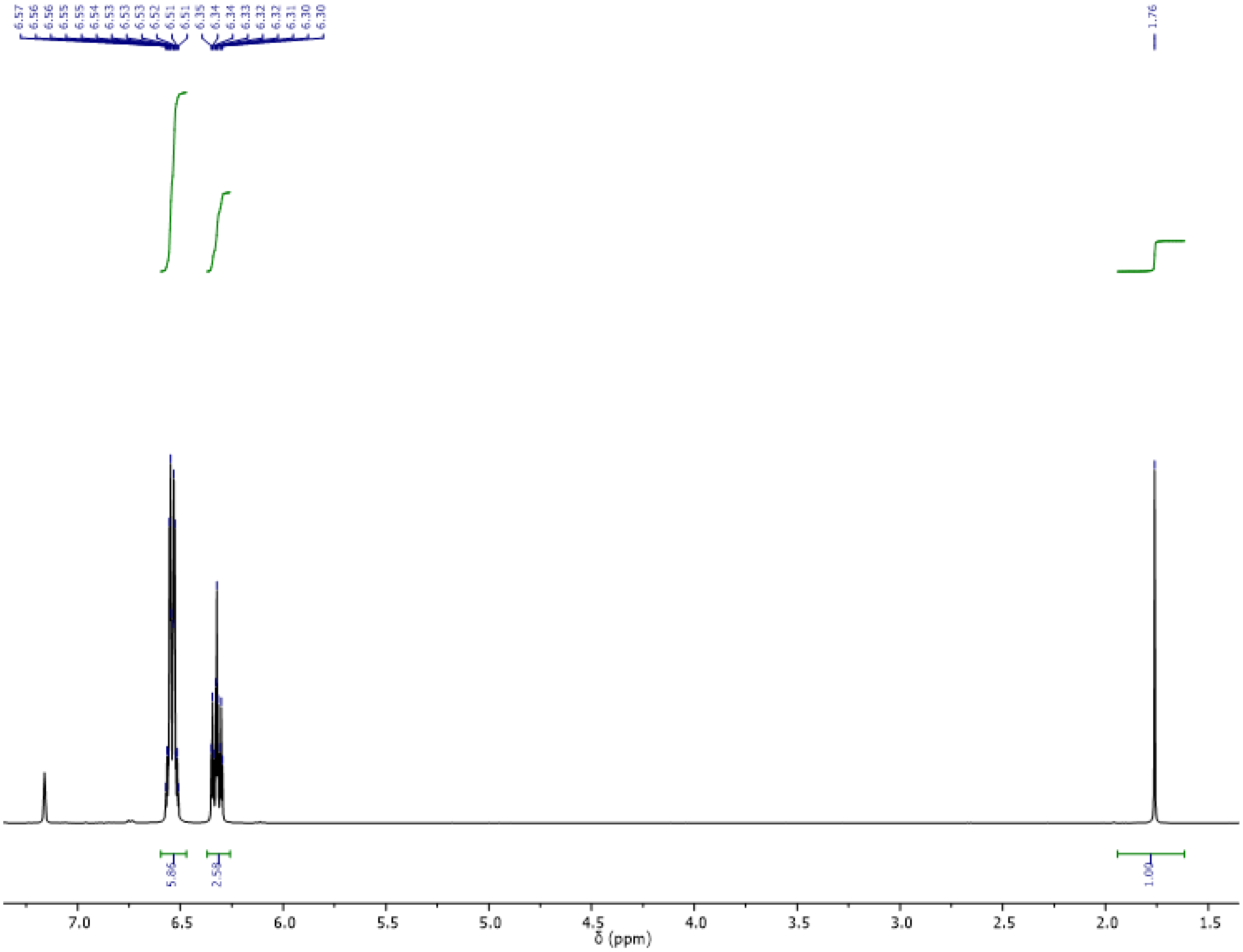

Figure S13. ^{1}H NMR (400 MHz, C_6D_6) spectrum of **F_6TrOH**.

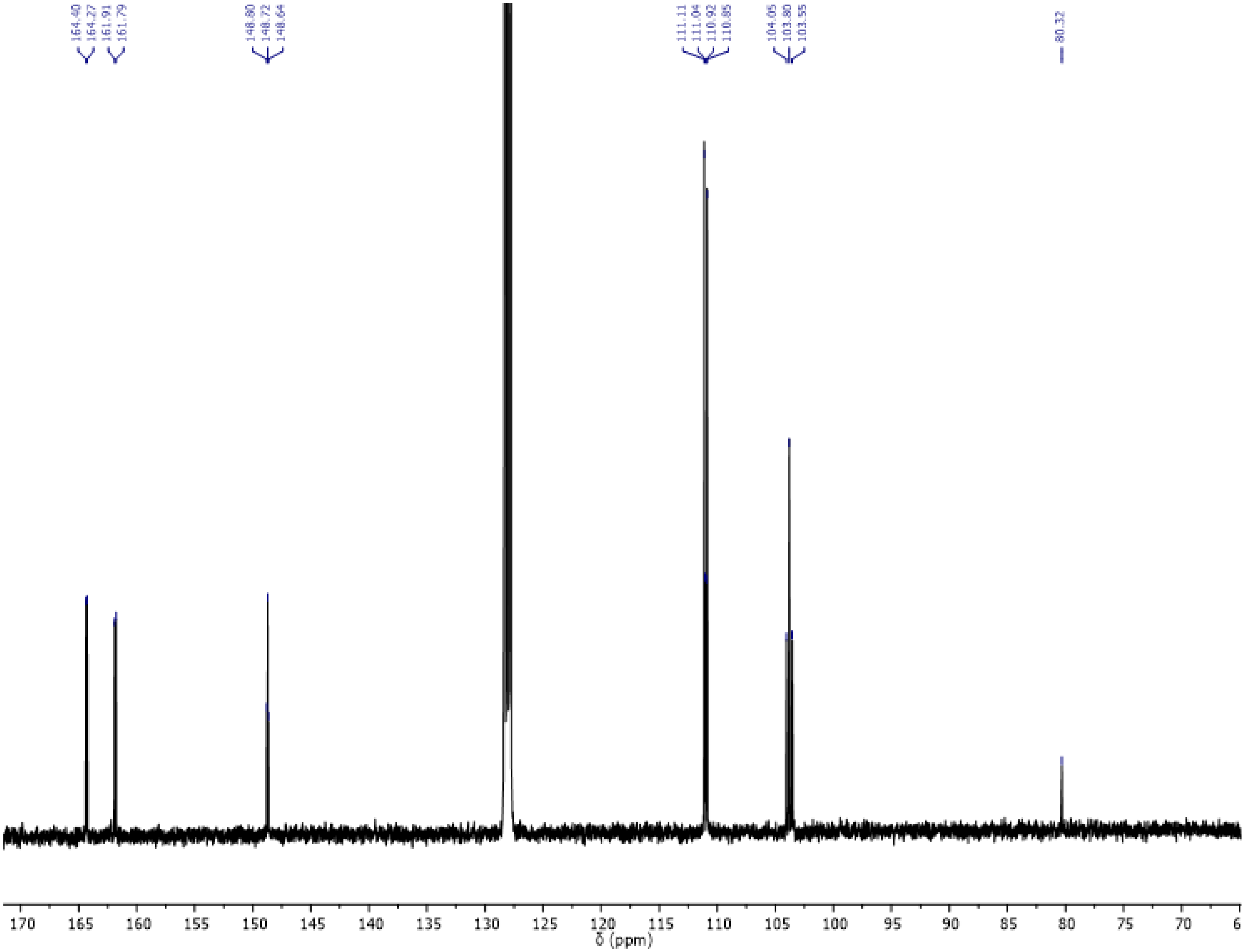

Figure S14. $^{13}C\{^{1}H\}$ NMR (101 MHz, C_6D_6) spectrum of **F_6TrOH**.

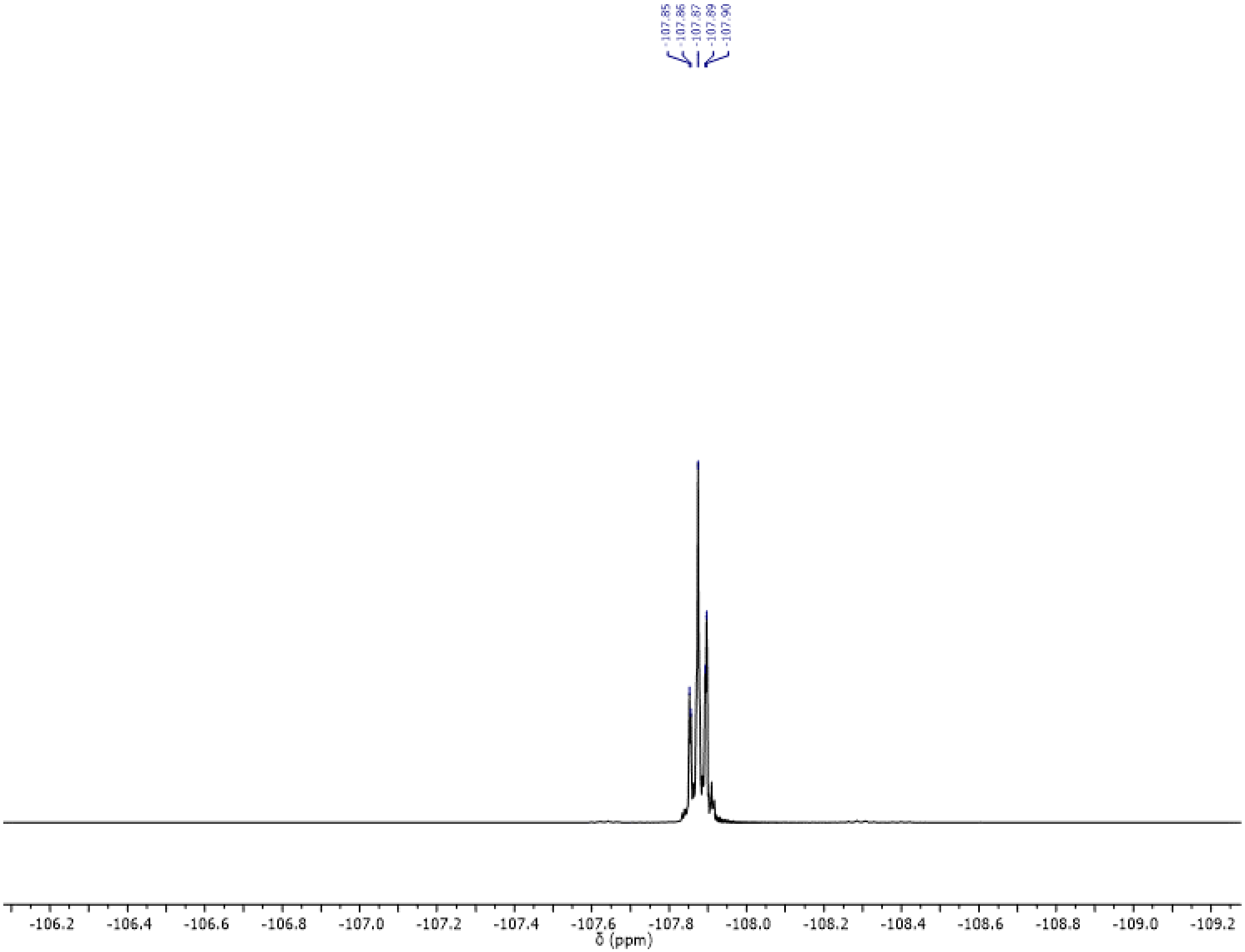

Figure S15. ^{19}F NMR (376 MHz, C_6D_6) spectrum of **F_6TrOH**.

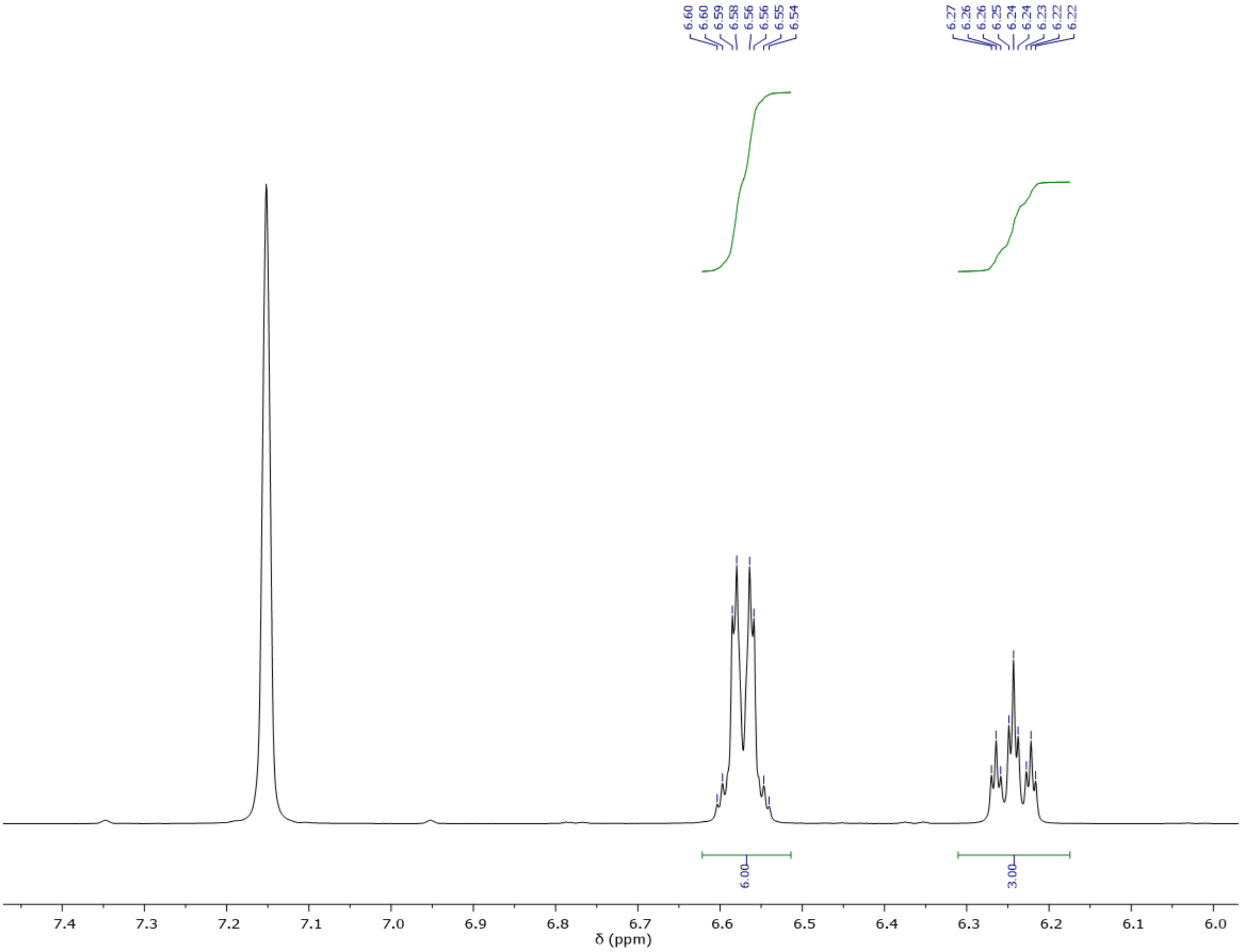

Figure S16. ^{1}H NMR (400 MHz, C_6D_6) spectrum of **F_6TrCl.**

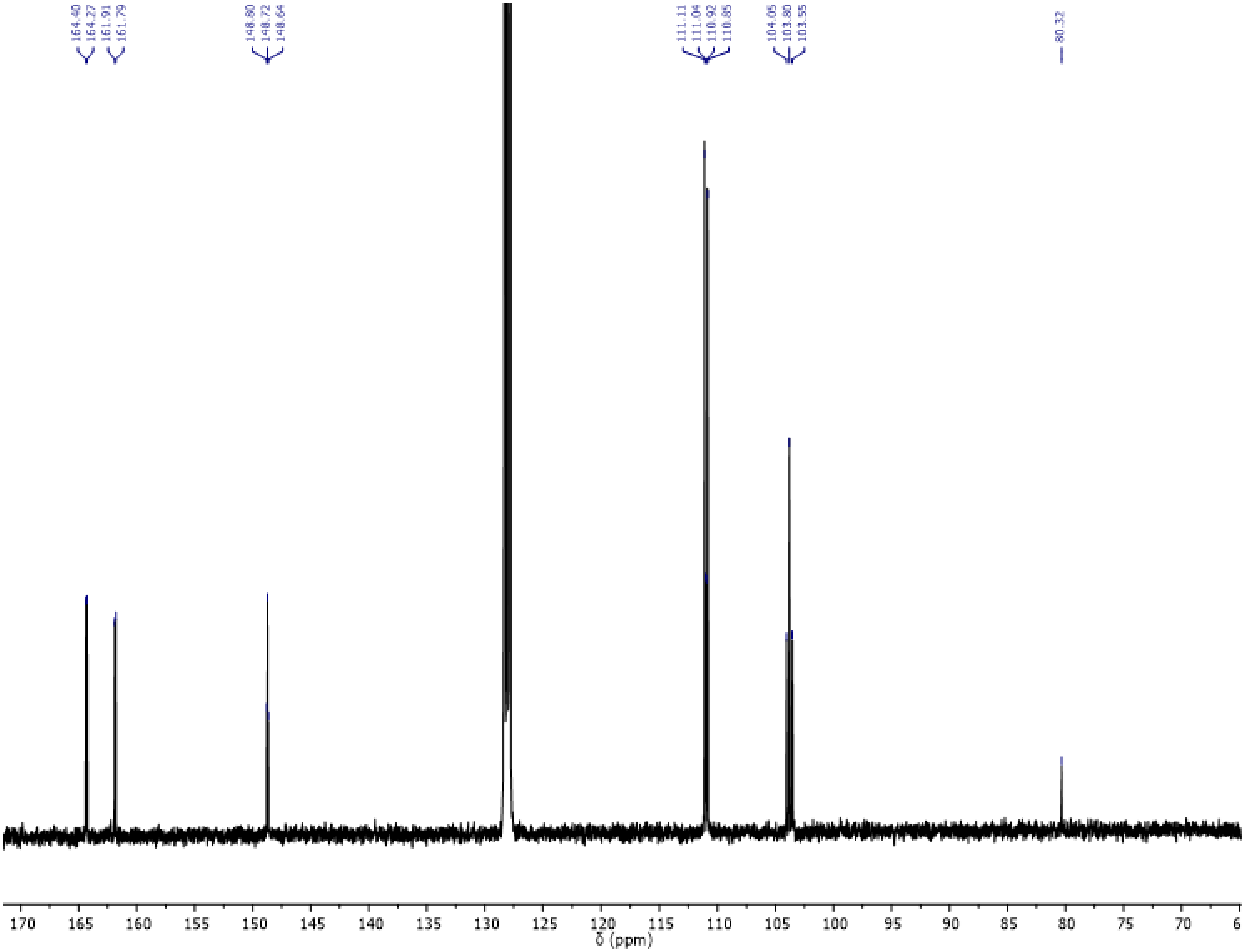

Figure S17. $^{13}C\{^{1}H\}$ NMR spectrum (101 MHz, C_6D_6) of **F_6TrCl.**

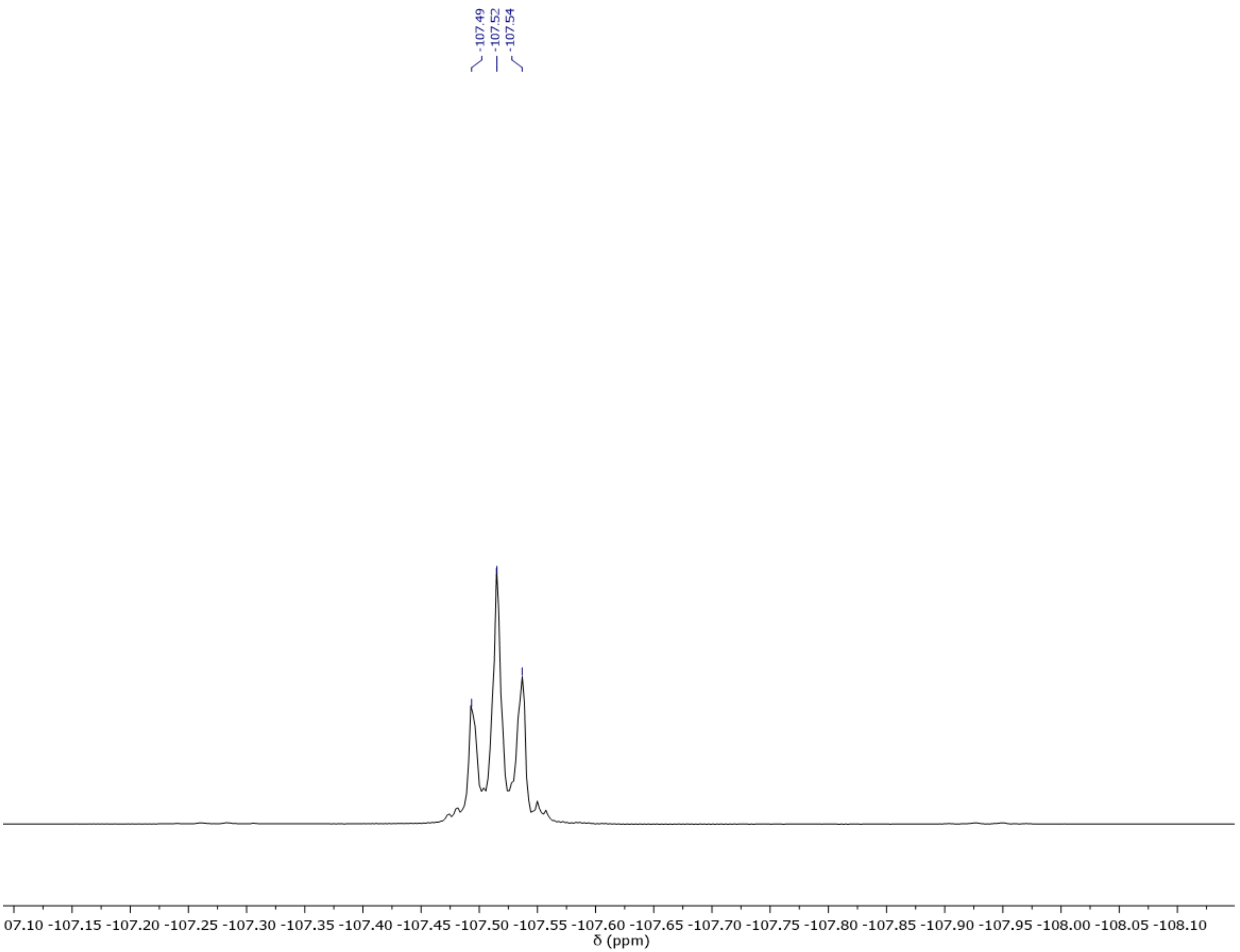

Figure S18. ^{19}F NMR (376 MHz, C_6D_6) spectrum of **F6TrCl**.

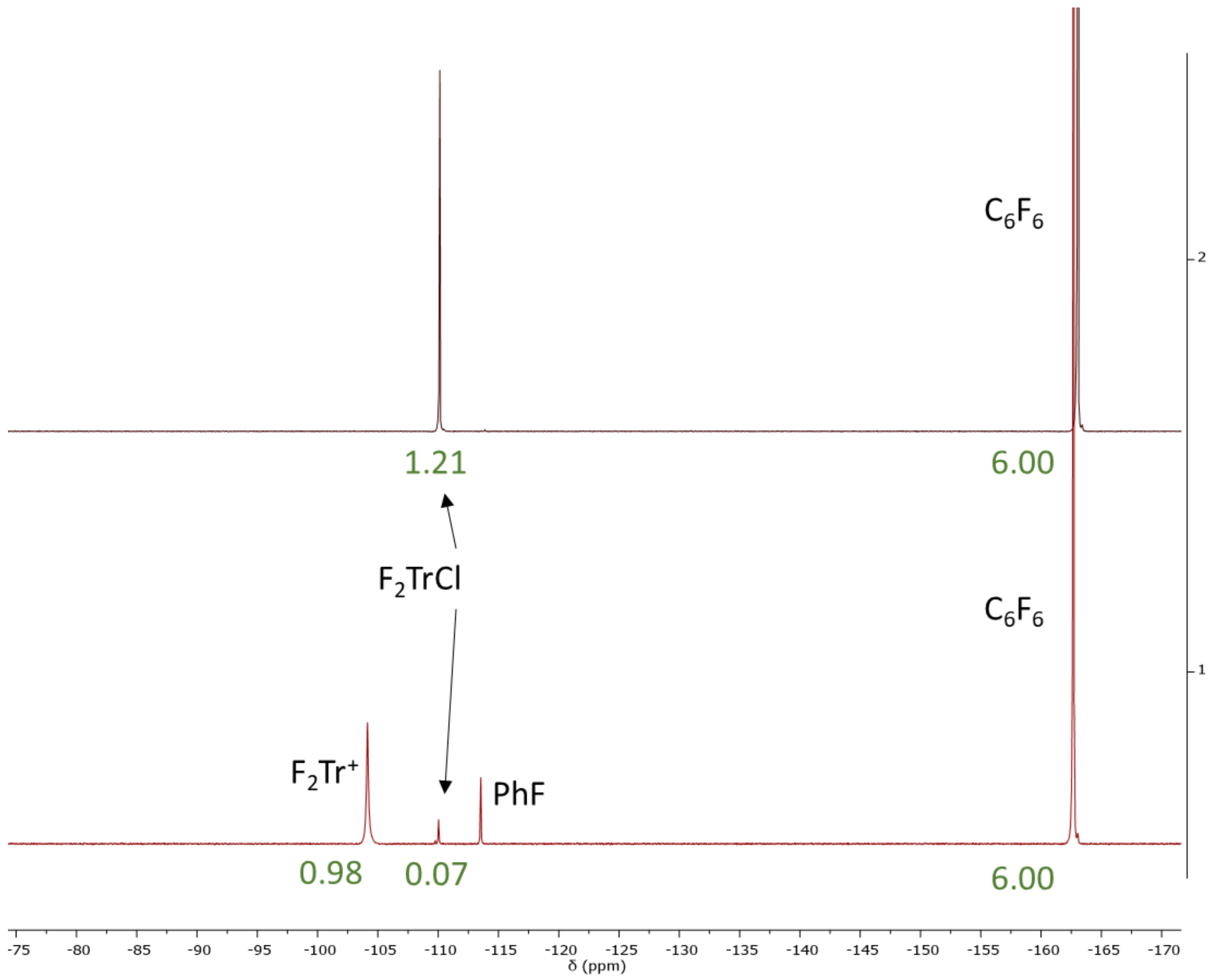

Figure S19. ^{19}F NMR (470 MHz, *o*-$C_6H_4Cl_2$) spectrum of **F_2TrCl** before (top) and after the addition of 1 eq of **Na[Cl11]** (bottom) with C_6F_6 as an internal standard (**Na**).

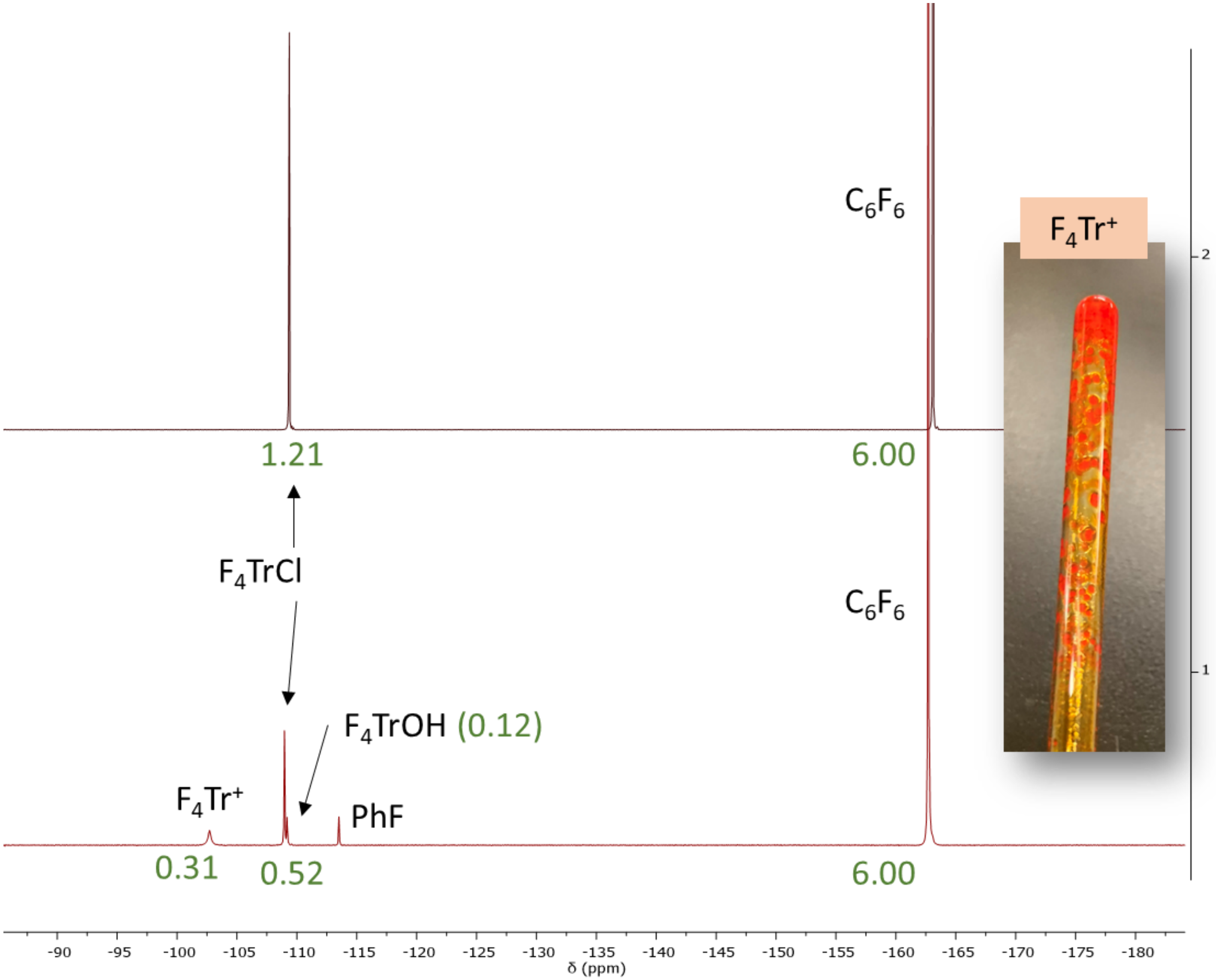

Figure S20. ^{19}F NMR (470 MHz, *o*-$C_6H_4Cl_2$) spectrum of **F_4TrCl** (top) and 24 h after the addition of 1 eq of **Na[Cl11]** (bottom) with C_6F_6 as an internal standard. Crystal deposited on the sides of the J. Young NMR tube (inset, **Na**).

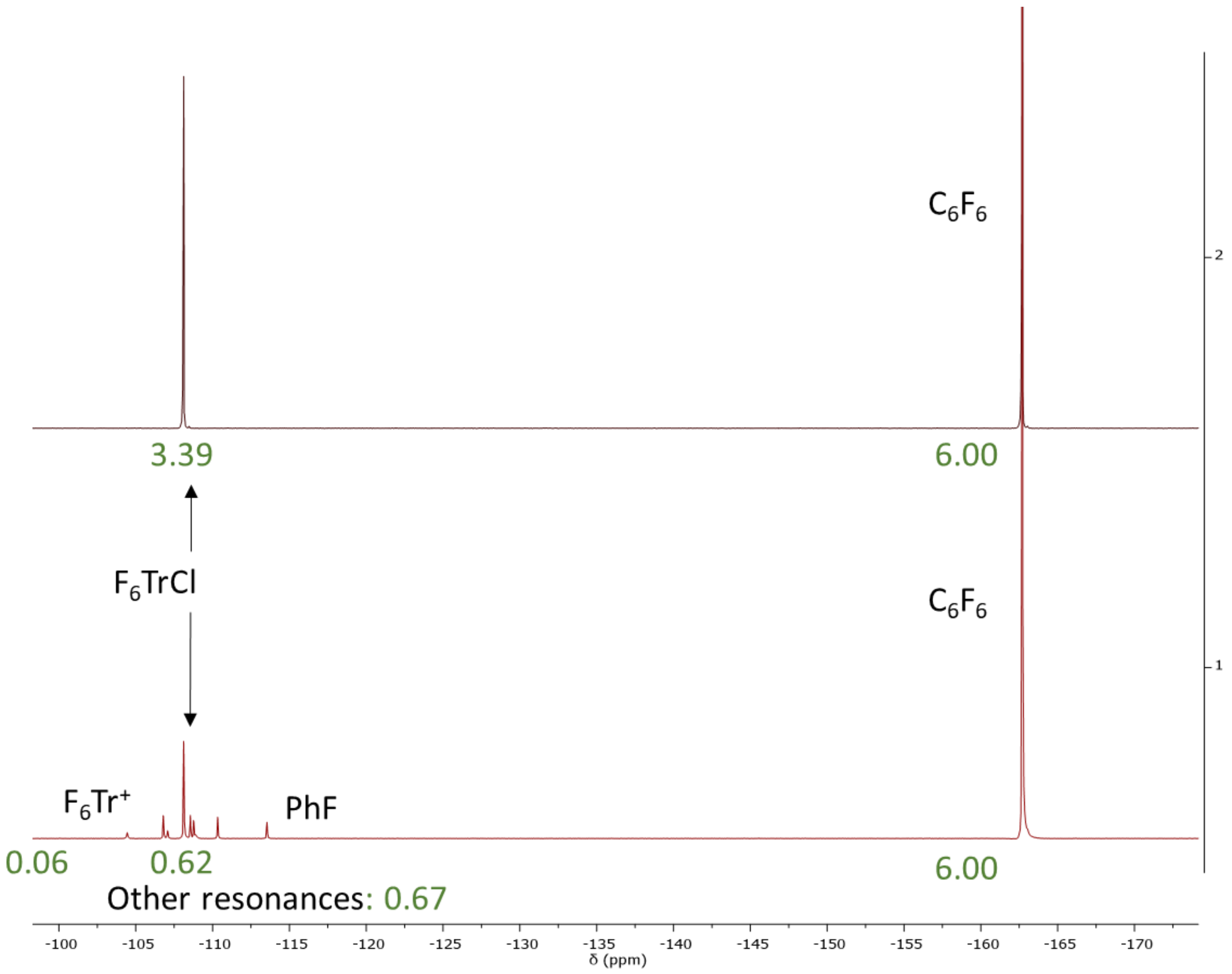

Figure S21. ^{19}F NMR (470 MHz, *o*-$C_6H_4Cl_2$) spectrum of **F_6TrCl** before (top) and after the addition of 1 eq of **Na[Cl11]** (bottom) with C_6F_6 as an internal standard (**Na**).

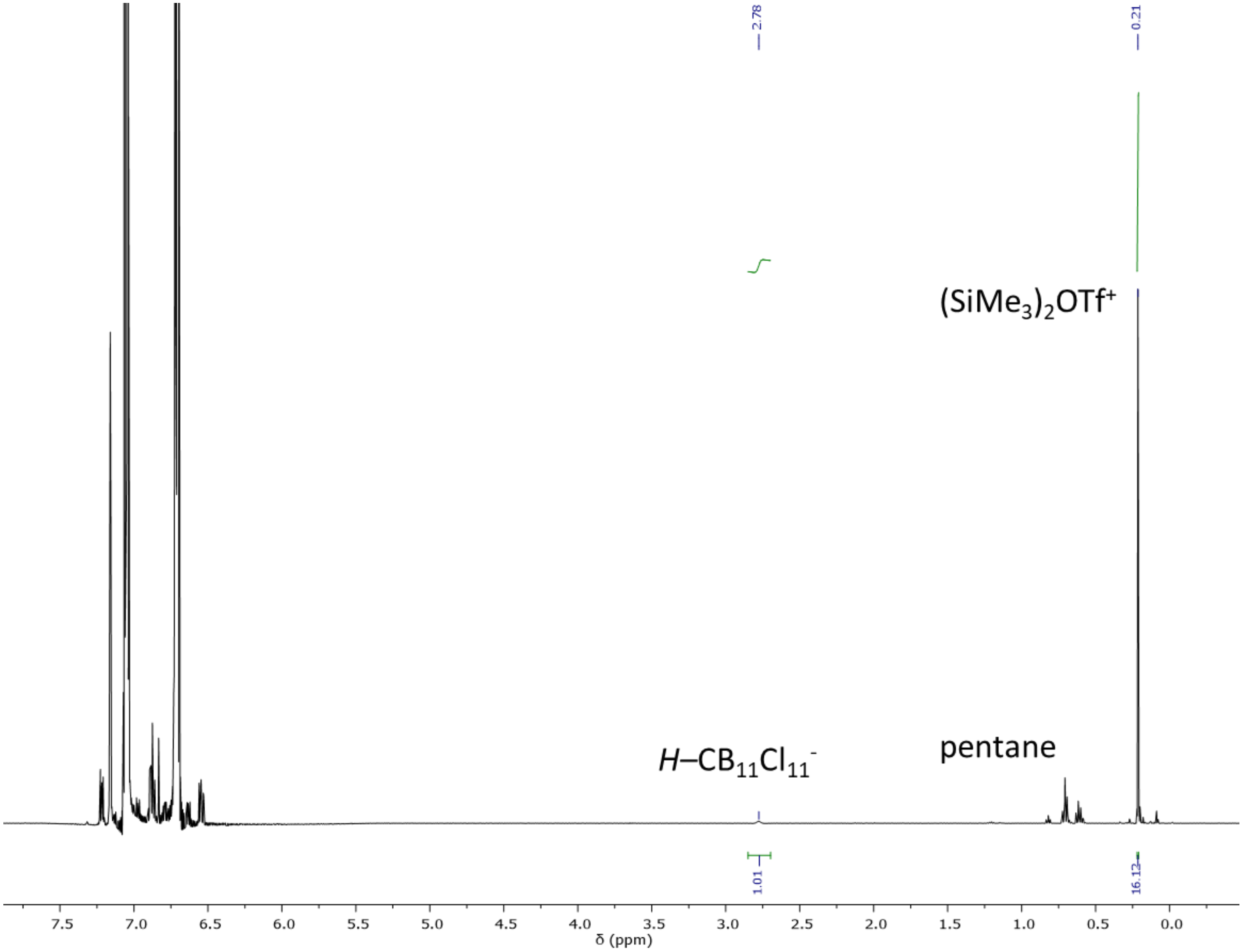

Figure S22. ^{1}H NMR (500 MHz, C_6D_6/*o*-$C_6H_4Cl_2$) spectrum of **[$(SiMe_3)_2$OTf][Cl11]**. Residual pentane is also observed (**Si**).

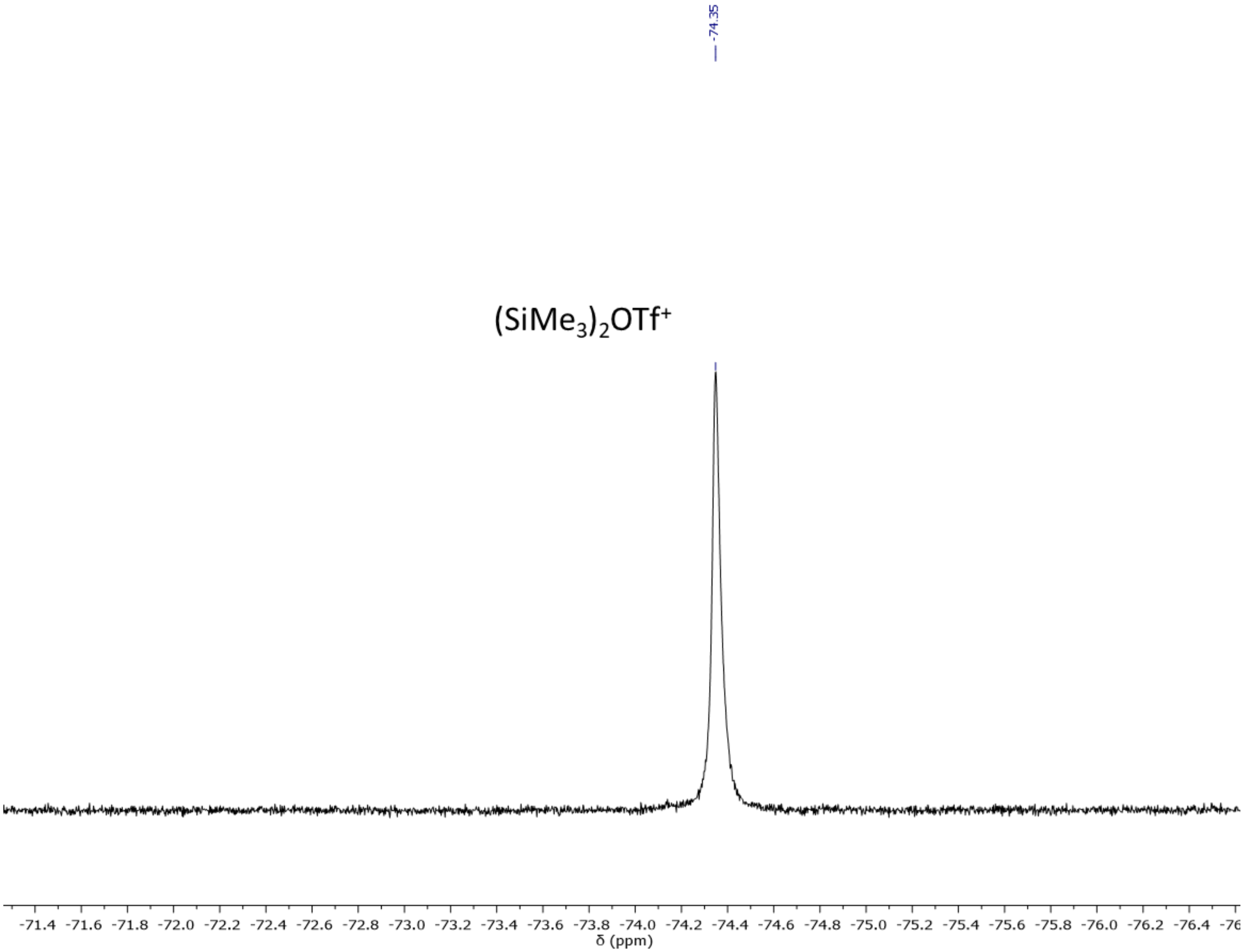

Figure S23. ^{19}F NMR (470 MHz, C_6D_6/*o*-$C_6H_4Cl_2$) spectrum of **[(SiMe$_3$)$_2$OTf][Cl11]** (**Si**).

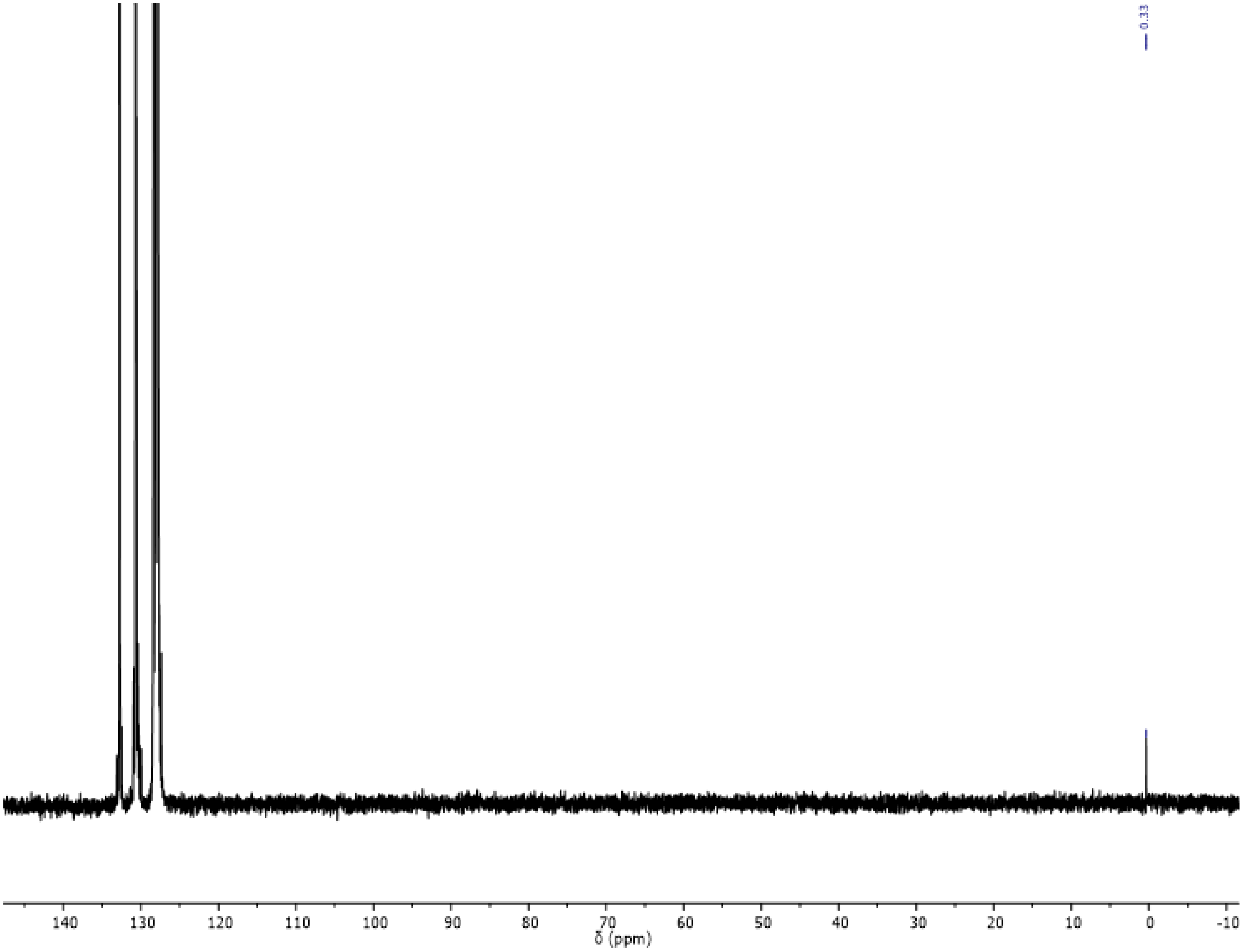

Figure S24. $^{13}C\{^{1}H\}$ NMR (101 MHz, C_6D_6/*o*-$C_6H_4Cl_2$) spectrum of **[(SiMe$_3$)$_2$OTf][Cl11]**.

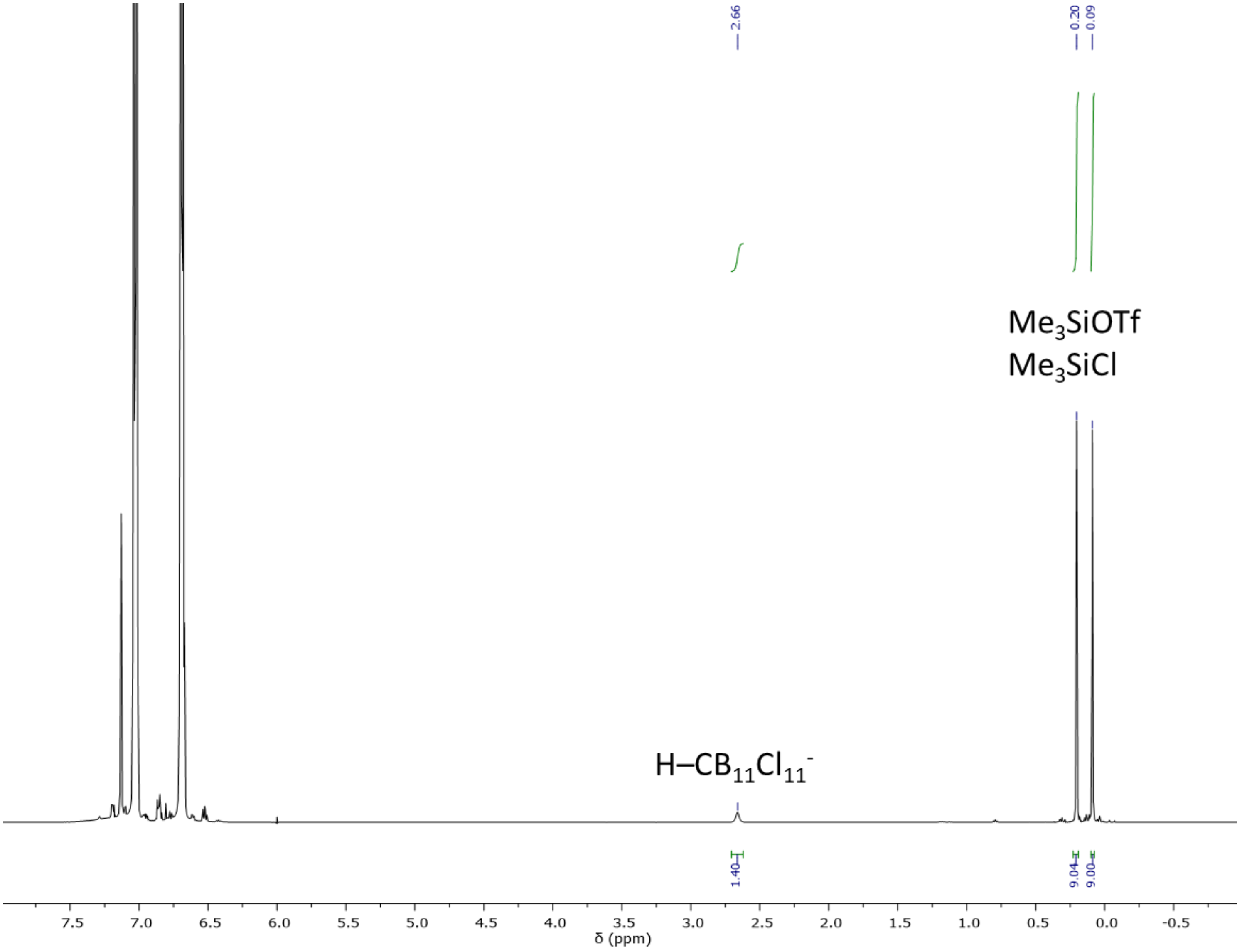

Figure S25. ^{1}H NMR (500 MHz, C_6D_6/*o*-$C_6H_4Cl_2$) spectrum of a reaction of **F_2TrCl** with 1 eq of **[$(SiMe_3)_2$OTf][Cl11]**. A 1:1 ratio of Me_3SiOTf and Me_3SiCl is observed.

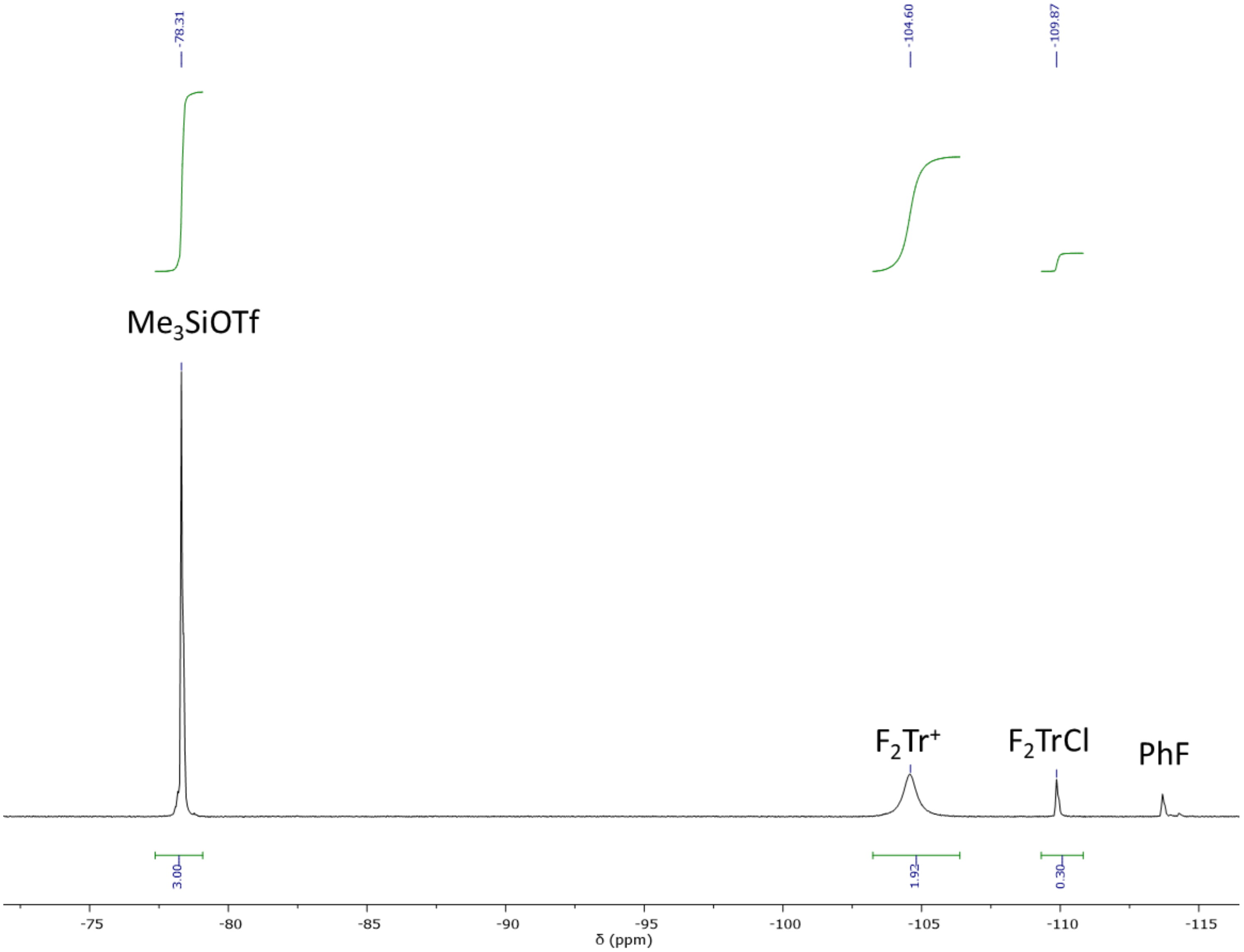

Figure S26. ^{19}F NMR (470 MHz, C_6D_6/*o*-$C_6H_4Cl_2$) spectrum of a reaction of **F_2TrCl** with 1 eq of **[$(SiMe_3)_2$OTf][Cl11].** Residual starting materials and PhF are also observed.

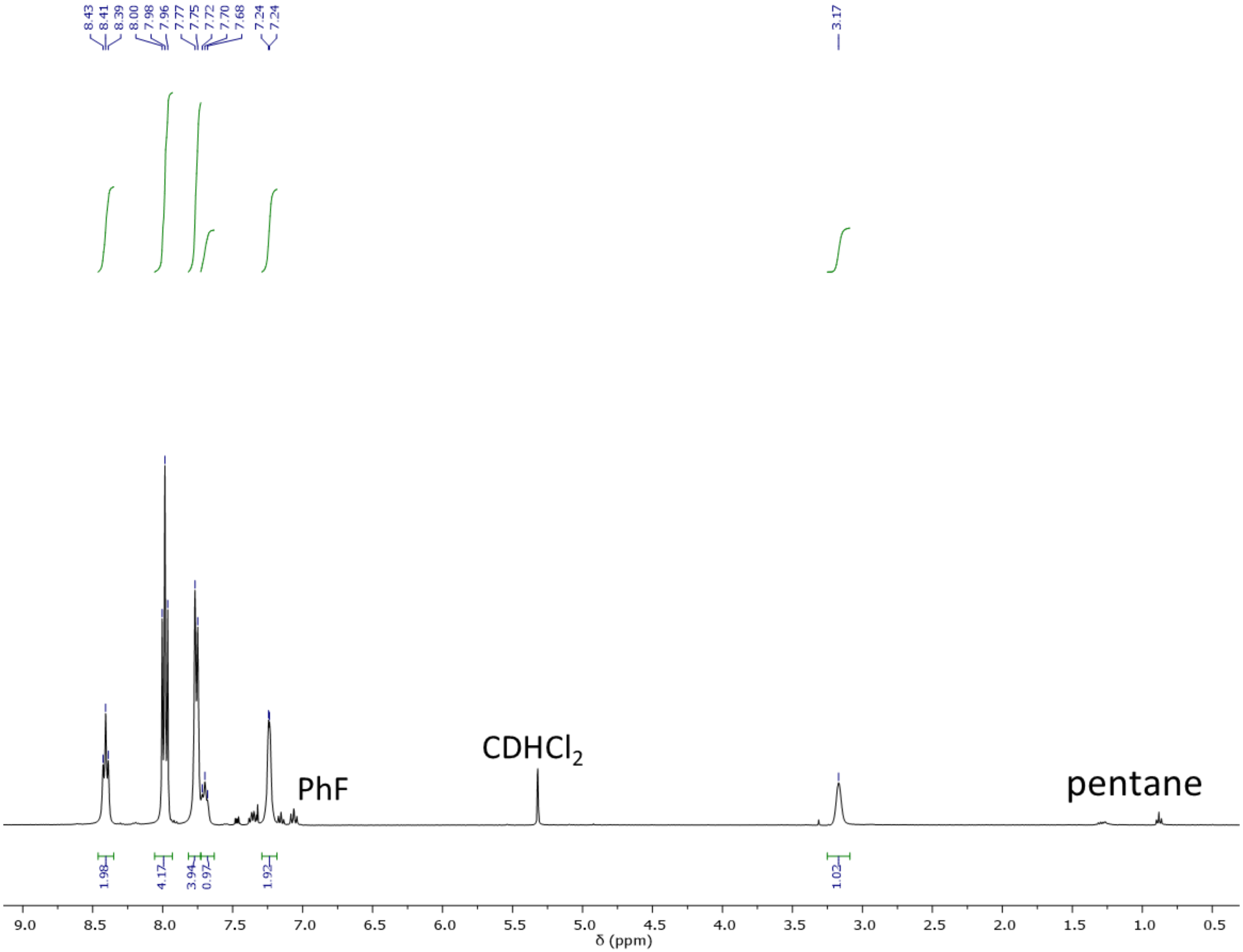

Figure S27. ^{1}H NMR (400 MHz, CD_2Cl_2) spectrum of **F_2Tr[Cl11]**. Residual pentane and PhF are also observed.

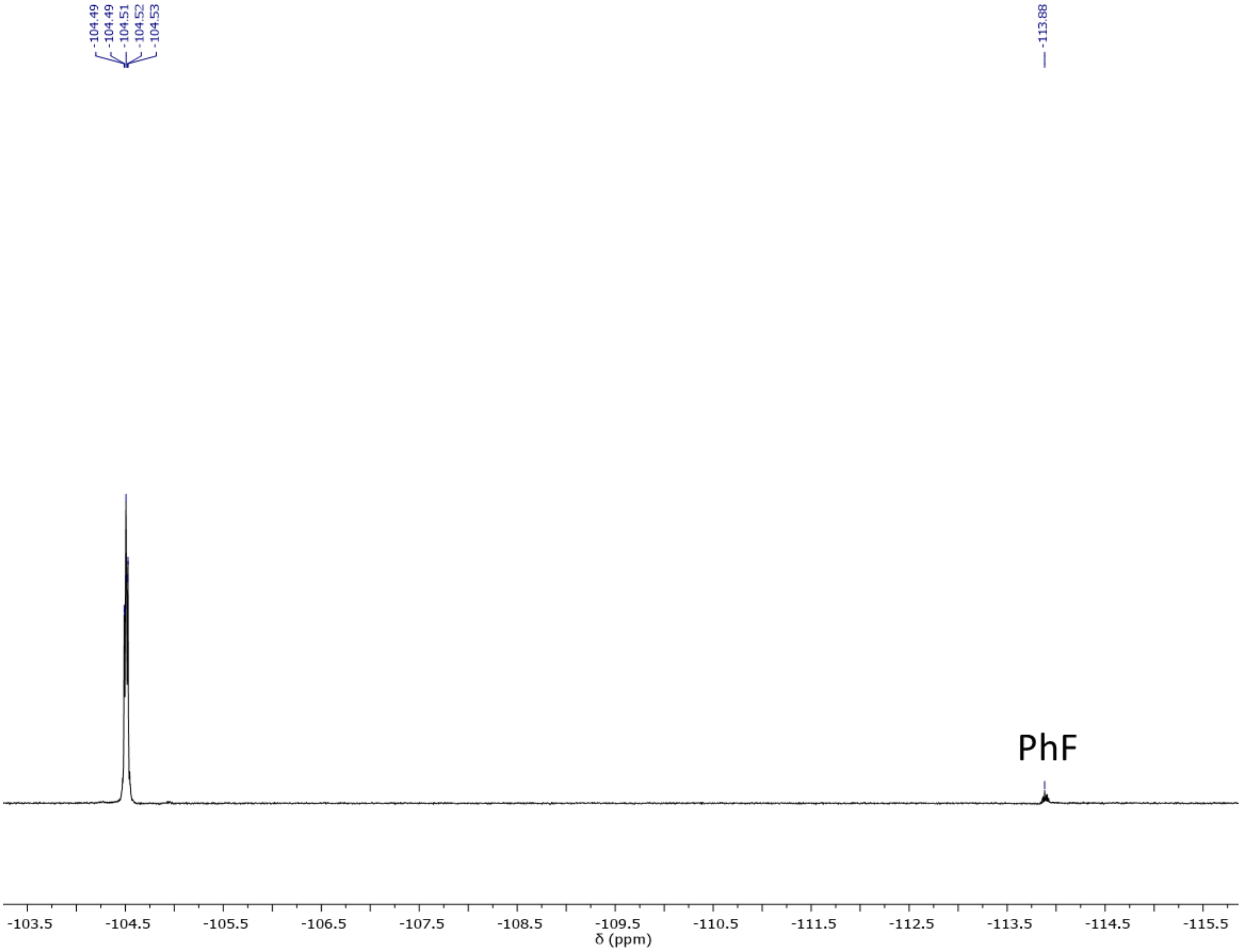

Figure S28. ^{19}F NMR (376 MHz, CD_2Cl_2) spectrum of **F_2Tr[Cl11]**. Residual PhF is also observed.

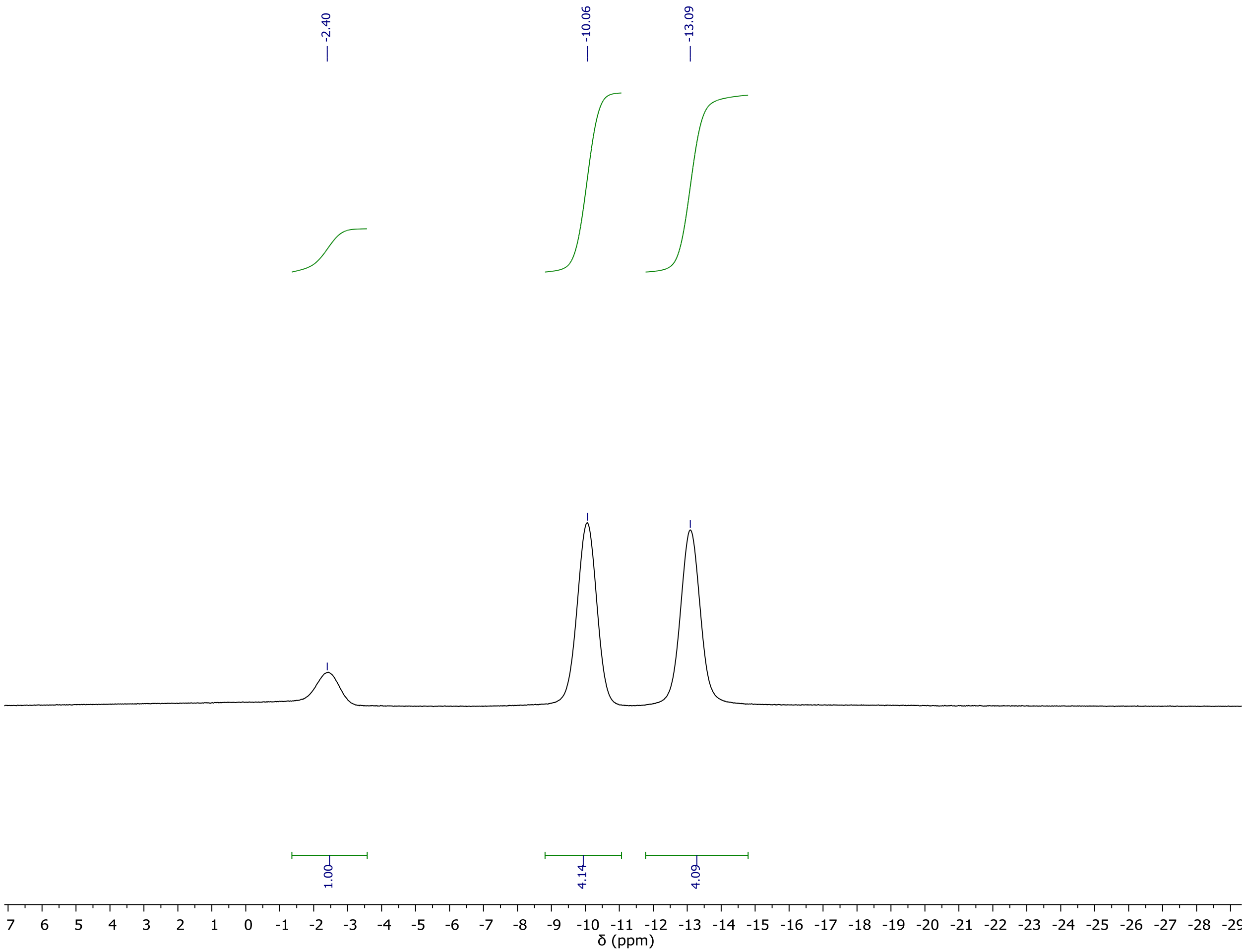

Figure S29. ^{11}B NMR (128 MHz, CD_2Cl_2) spectrum of **F_2Tr[Cl11]**.

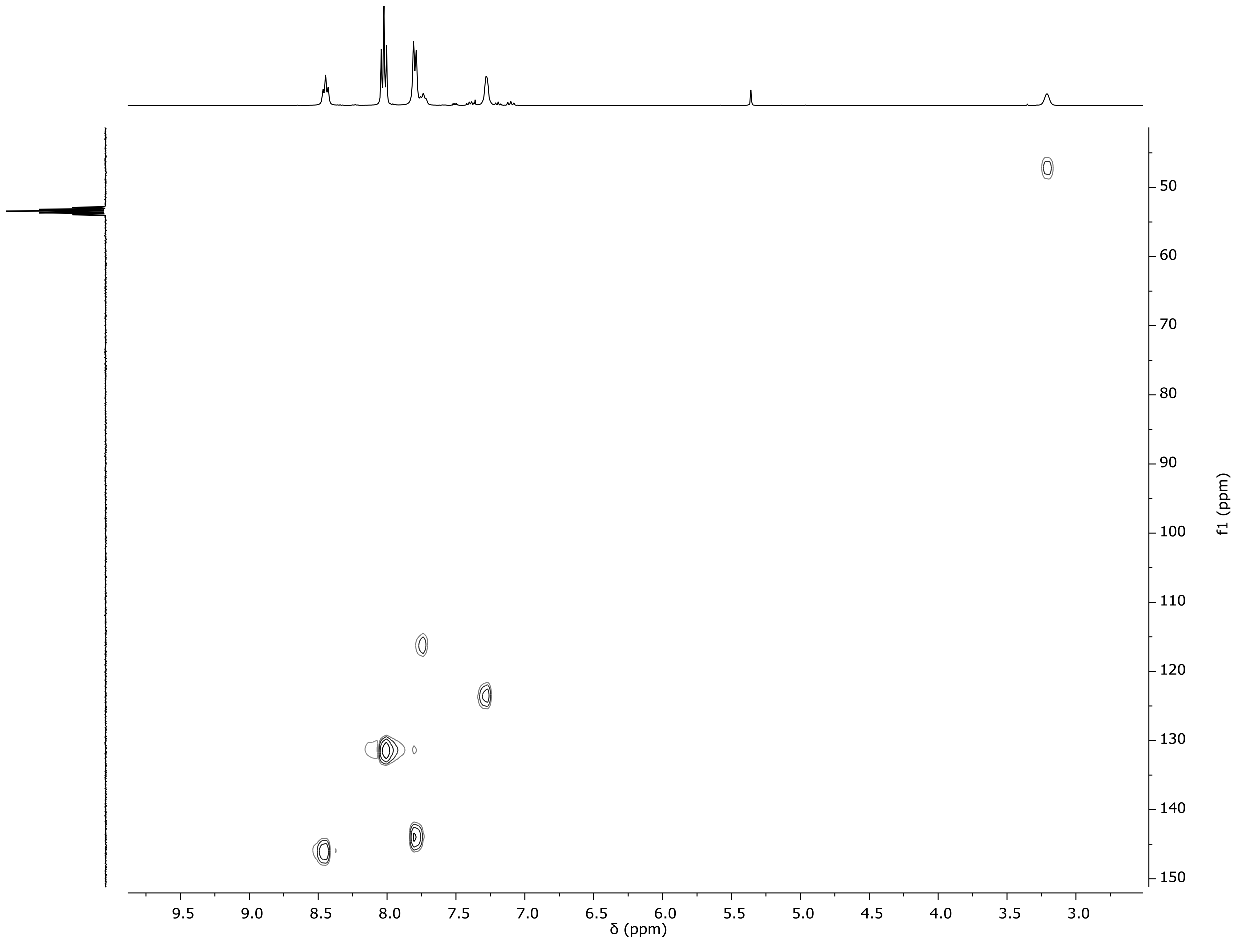

Figure S30. ^{13}C-^{1}H HSQC NMR (400 MHz, CD_2Cl_2) spectrum of **F_2Tr[Cl11]**.

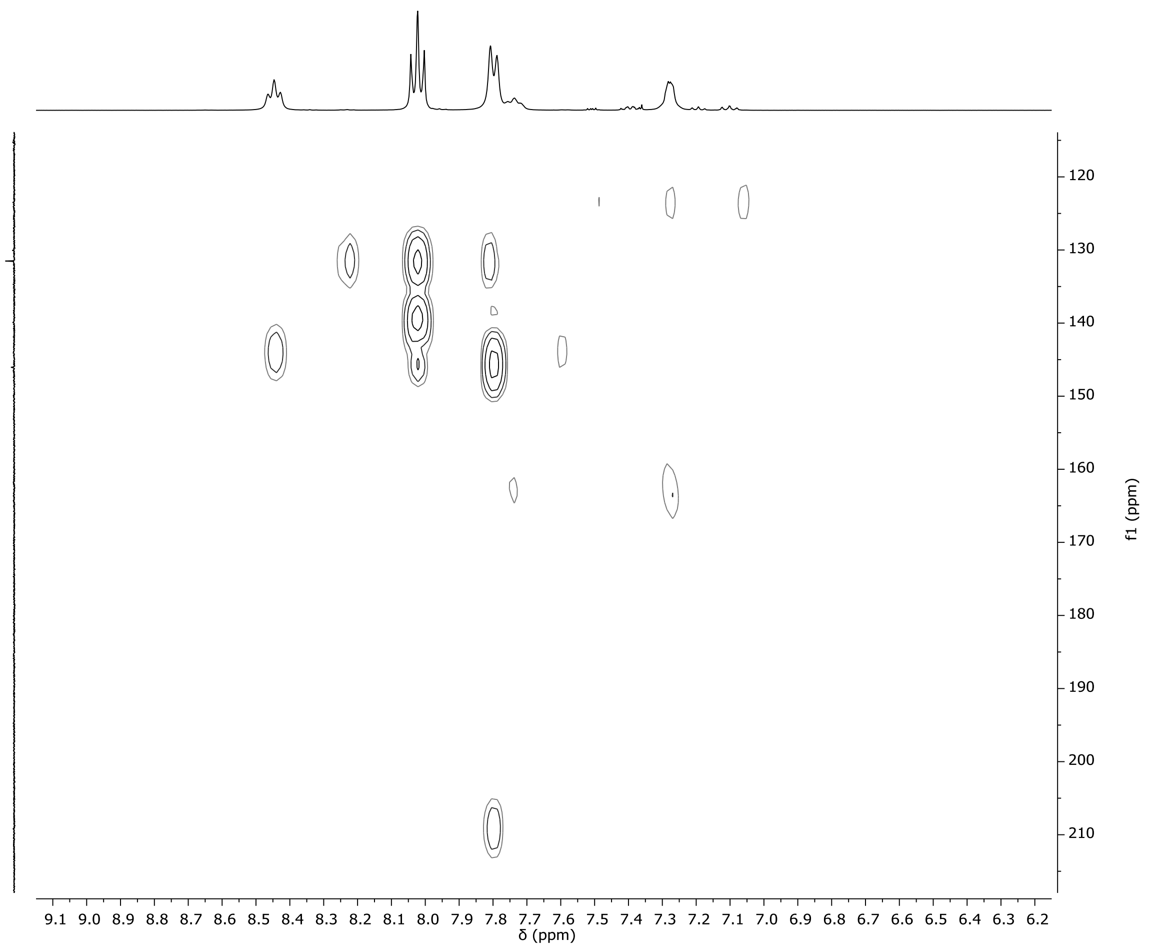

Figure S31. ^{13}C-^{1}H HMBC NMR (400 MHz, CD_2Cl_2) spectrum of **F_2Tr[Cl11]**.

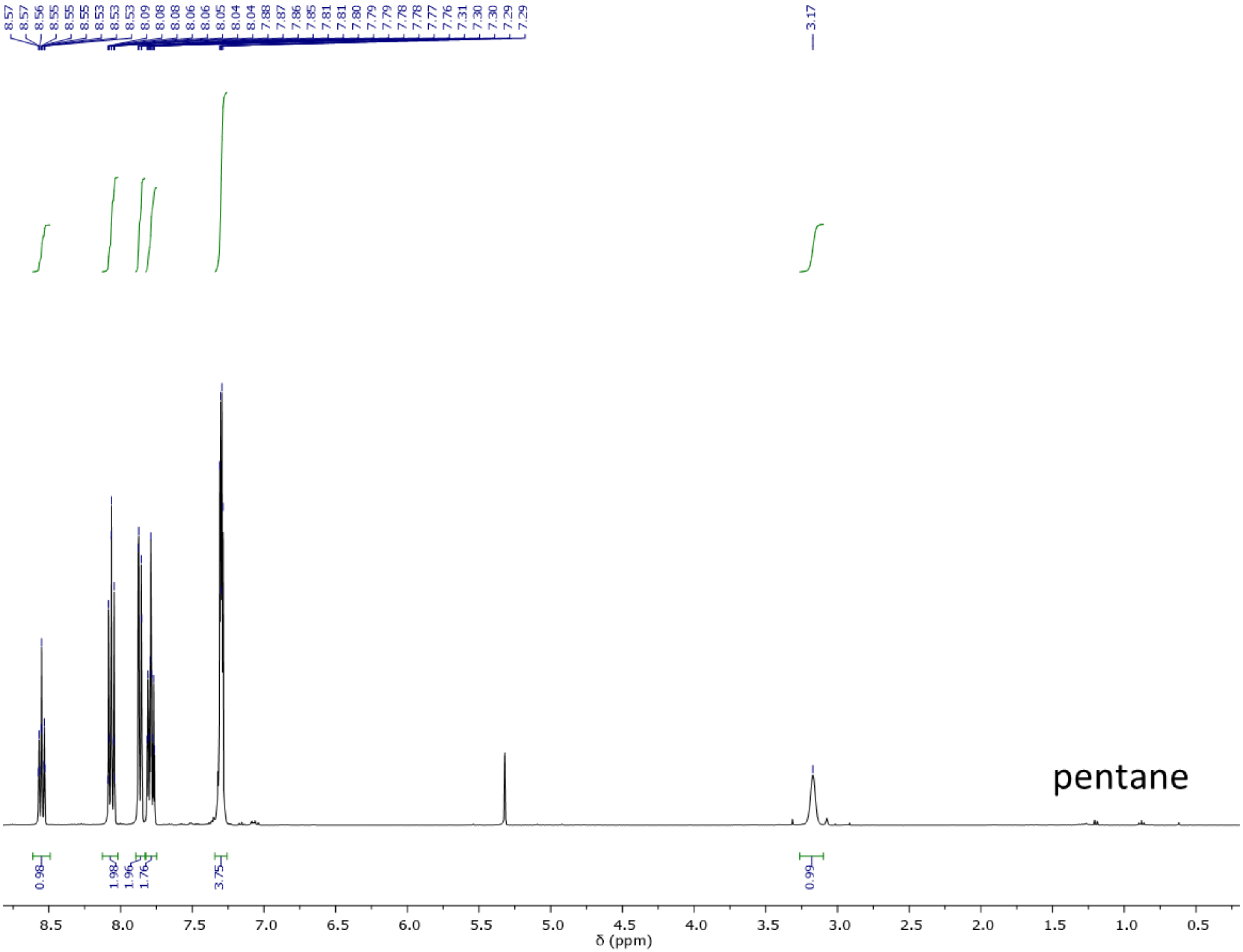

Figure S32. Full ^{1}H NMR (400 MHz, CD_2Cl_2) spectrum of **F_4Tr[Cl11]**. Residual pentane is observed.

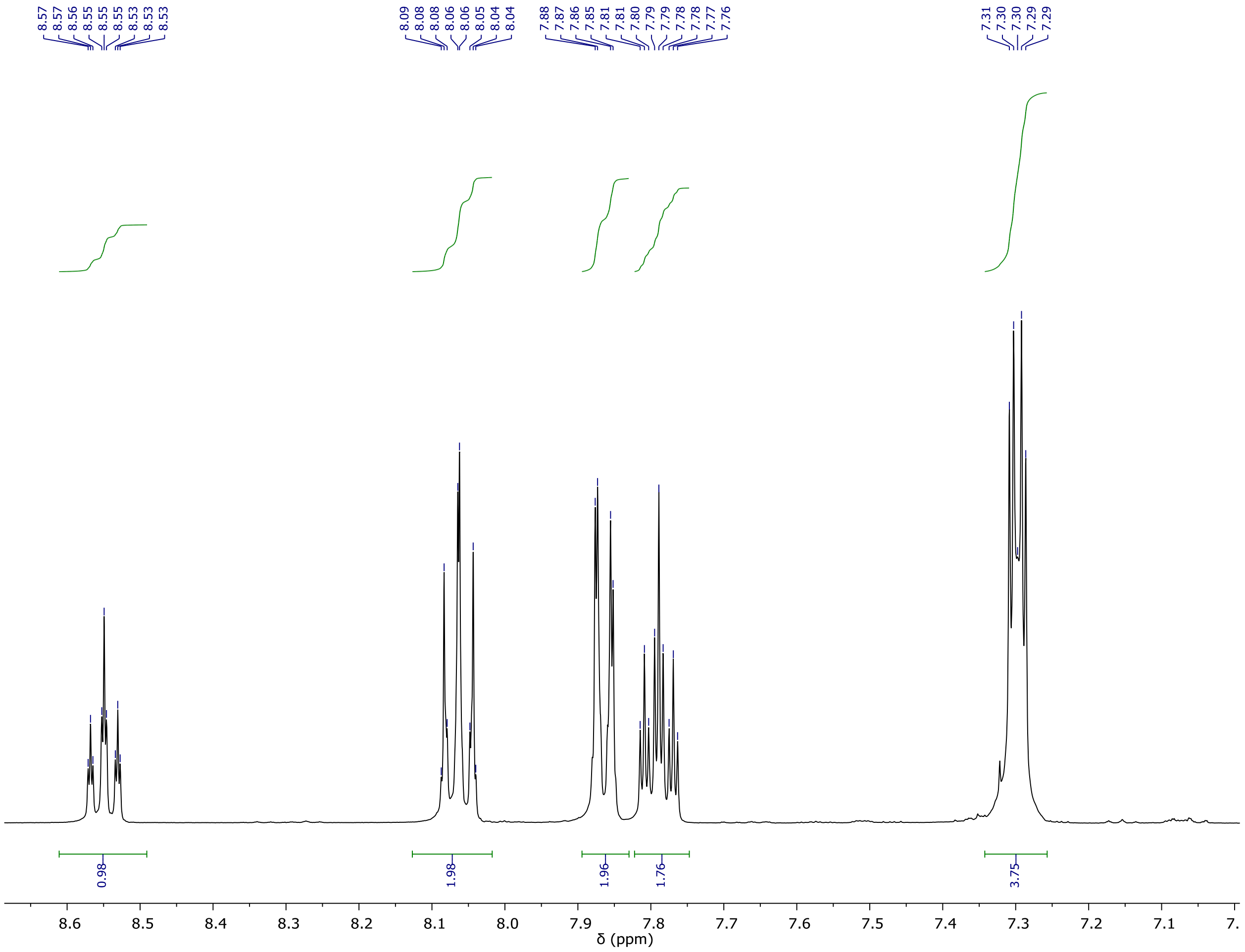

Figure S33. The aromatic region of a ^{1}H NMR (400 MHz, CD_2Cl_2) spectrum of **F_4Tr[Cl11]**.

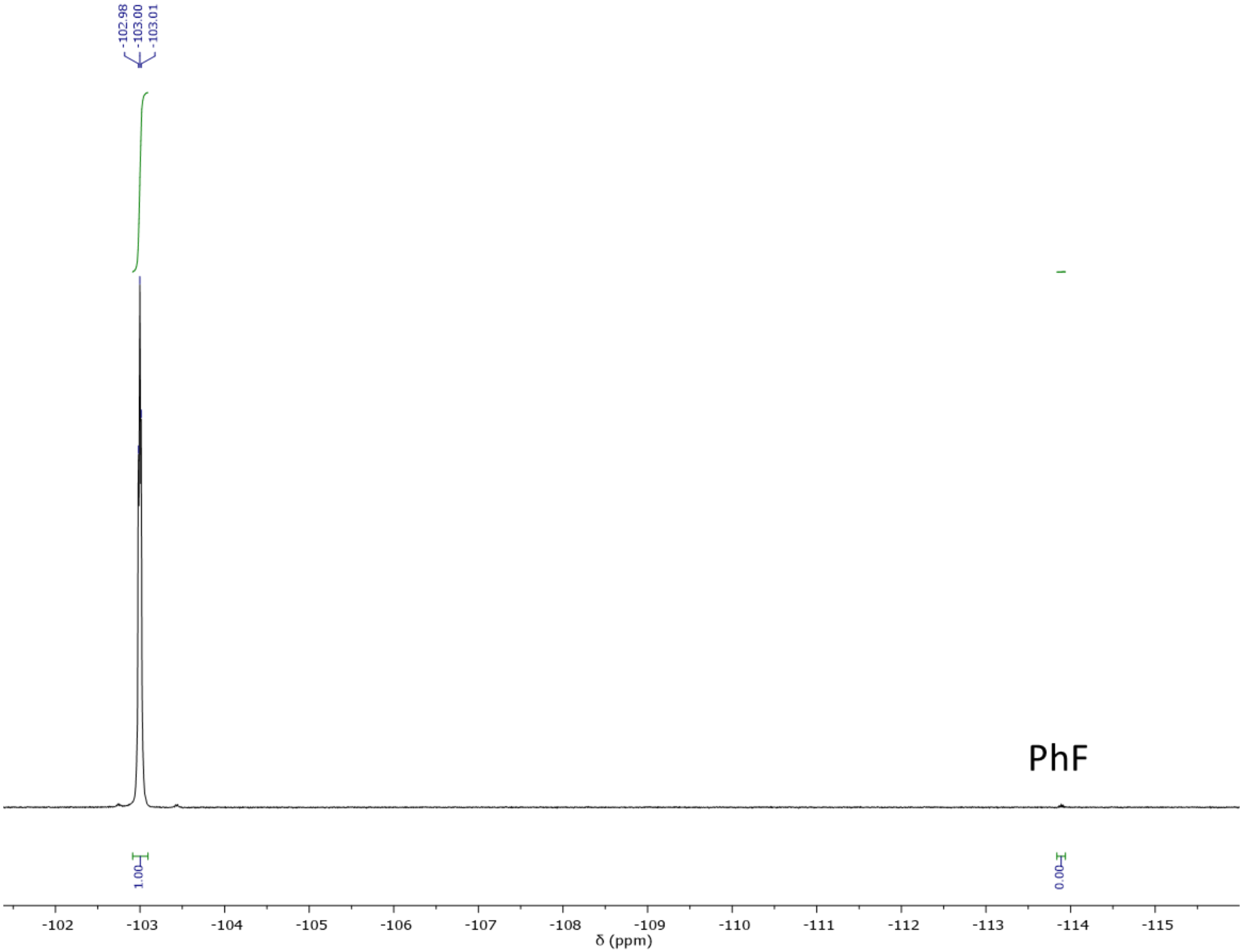

Figure S34. ^{19}F NMR (376 MHz, CD_2Cl_2) spectrum of **F_4Tr[Cl11]**. Residual PhF is also observed.

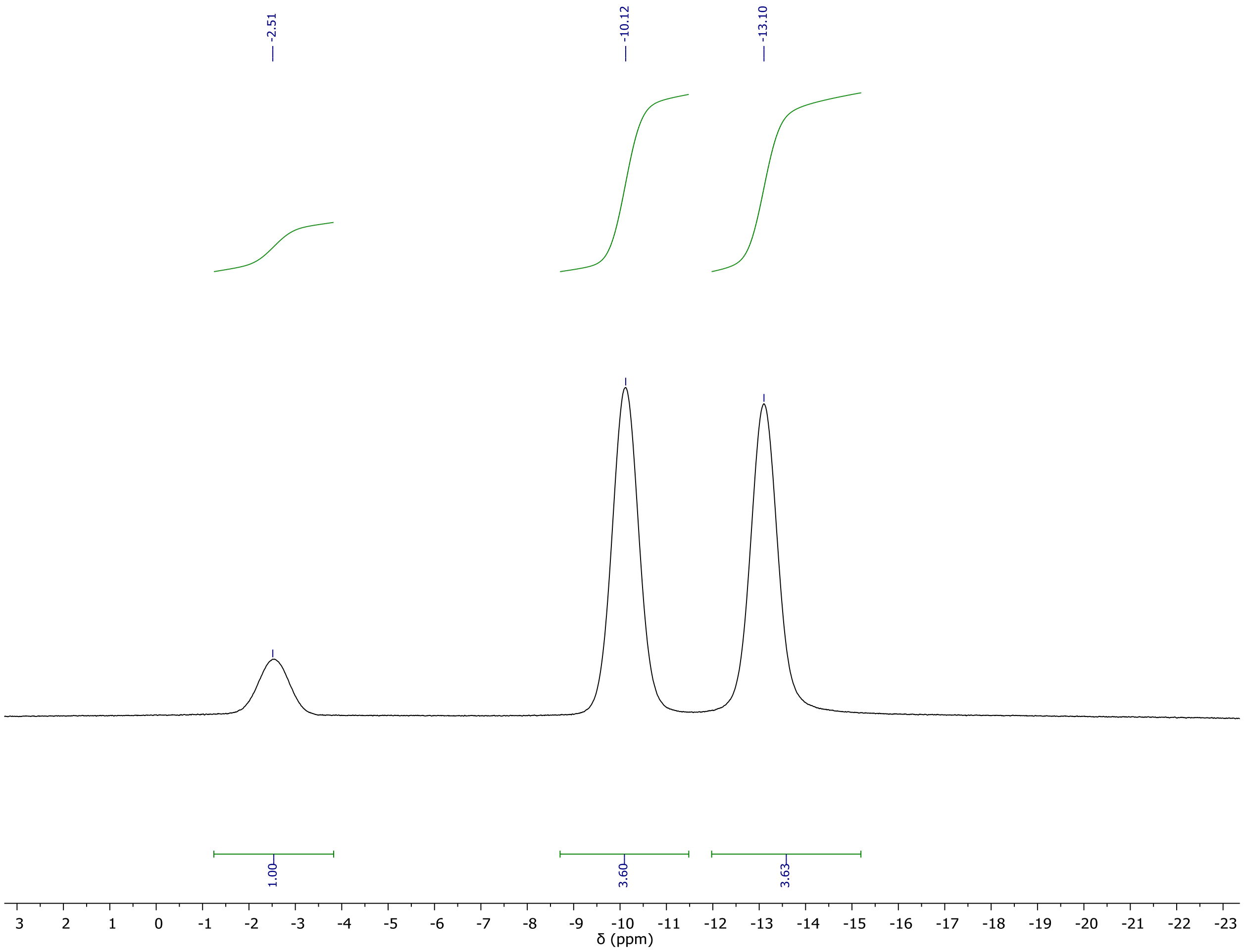

Figure S35. ^{11}B NMR (128 MHz, CD_2Cl_2) spectrum of **F_4Tr[Cl11]**.

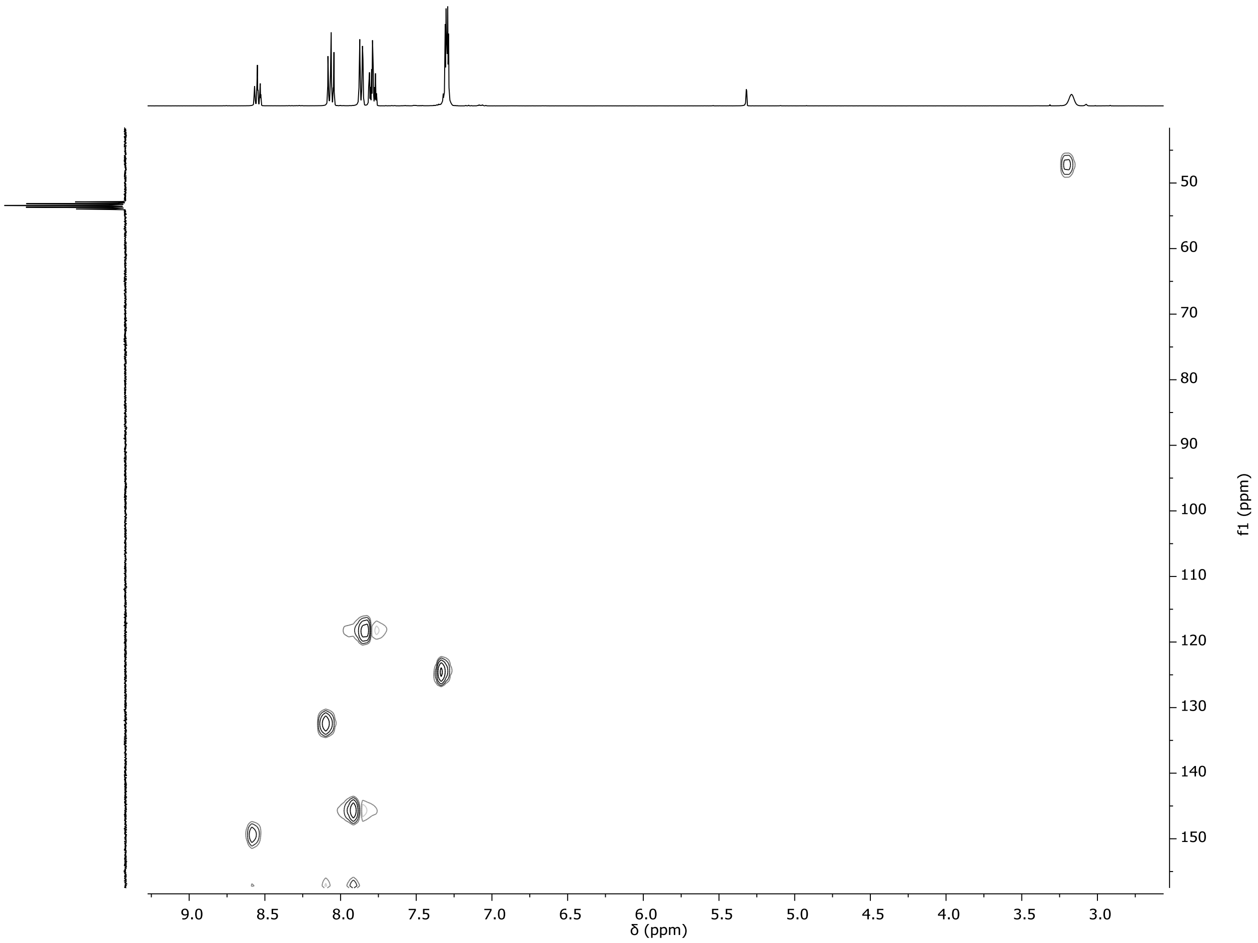

Figure S36. ^{13}C-^{1}H HSQC NMR (400 MHz, CD_2Cl_2) spectrum of **F_4Tr[Cl11]**.

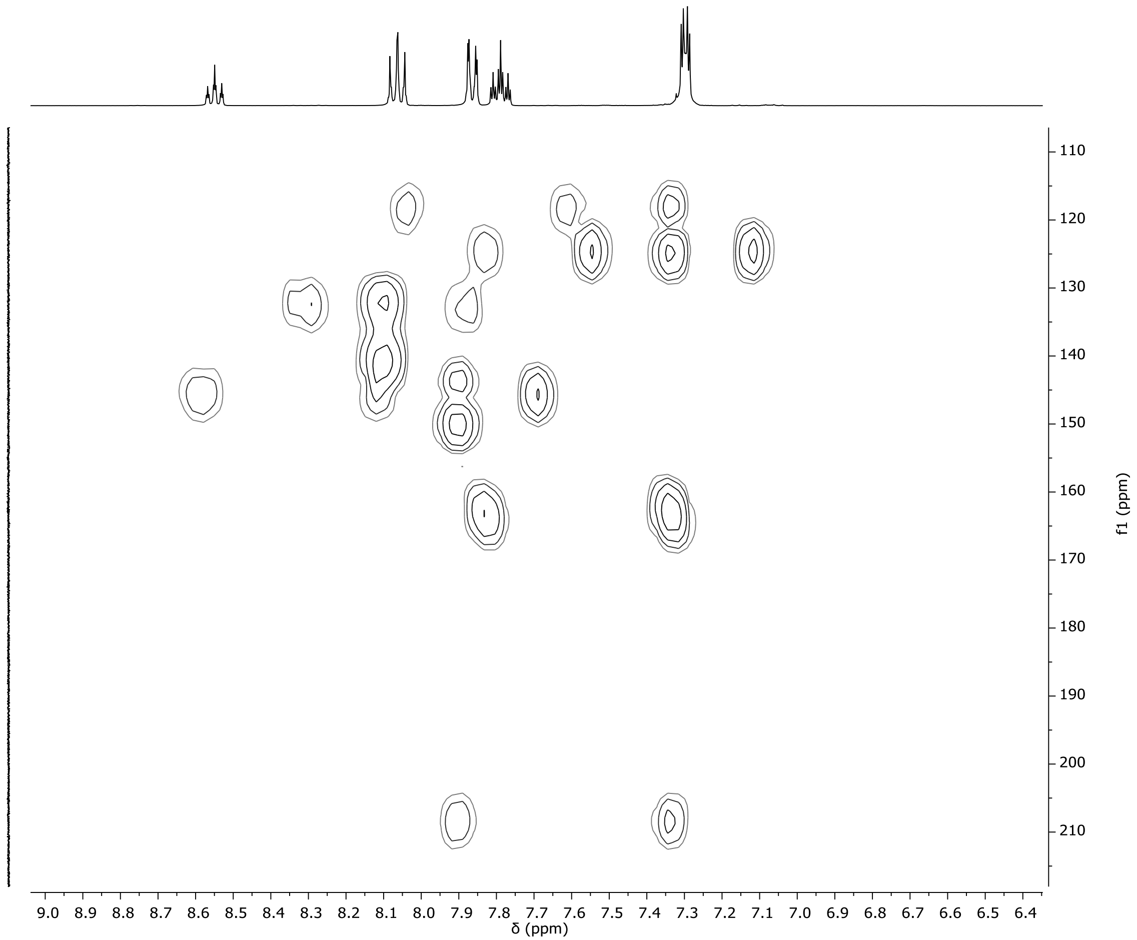

Figure S37. ^{13}C-^{1}H HMBC NMR (400 MHz, CD_2Cl_2) spectrum of **F_4Tr[Cl11]**.

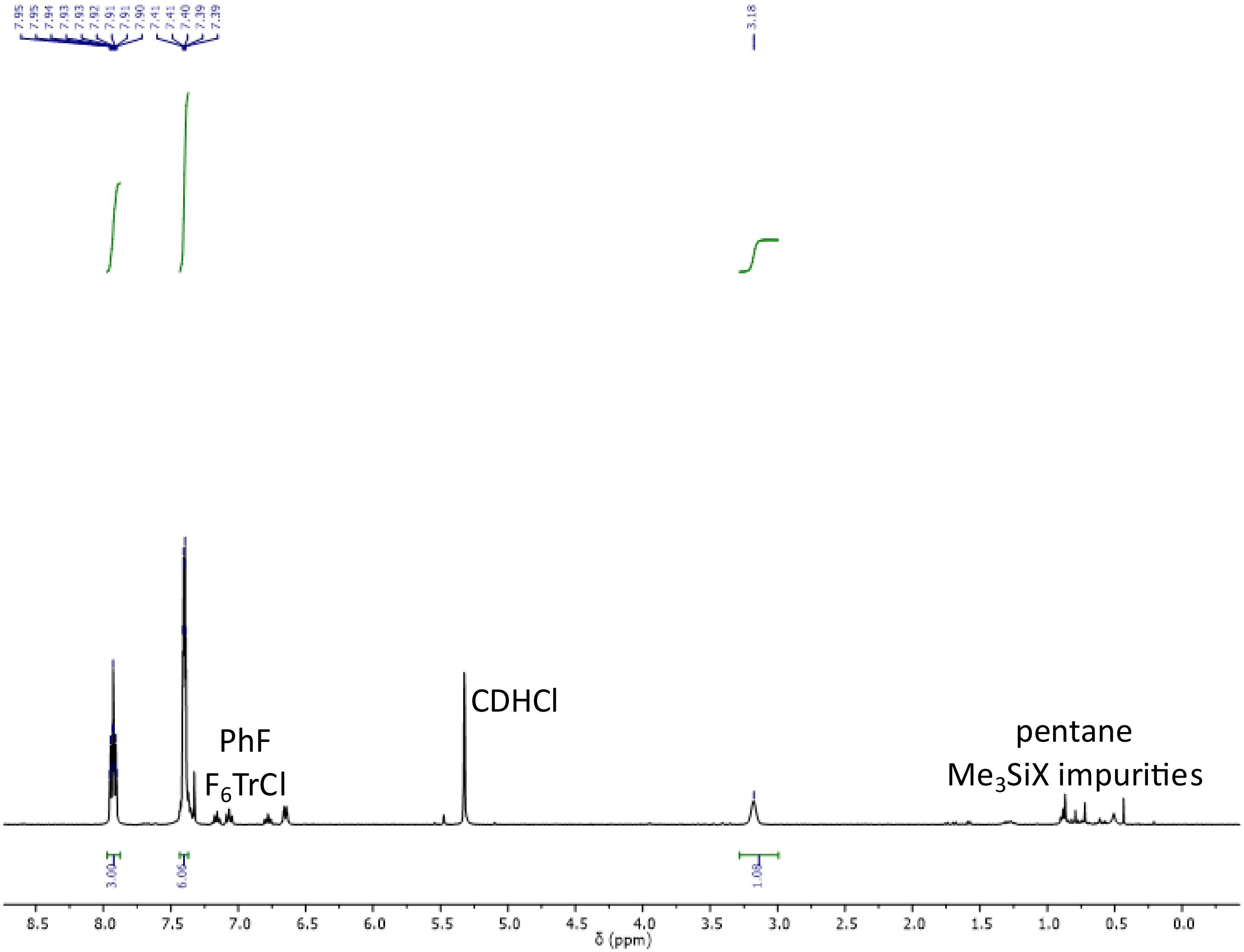

Figure S38. ^{1}H NMR (400 MHz, CD_2Cl_2) spectrum of **F_6Tr[Cl11]**. Residual pentane, Me_3SiX impurities, F_6TrCl, and PhF can be observed.

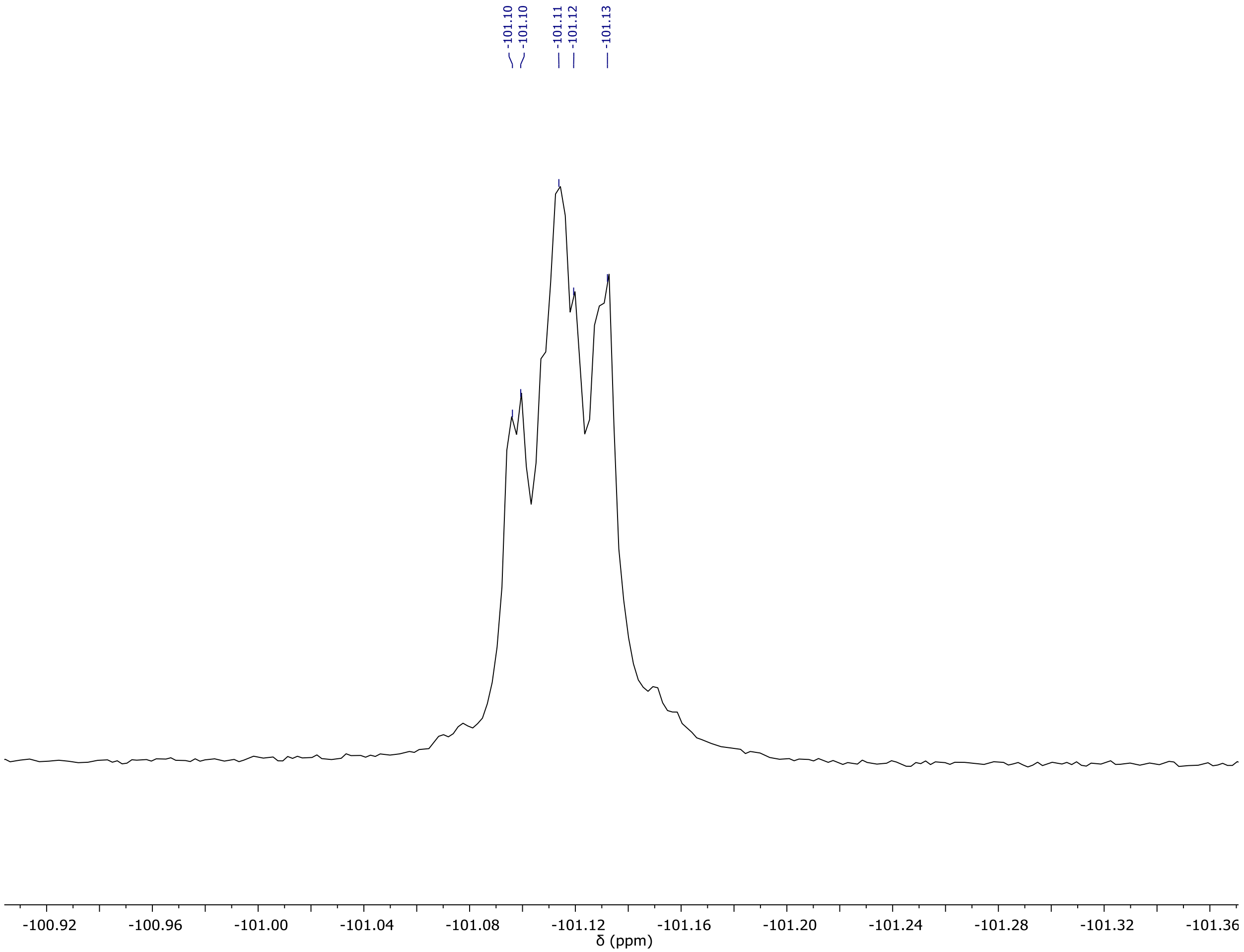

Figure S39. ^{19}F NMR (376 MHz, CD_2Cl_2) spectrum of **F_6Tr[Cl11]**.

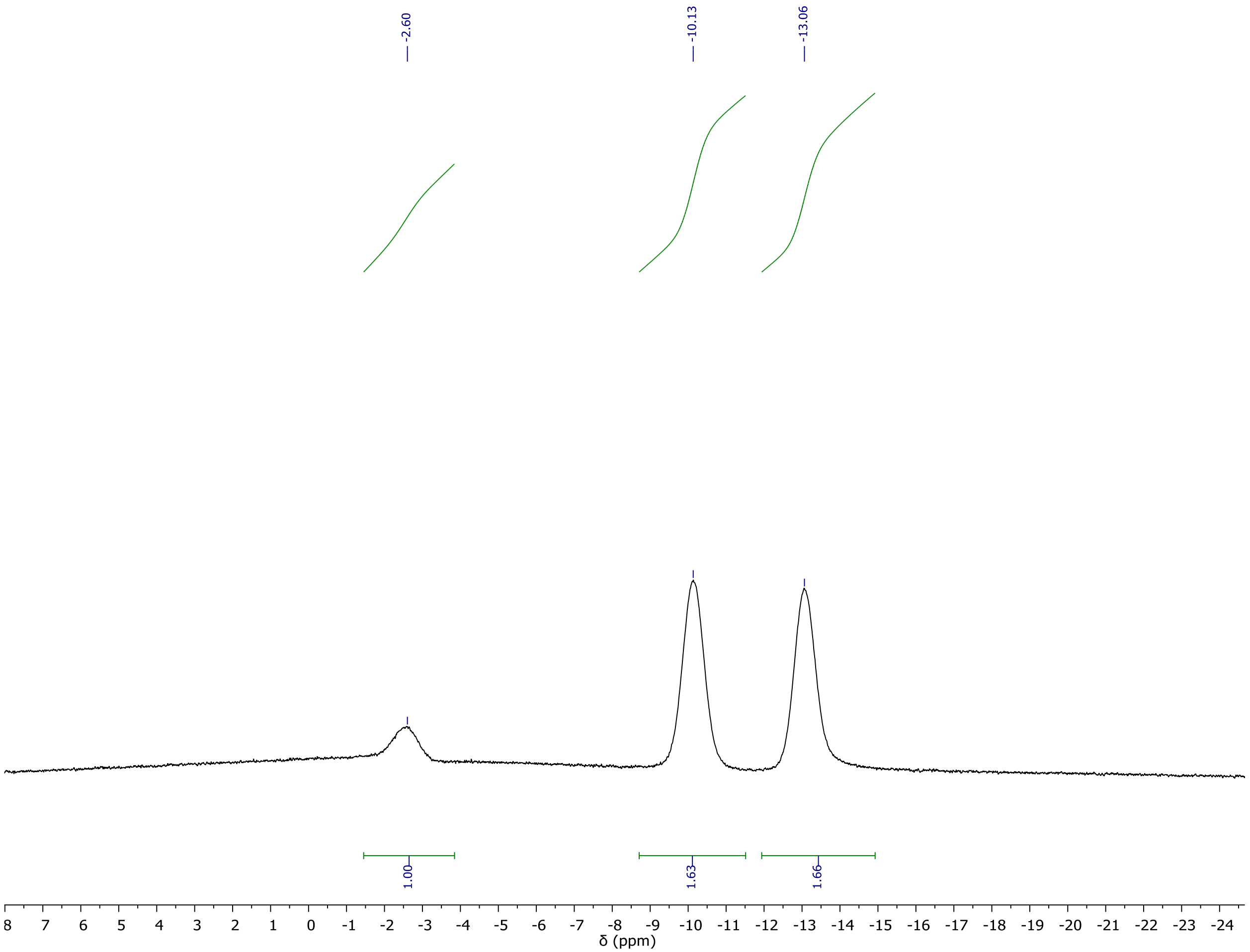

Figure S40. ^{11}B NMR (128 MHz, CD_2Cl_2) spectrum of **F_6Tr[Cl11]**.

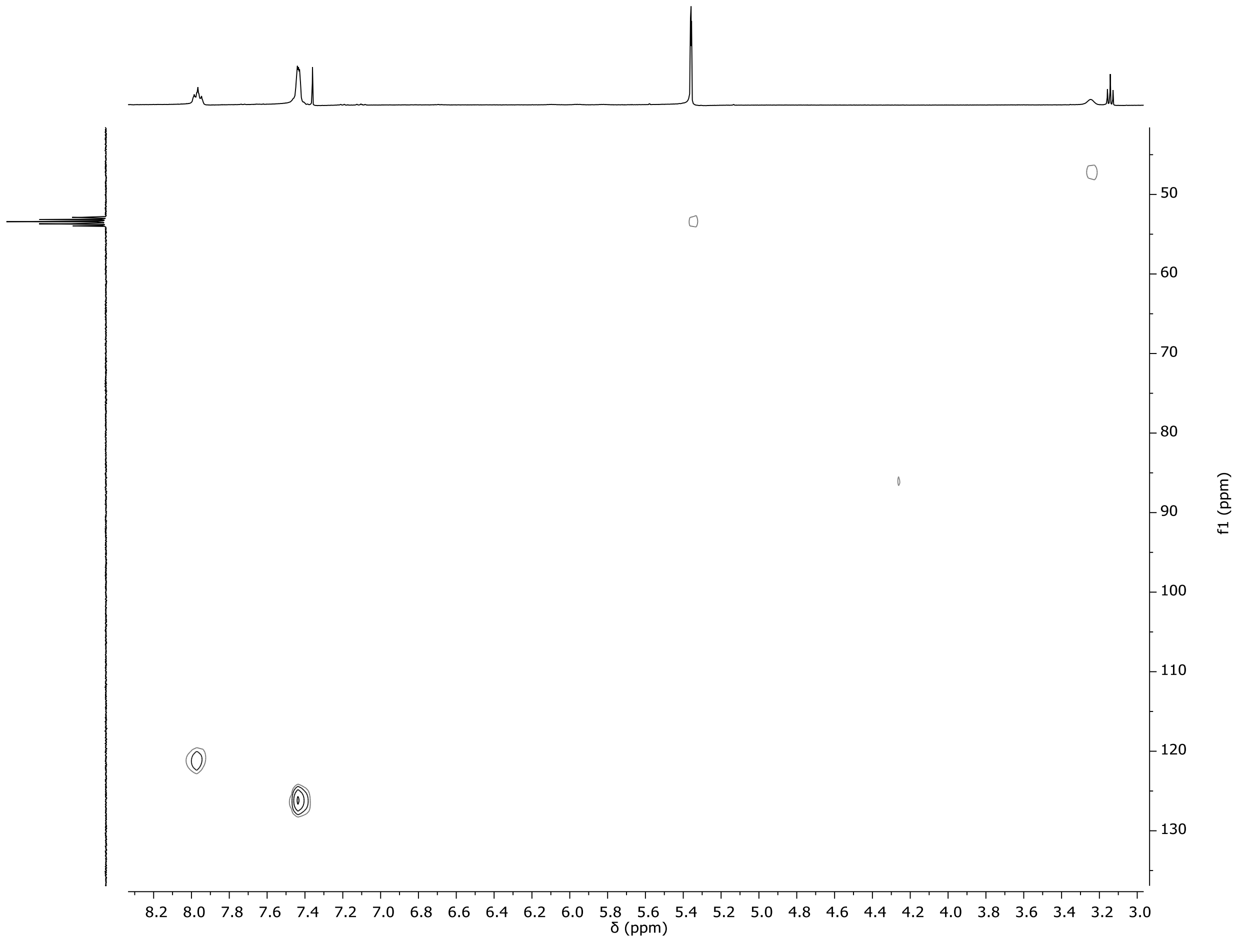

Figure S41. ^{13}C-^{1}H HSQC NMR (400 MHz, CD_2Cl_2) spectrum of **F_6Tr[Cl11]**.

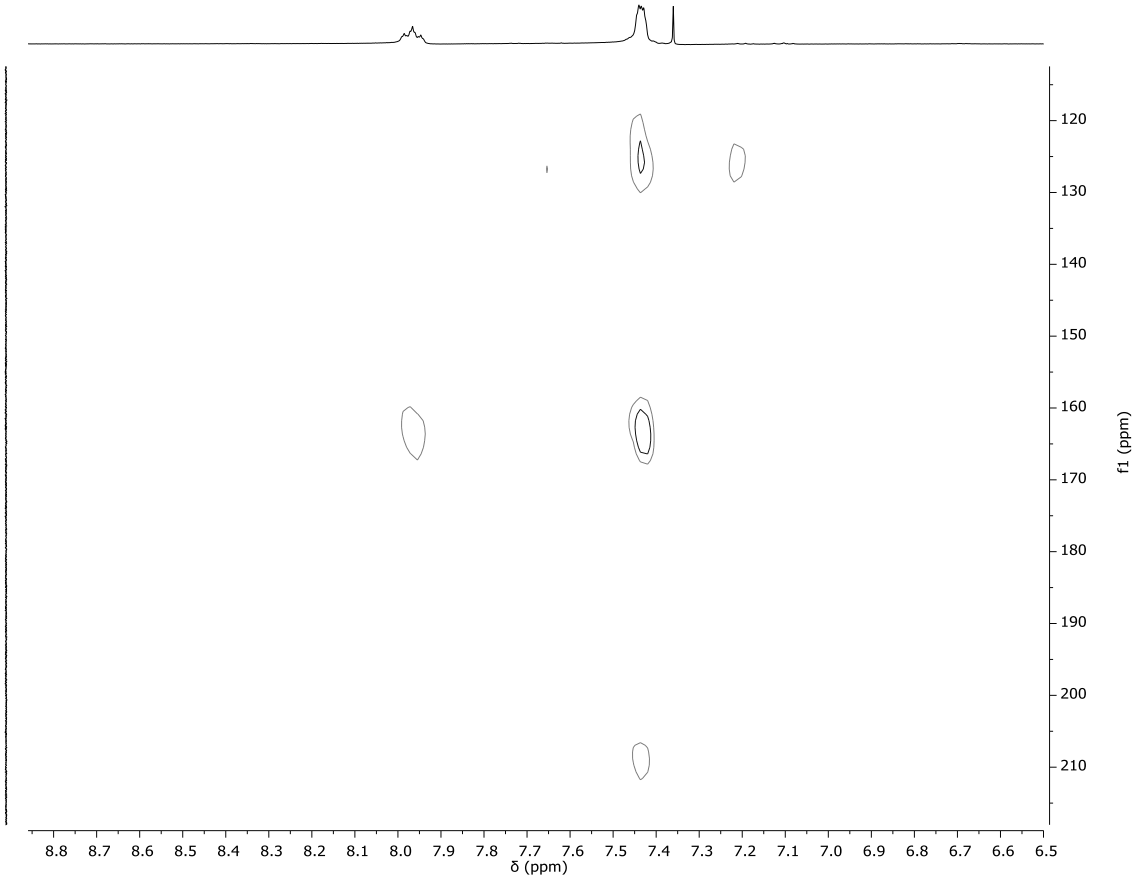

Figure S42. ^{13}C-^{1}H HMBC NMR (400 MHz, CD_2Cl_2) spectrum of **F_6Tr[Cl11]**.

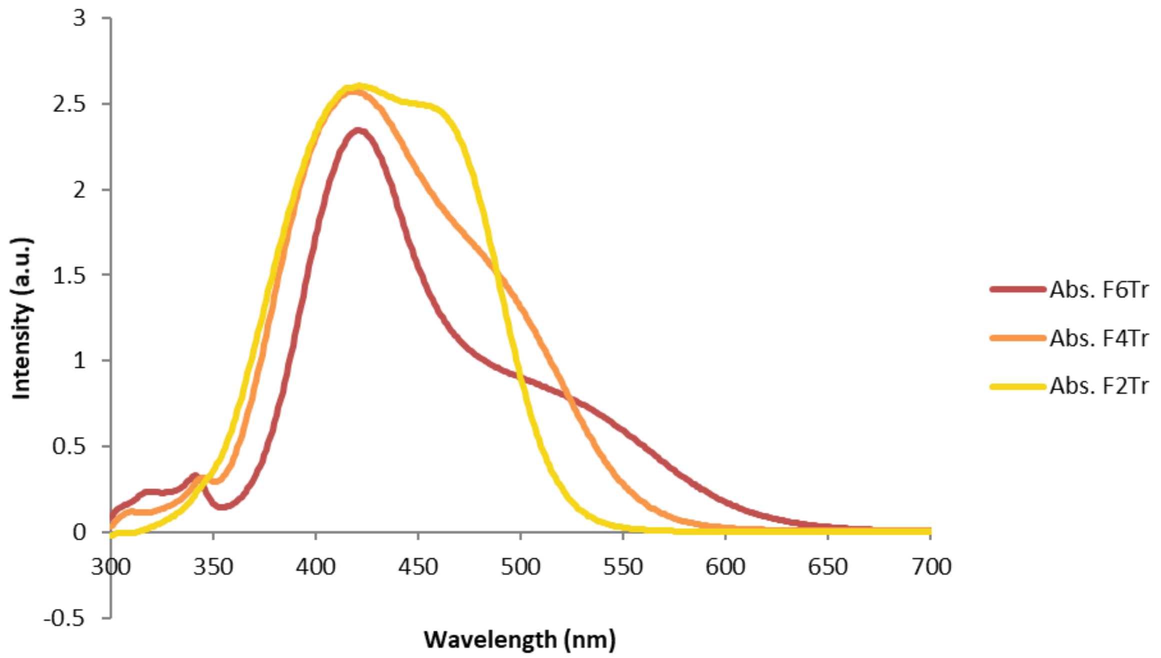

Figure S43. UV-Vis spectra of **F_2-, F_4-** and **F_6Tr[Cl_{11}]**. The spectra were obtained using a 0.11 mM samples in *o*-$C_6H_4Cl_2$.

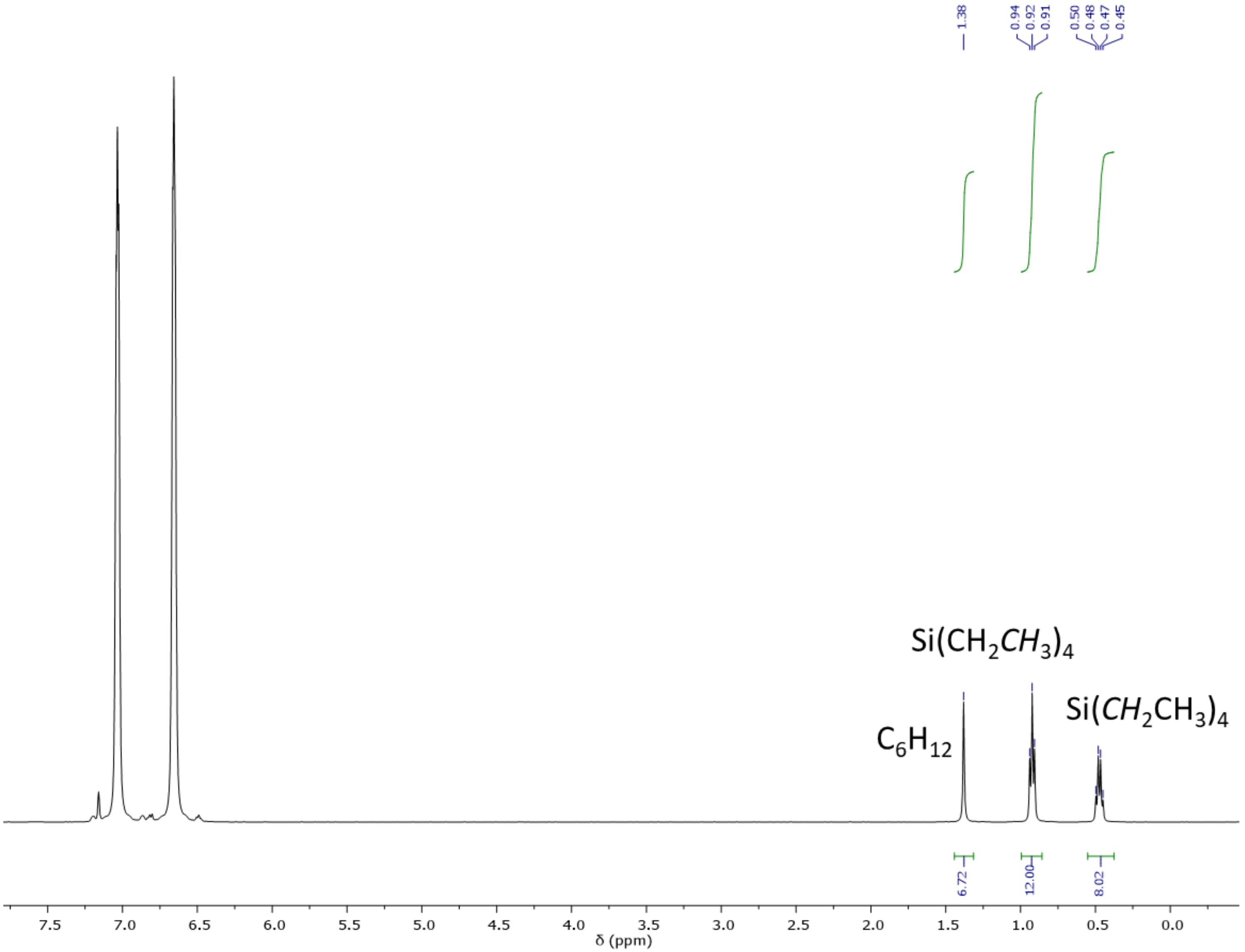

Figure S44. ^{1}H NMR (500 MHz, C_6D_6/*o*-$C_6H_4Cl_2$) spectrum of a stock solution containing $SiEt_4$, C_6H_{12}, and C_6F_6 (reagents).

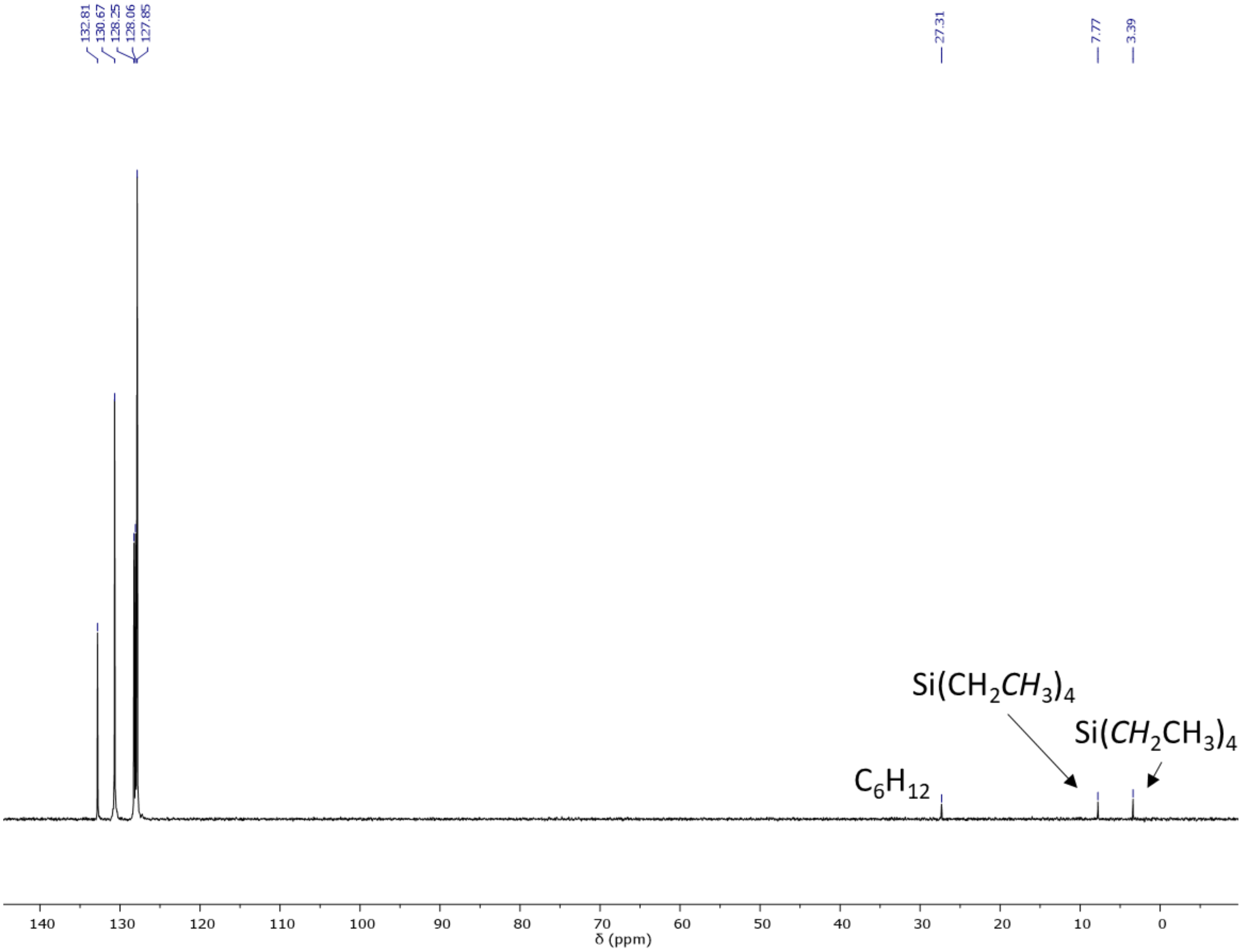

Figure S45. $^{13}C\{^1H\}$ NMR (126 MHz, C_6D_6/*o*-$C_6H_4Cl_2$) spectrum of a stock solution containing $SiEt_4$, C_6H_{12}, and C_6F_6. The concentration of C_6F_6 was not sufficiently high for ^{13}C NMR spectroscopy (reagents).

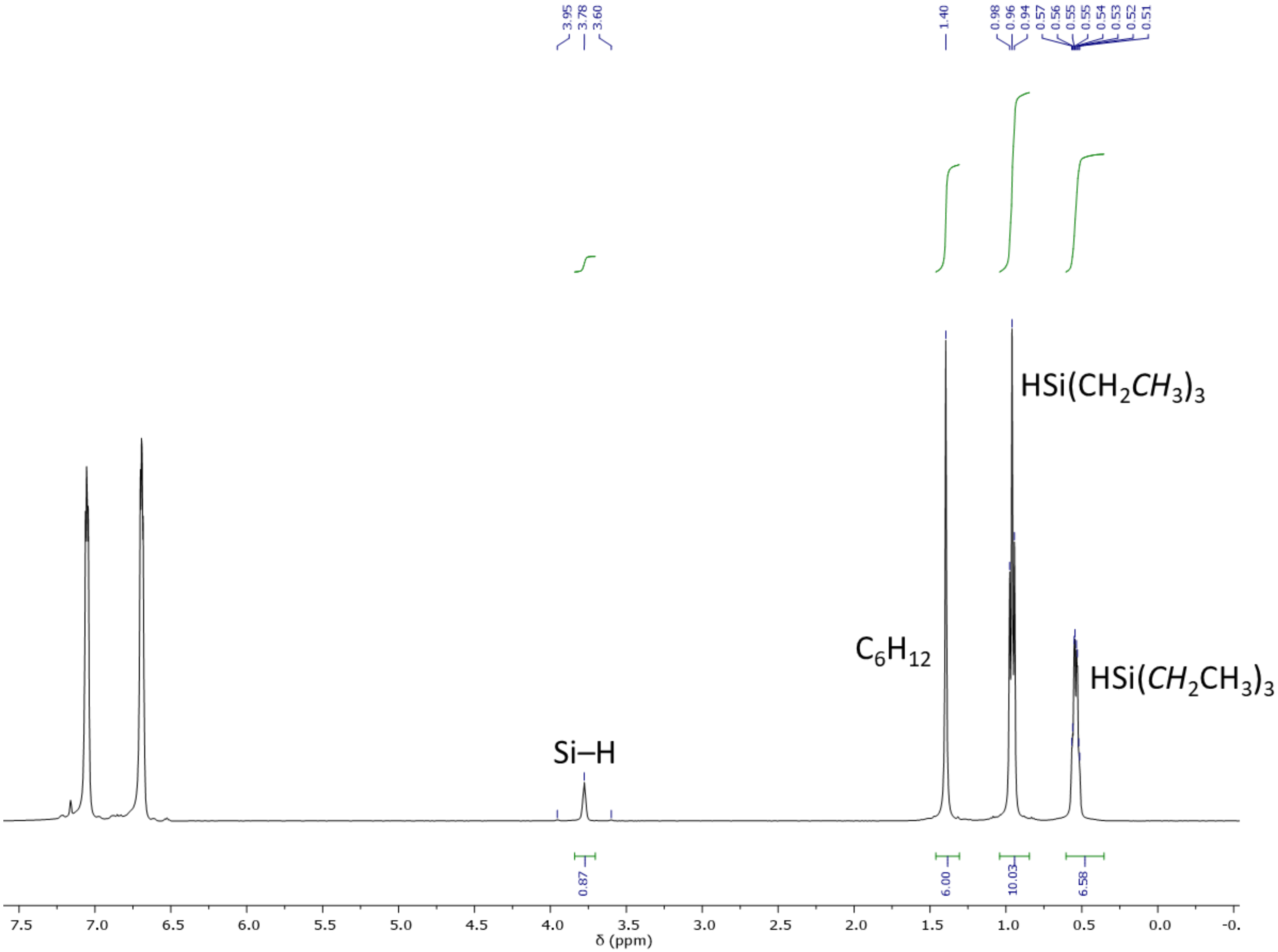

Figure S46. ^{1}H NMR (500 MHz, C_6D_6/*o*-$C_6H_4Cl_2$) spectrum of a stock solution containing $HSiEt_3$, C_6H_{12}, and C_6F_6 (reagents).

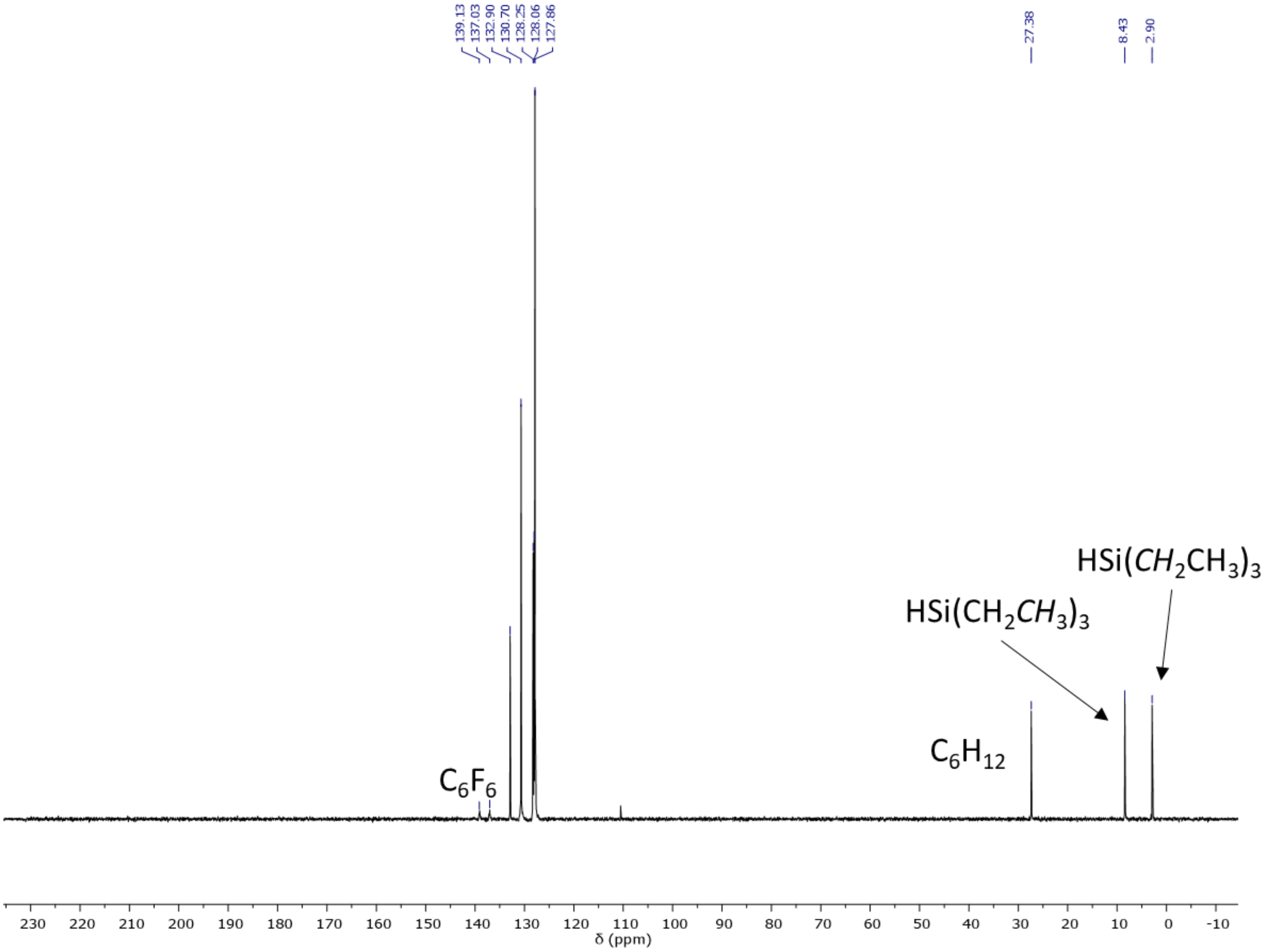

Figure S47. $^{13}C\{^{1}H\}$ NMR (126 MHz, C_6D_6/*o*-$C_6H_4Cl_2$) spectrum of a stock solution containing $HSiEt_3$, C_6H_{12}, and C_6F_6. An artifact of the instrument can be observed at 110 ppm (reagents).

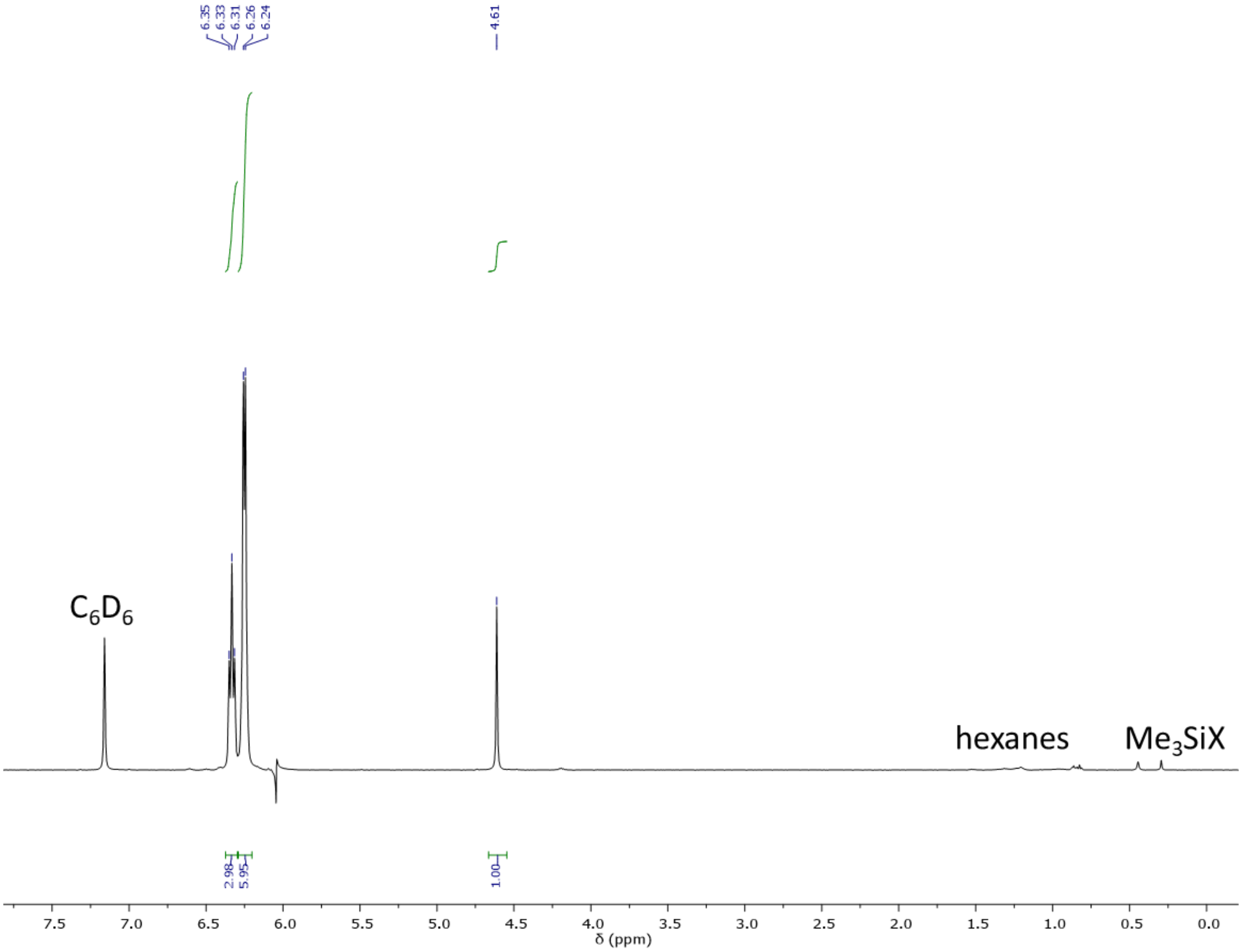

Figure S48. ^{1}H NMR (500 MHz, C_6D_6) spectrum of **F_6TrH**. Residual hexanes, Me_3SiX byproducts, and an artifact of the instrument are also observed (**R1**).

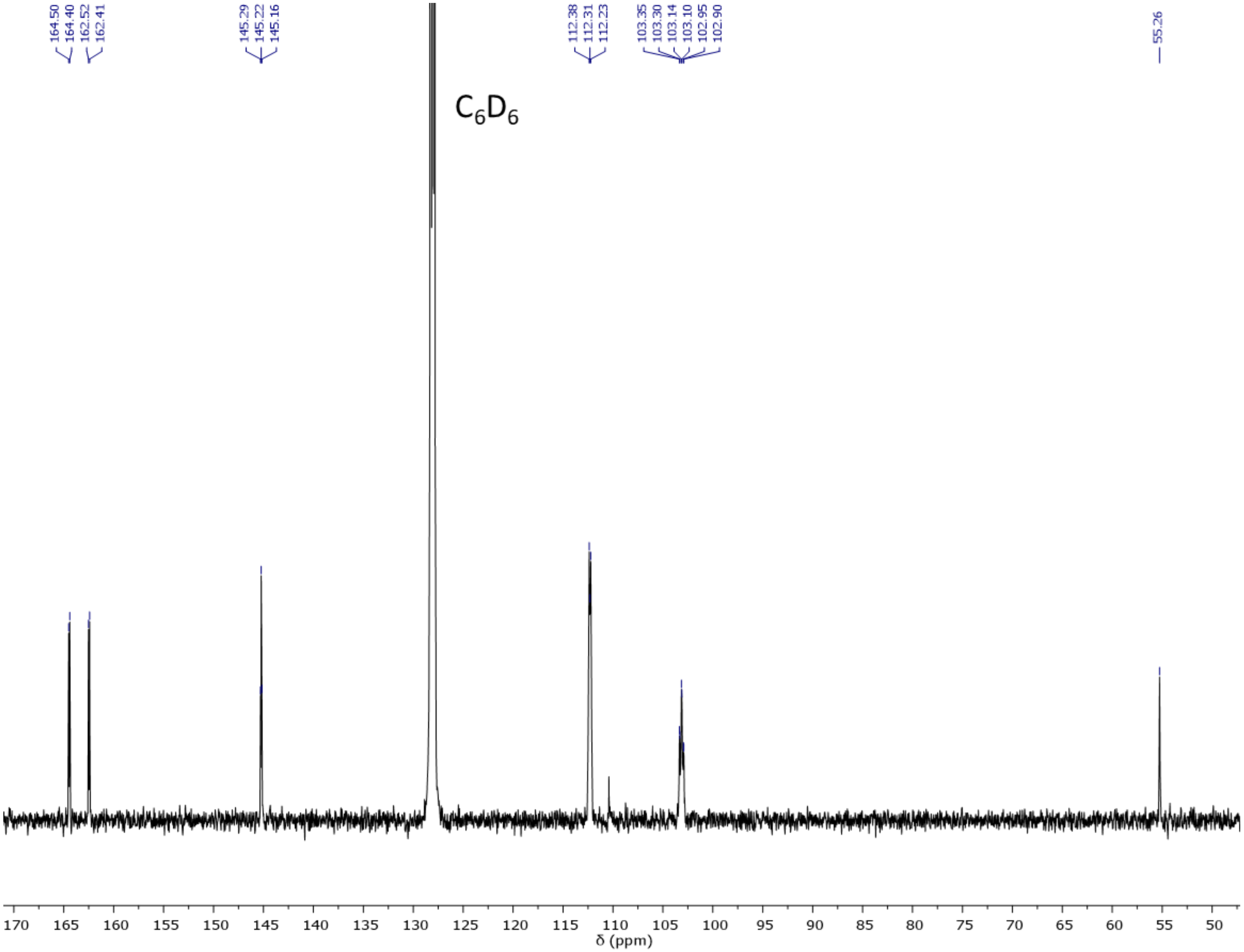

Figure S49. $^{13}C\{^1H\}$ (126 MHz, C_6D_6) spectrum of **F_6TrH** (**R1**).

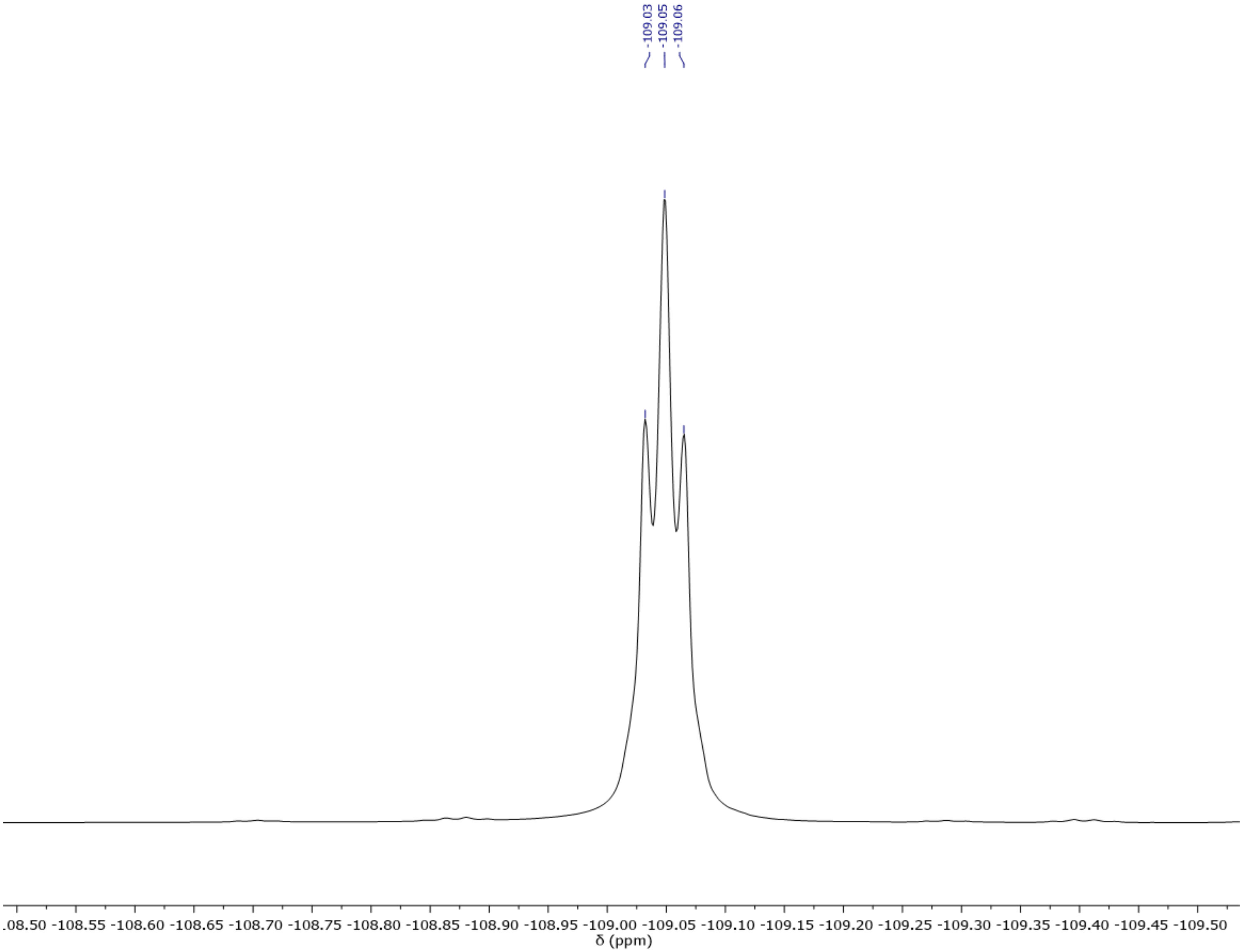

Figure S50. ^{19}F NMR (470 MHz, C_6D_6) spectrum of $\mathbf{F_6TrH}$ (**R1**).

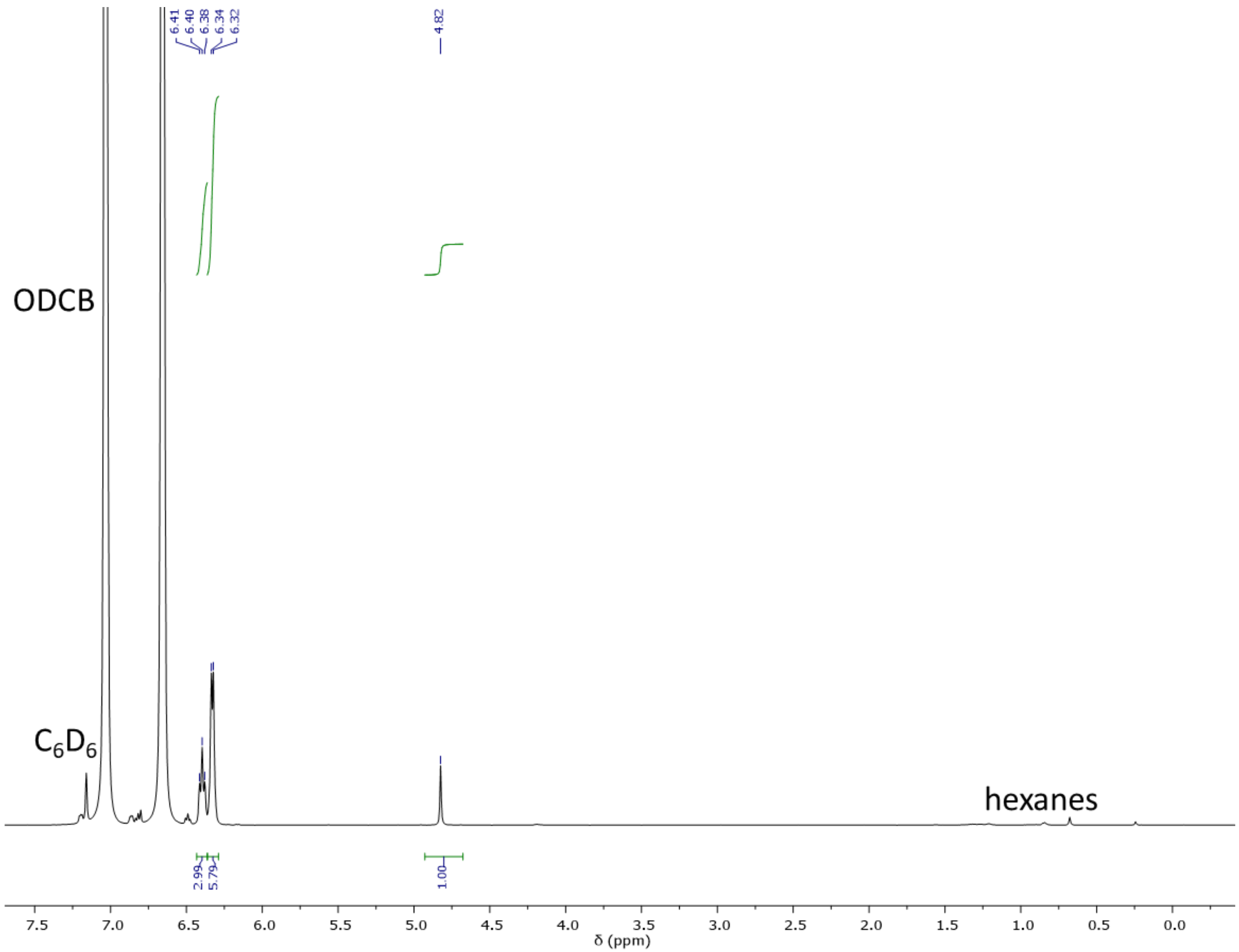

Figure S51. ^{1}H NMR (500 MHz, C_6D_6/*o*-$C_6H_4Cl_2$) spectrum of **F_6TrH**. Residual hexanes can also be observed (**R1**).

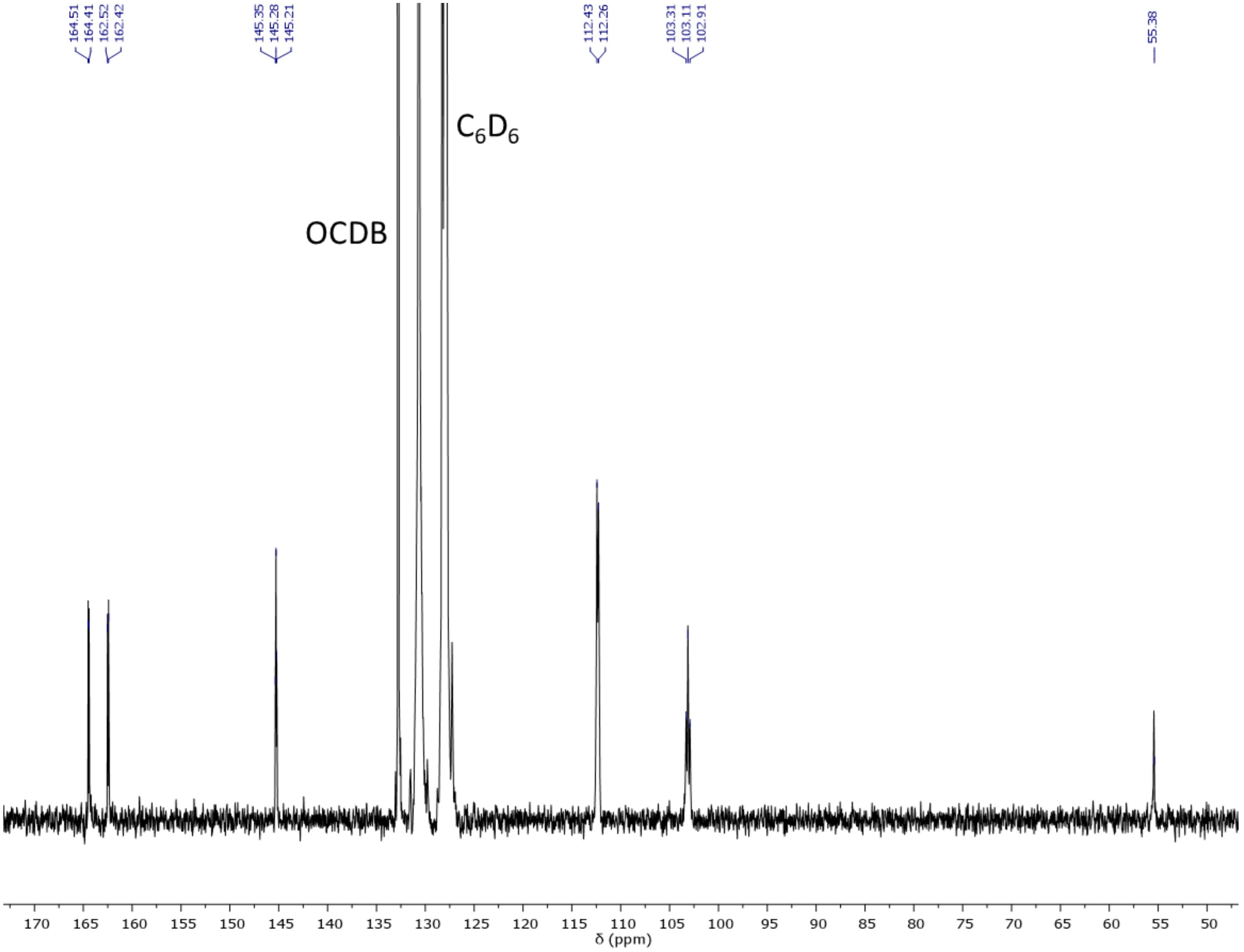

Figure S52. $^{13}C\{^1H\}$ NMR (126 MHz, C_6D_6/*o*-$C_6H_4Cl_2$) spectrum of **F_6TrH** (**R1**).

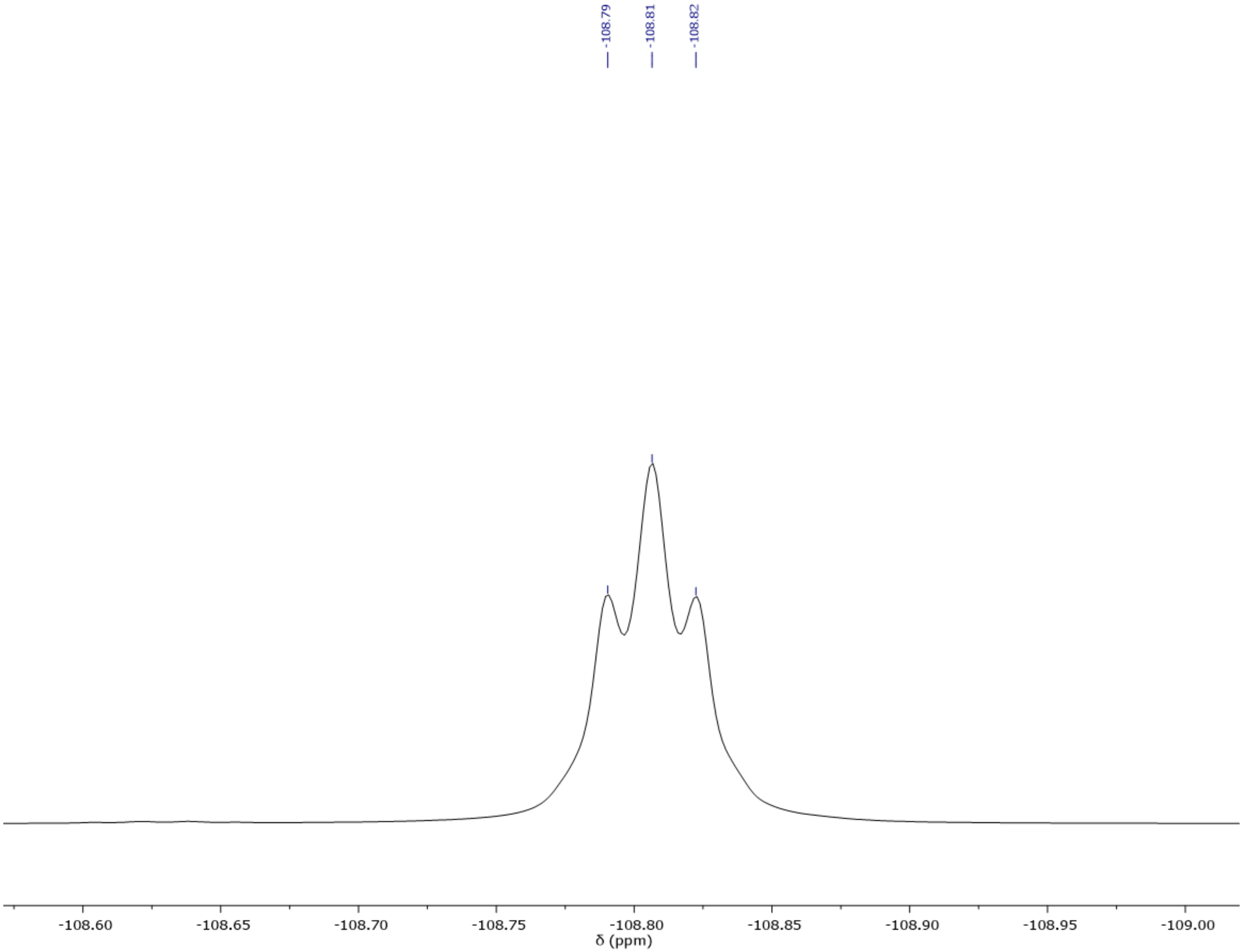

Figure S53. ^{19}F NMR (470 MHz, C_6D_6/*o*-$C_6H_4Cl_2$) spectrum of **F_6TrH (R1)**.

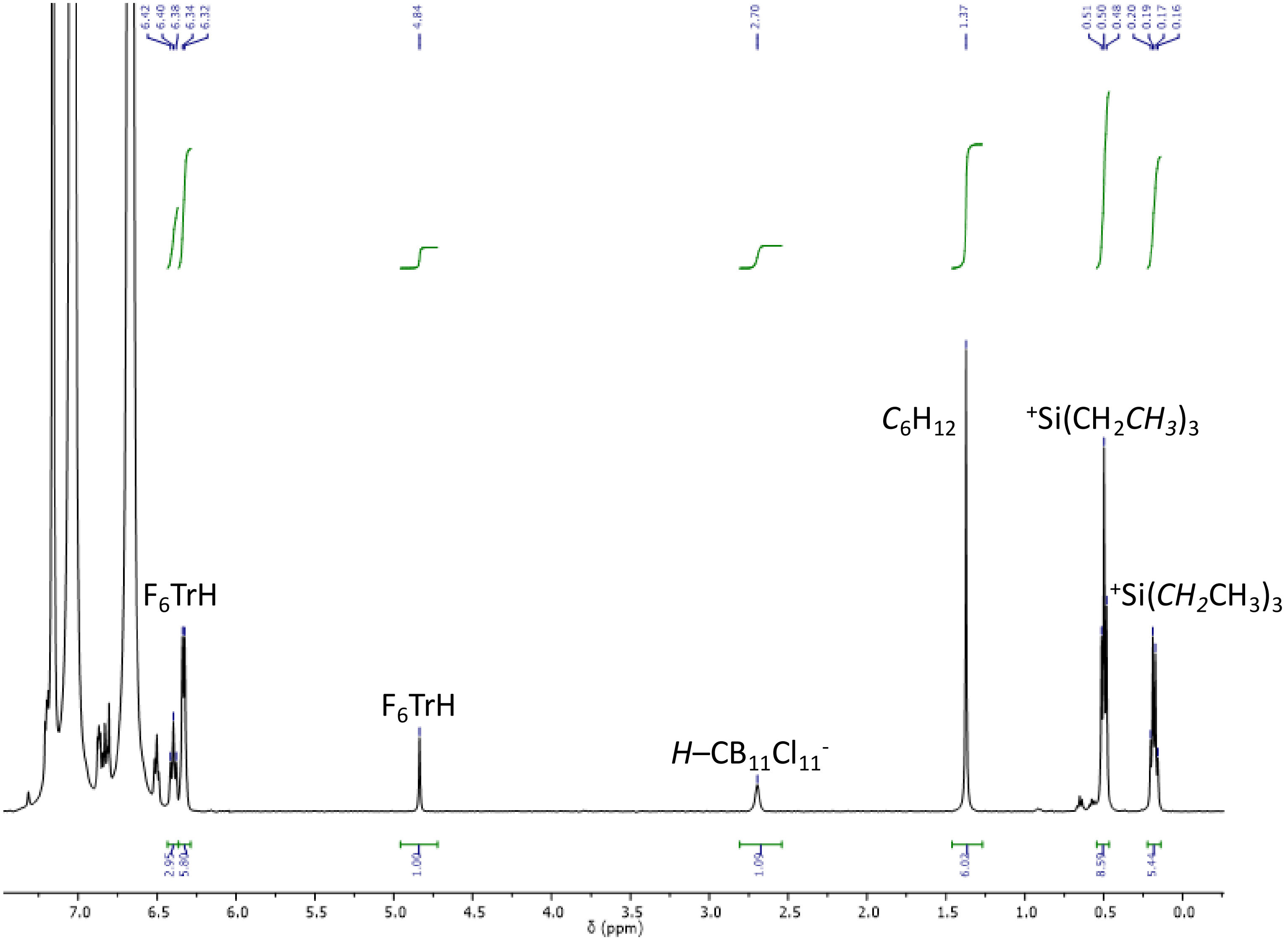

Figure S54. ^{1}H NMR (500 MHz, C_6D_6/*o*-$C_6H_4Cl_2$) spectrum taken 30 min after reaction of **F_6Tr[Cl11]** with 0.75 eq $HSiEt_3$ (**R2**).

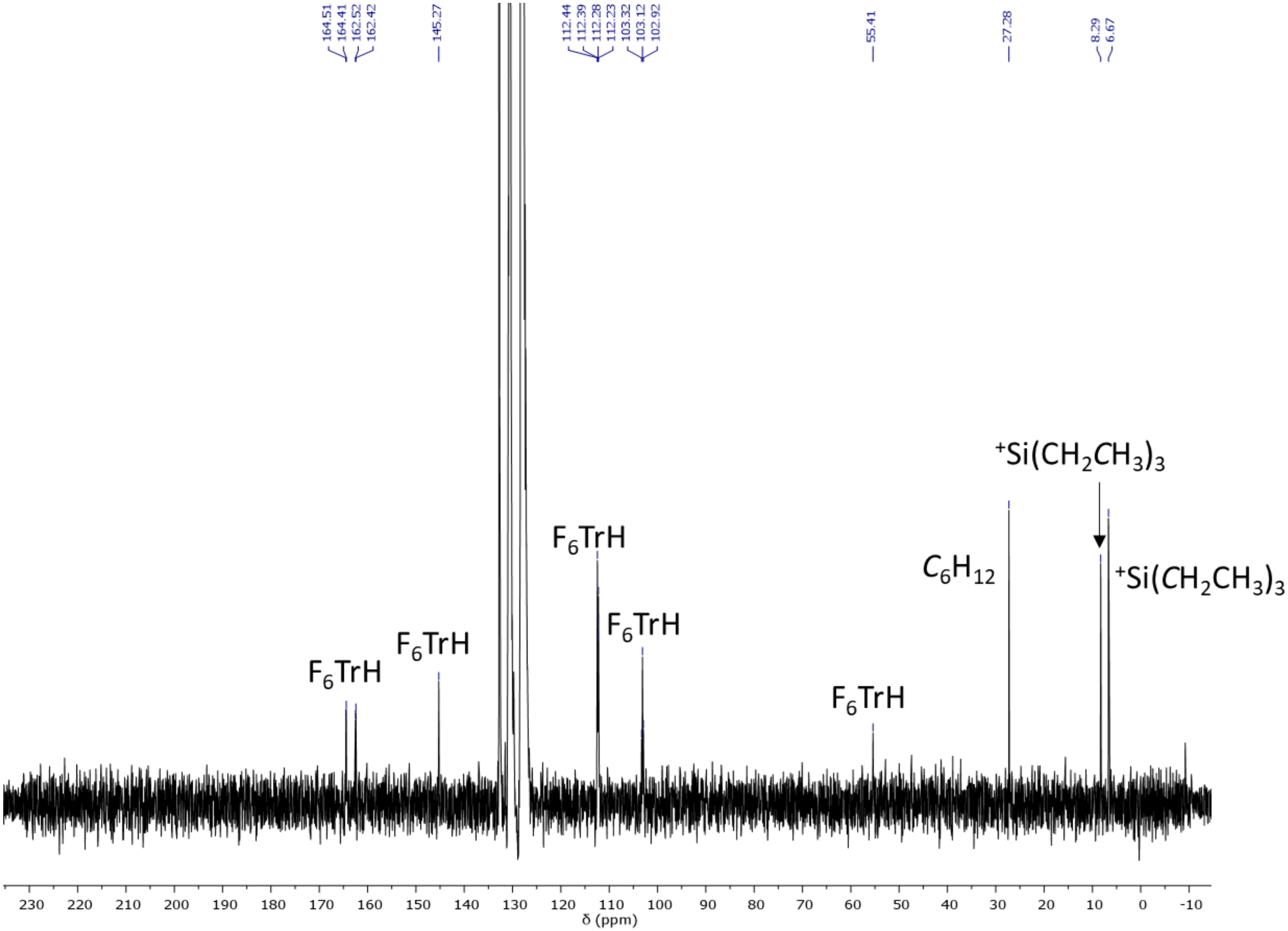

Figure S55. Full $^{13}C\{^{1}H\}$ NMR (126 MHz, C_6D_6/*o*-$C_6H_4Cl_2$) spectrum taken 30 min after reaction of **$F_6Tr[Cl_{11}]$** with 0.75 eq $HSiEt_3$ (**R2R4**).

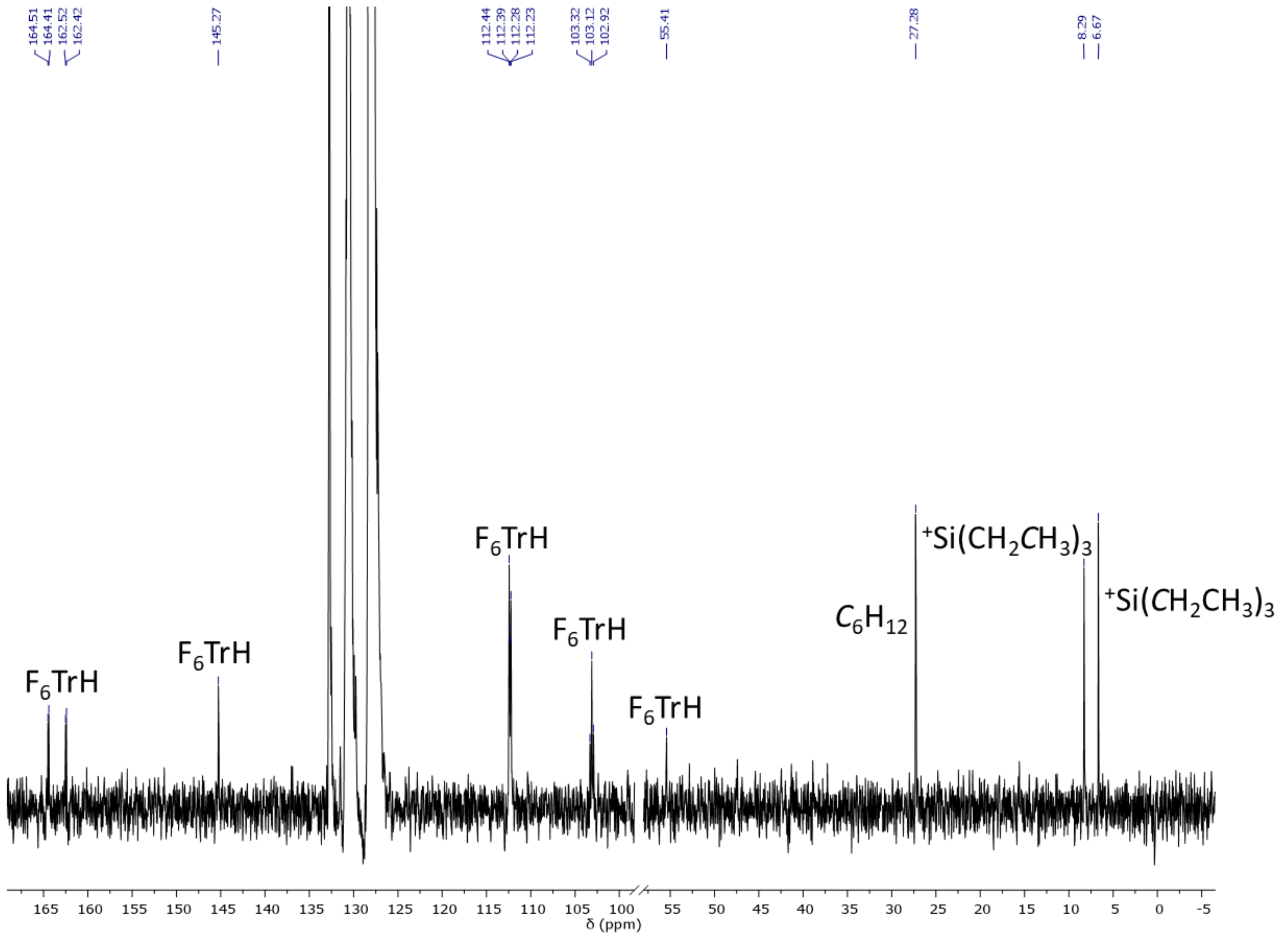

Figure S56. Truncated $^{13}C\{^{1}H\}$ NMR (126 MHz, C_6D_6/*o*-$C_6H_4Cl_2$) spectrum taken 30 min after reaction of **F_6Tr[Cl11]** with 0.75 eq $HSiEt_3$ (**R2**).

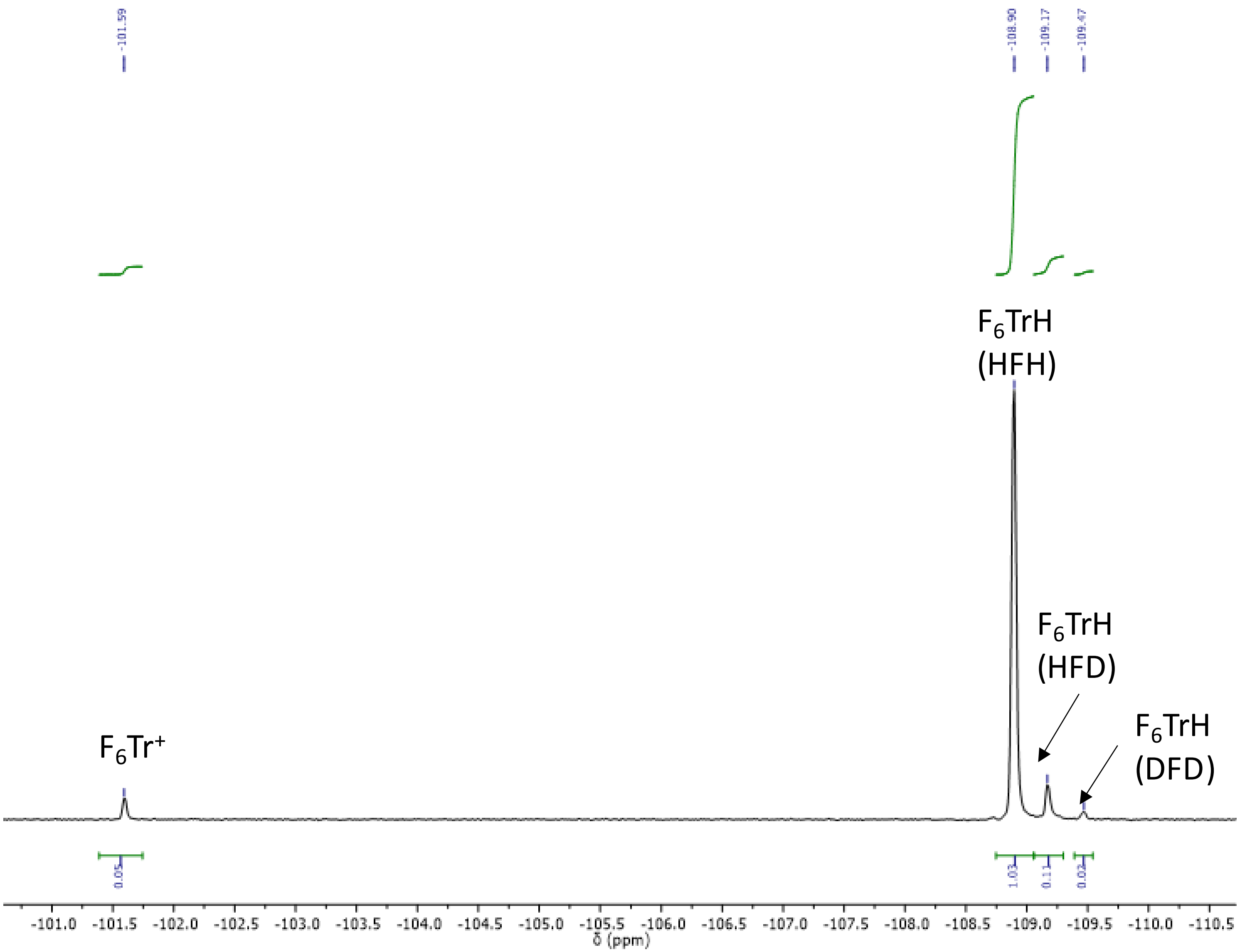

Figure S57. ^{19}F NMR (470 MHz, C_6D_6/*o*-$C_6H_4Cl_2$) spectrum taken 30 min after reaction of **F_6Tr[Cl11]** with 0.9 eq $HSiEt_3$. Three resonances are observed for the three isotopic environments of the F-atoms in **F_6TrH**: HFH, HFD, and DFD (**R4**).

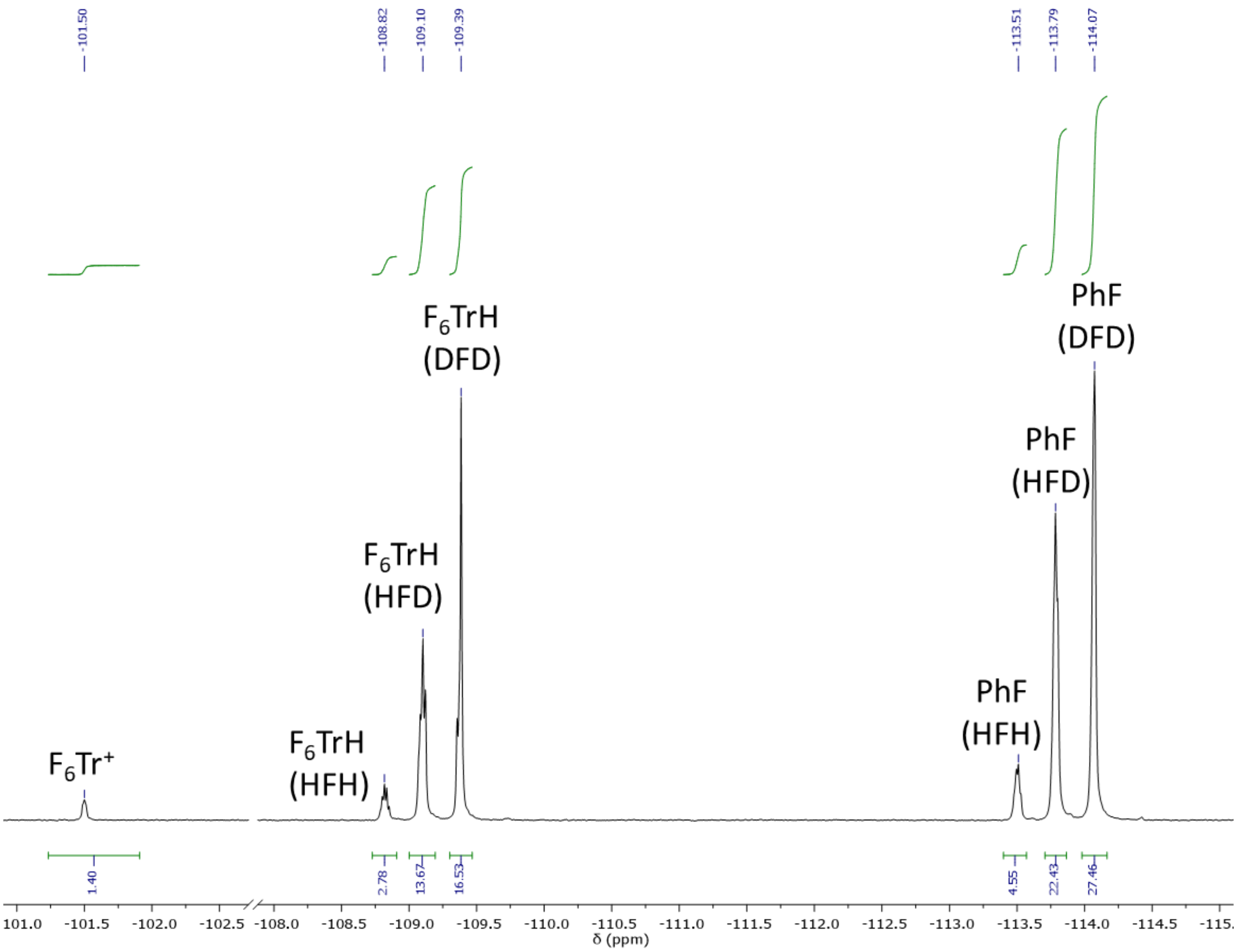

Figure S58. ^{19}F NMR (470 MHz, C_6D_6/*o*-$C_6H_4Cl_2$) spectrum taken 15 min after the addition of PhF to RX. Three resonances are observed for the three isotopic environments of the F-atoms in **F_6TrH** and PhF: HFH, HFD, and DFD (**R4**).

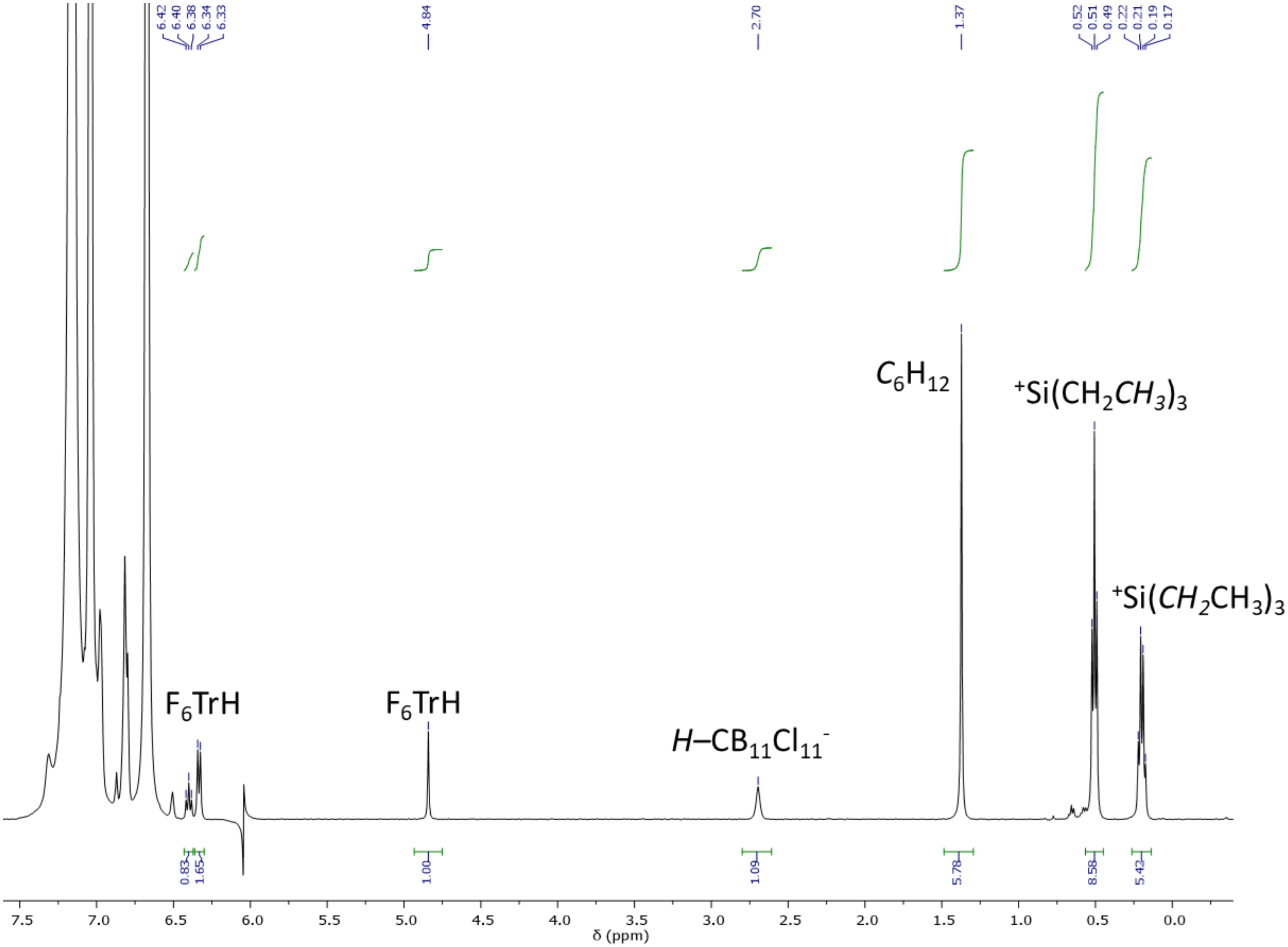

Figure S59. ^1H NMR (500 MHz, C_6D_6/*o*-$C_6H_4Cl_2$) spectrum taken 15 min after reaction the addition of PhF to **R4**.

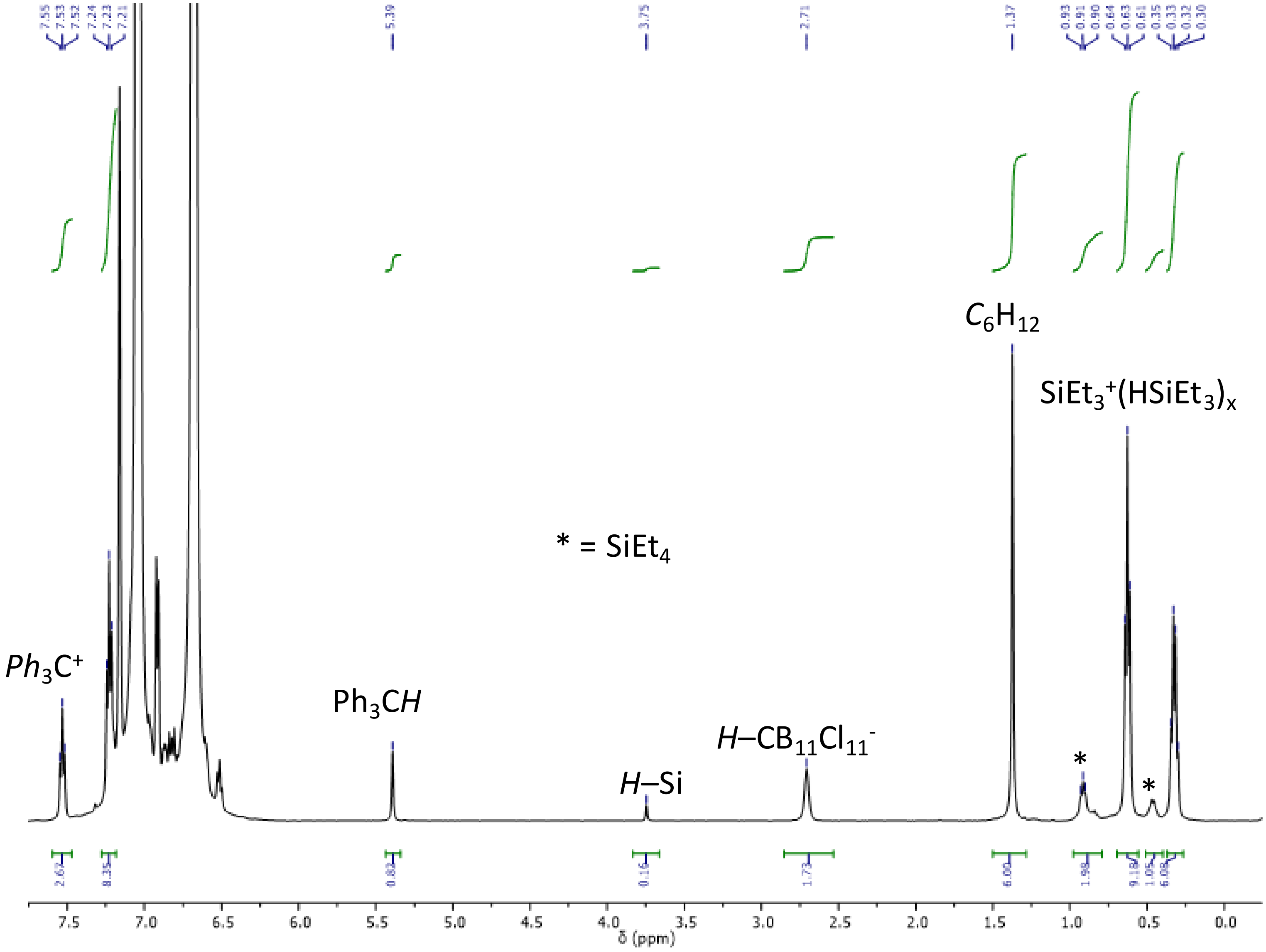

Figure S60. ^{1}H NMR (500 MHz, C_6D_6/*o*-$C_6H_4Cl_2$) spectrum taken 30 min after reaction of **Tr[Cl11]** with 0.9 eq $HSiEt_3$ (**R3**).

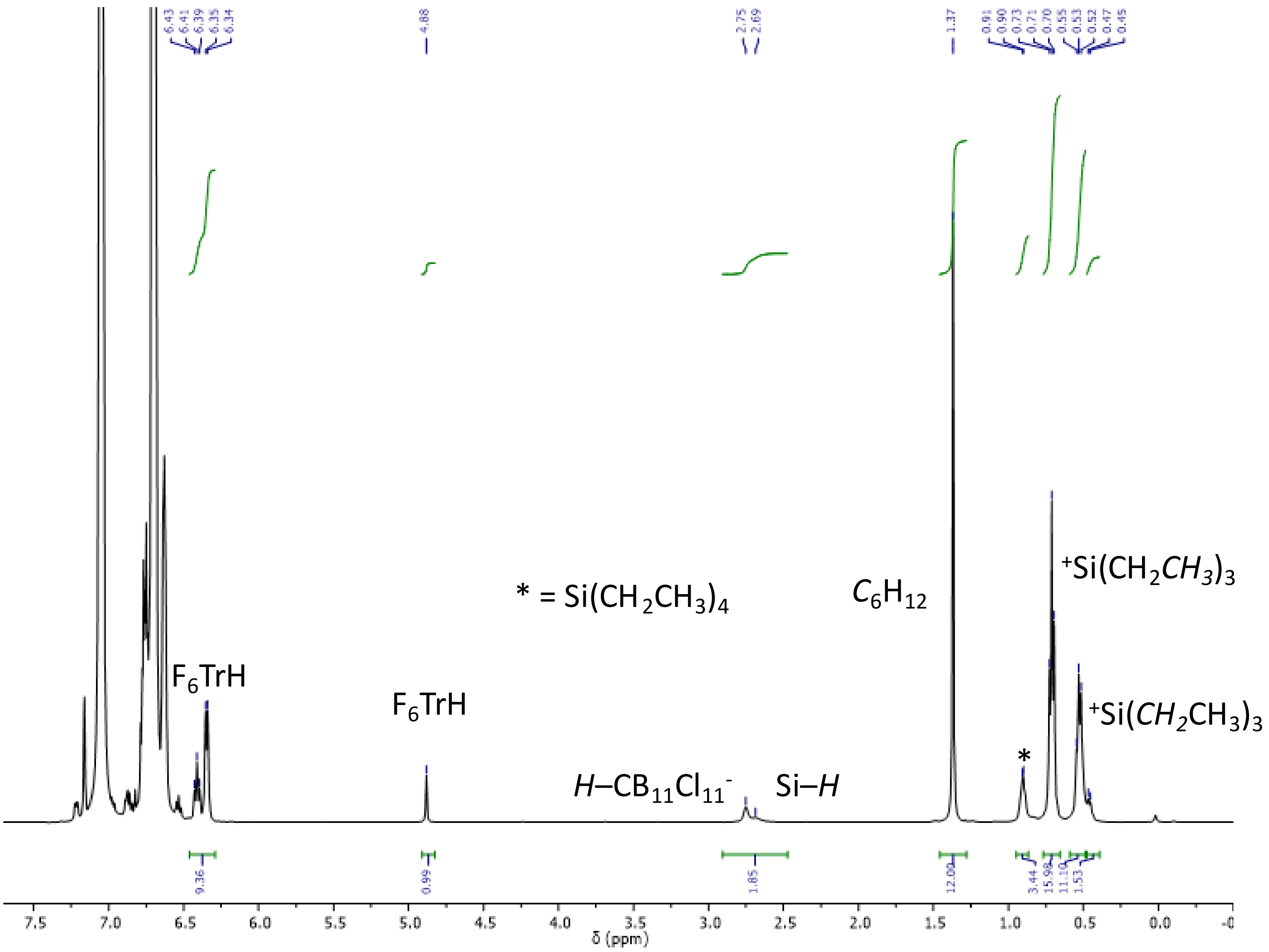

Figure S61. ^{1}H NMR (500 MHz, C_6D_6/*o*-$C_6H_4Cl_2$) spectrum of **F_6Tr[Cl11]** and 2 eq of $HSiEt_3$ (**R4**).

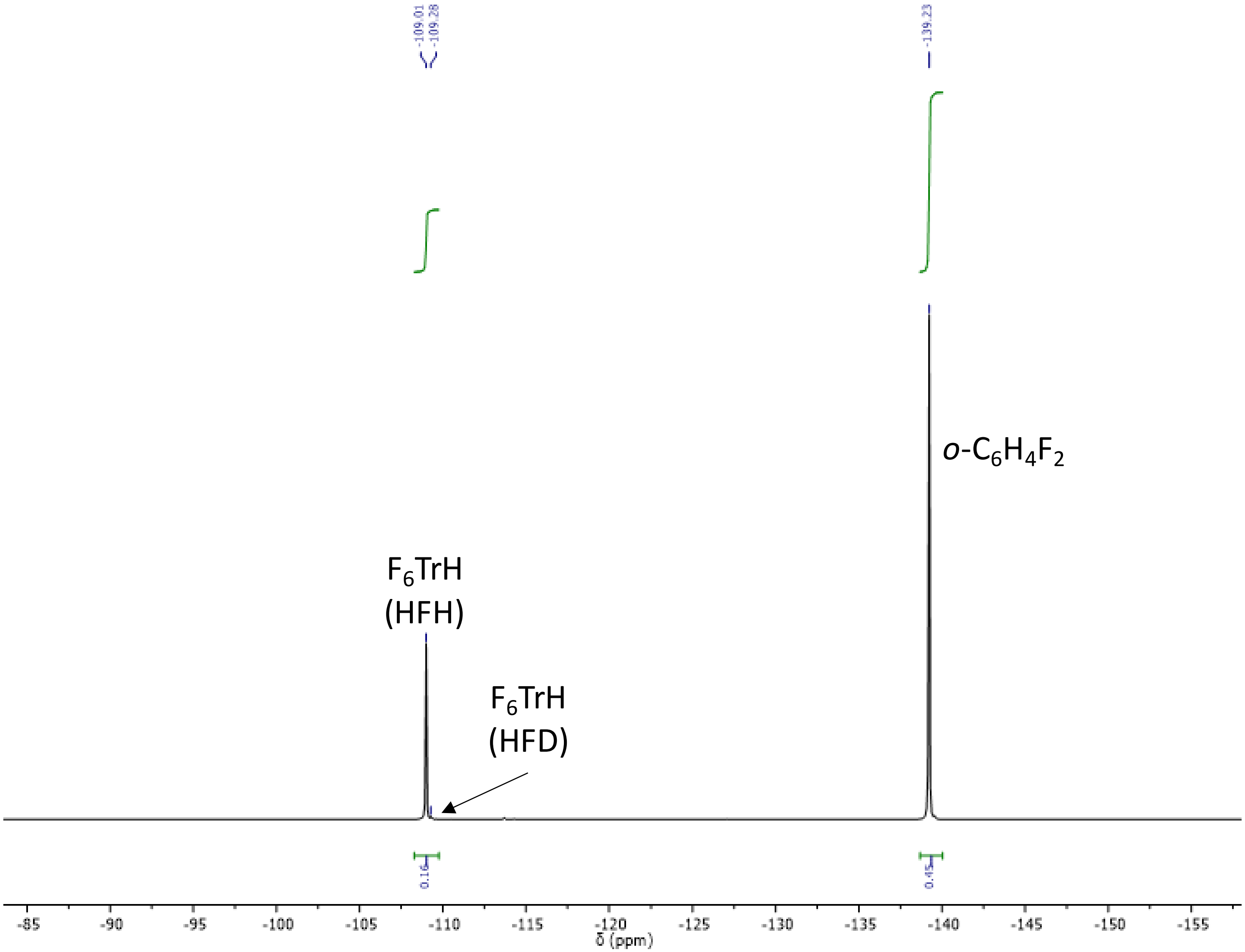

Figure S62. ^{19}F NMR (470 MHz, C_6D_6/*o*-$C_6H_4Cl_2$) spectrum of **F_6Tr[Cl11]** and 2 eq of $HSiEt_3$. < 2%% of H/D exchanged **F_6TrH** can be observed after 10 min (**R4**).

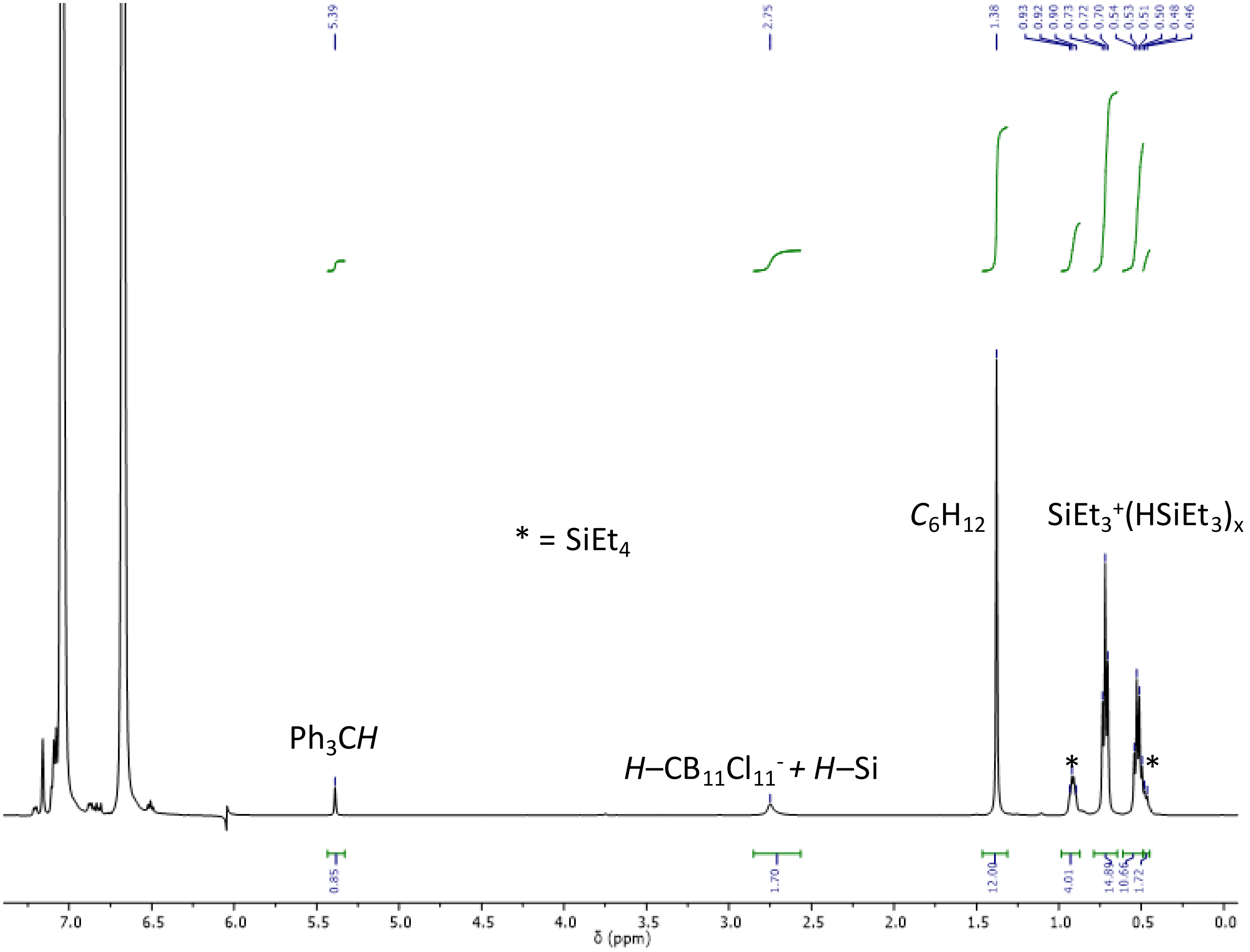

Figure S63. ^{1}H NMR (500 MHz, C_6D_6/*o*-$C_6H_4Cl_2$) spectrum of **Tr[Cl11]** and 2 eq of $HSiEt_3$ (**R5**).

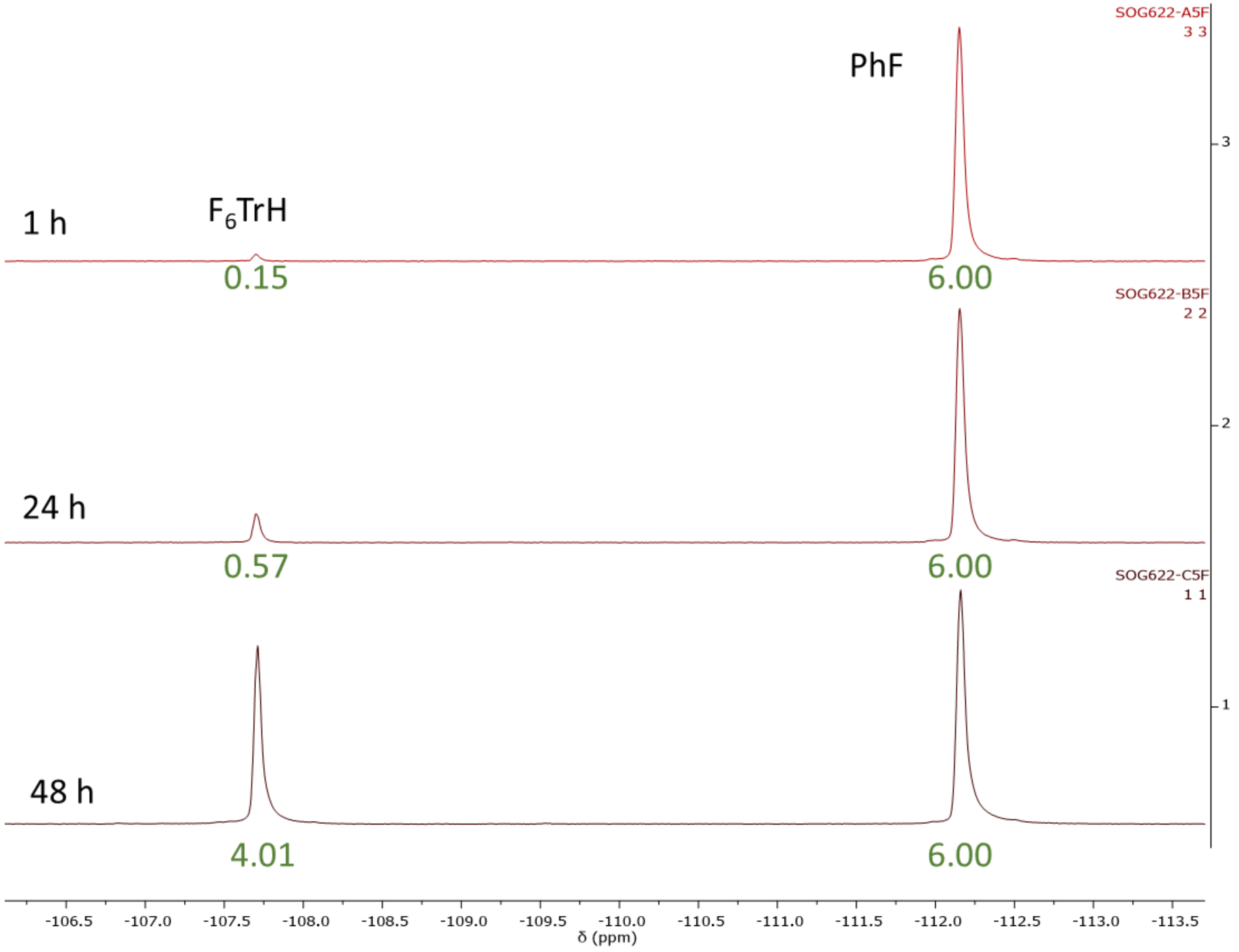

Figure S64. ^{19}F NMR (470 MHz, mesitylene) spectra of **F_6Tr[Cl11]** in mesitylene after 1 h (top), 24 h (middle), and 48 h (bottom). Relative integration ratios are shown in green (**R6**).

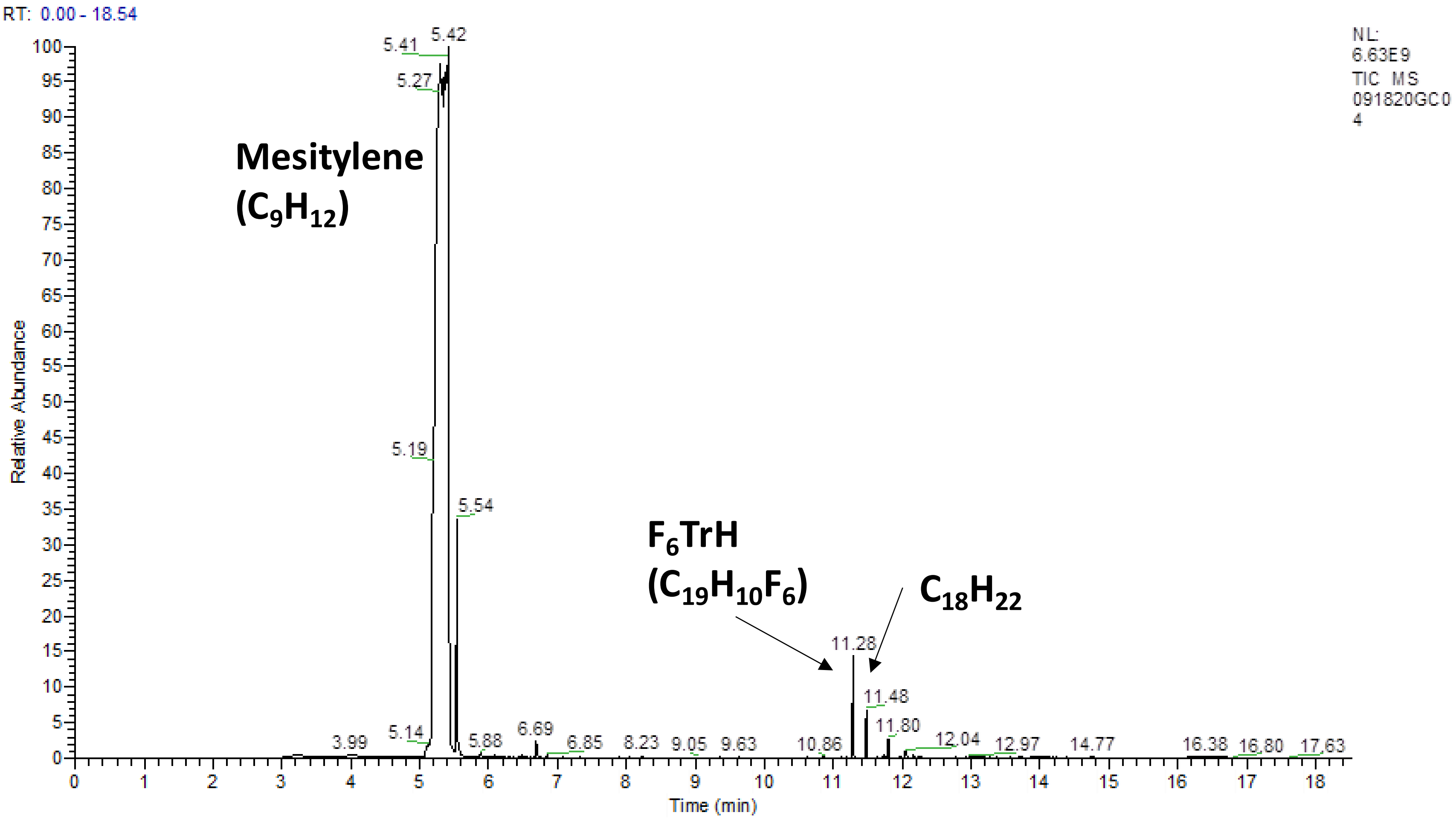

Figure S65. GC trace of **R6**.

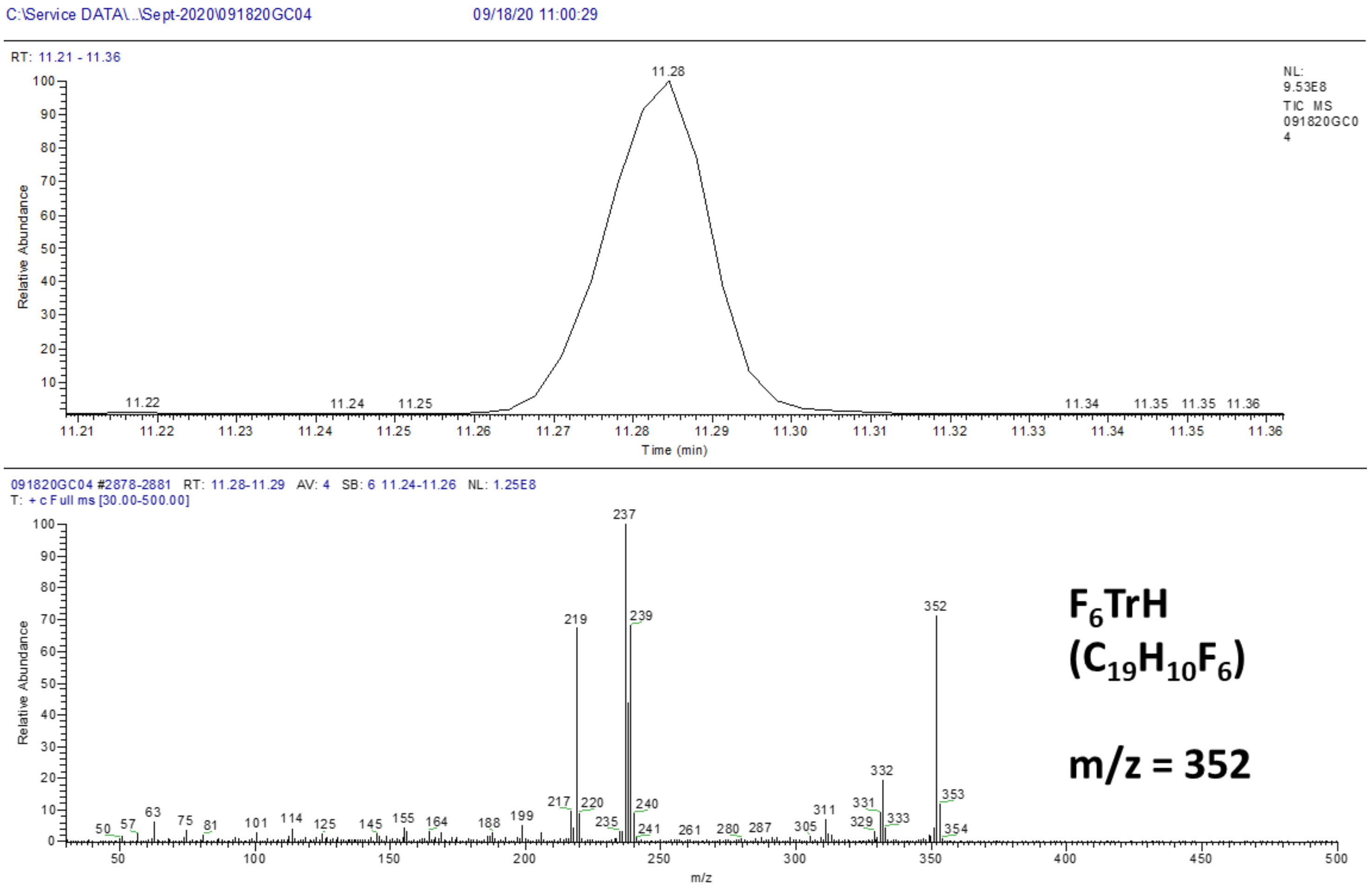

Figure S66. GC/MS spectrum of **F$_6$TrH** from **R6**.

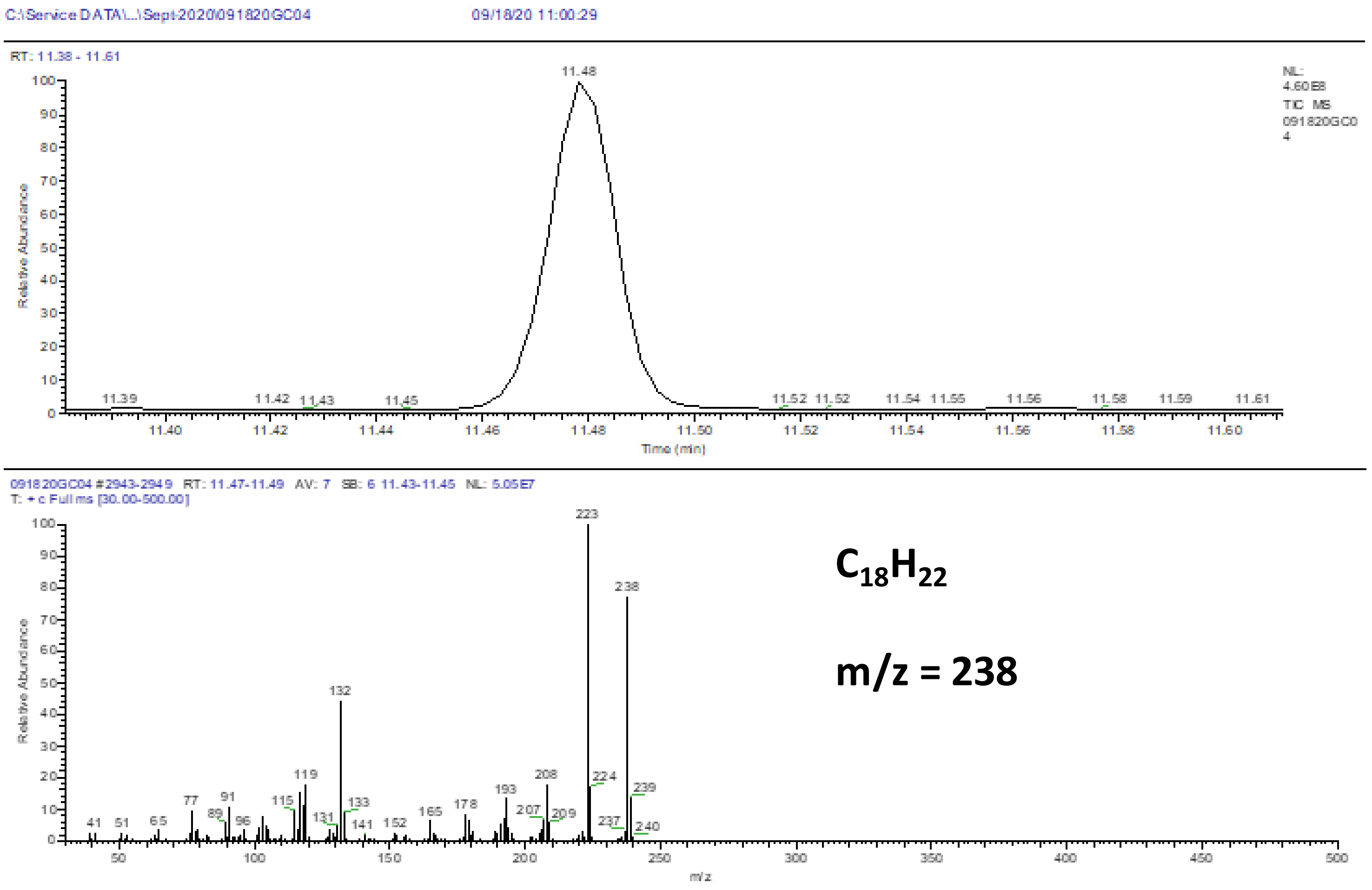

Figure S67. GC/MS spectrum of $C_{18}H_{22}$ from **R6**.

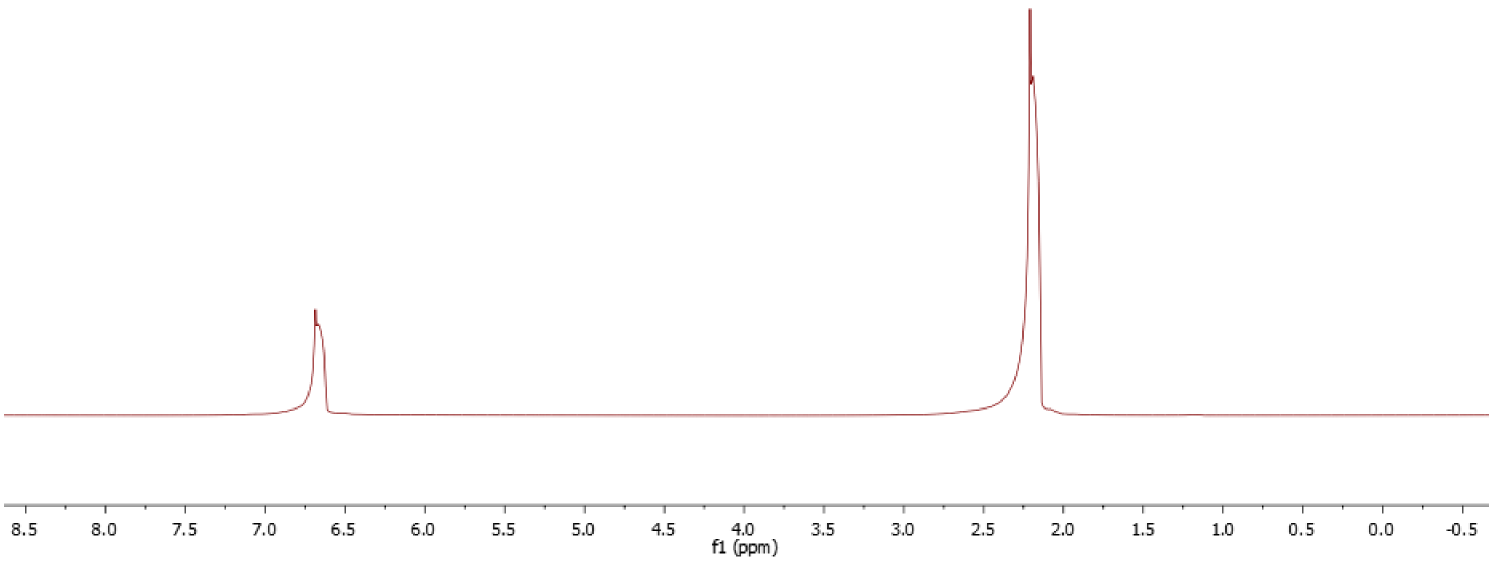

Figure S68. ^{1}H NMR (500 MHz, mesitylene) spectrum of **Tr[Cl11]** in excess mesitylene two weeks at room temperature (**R7**).

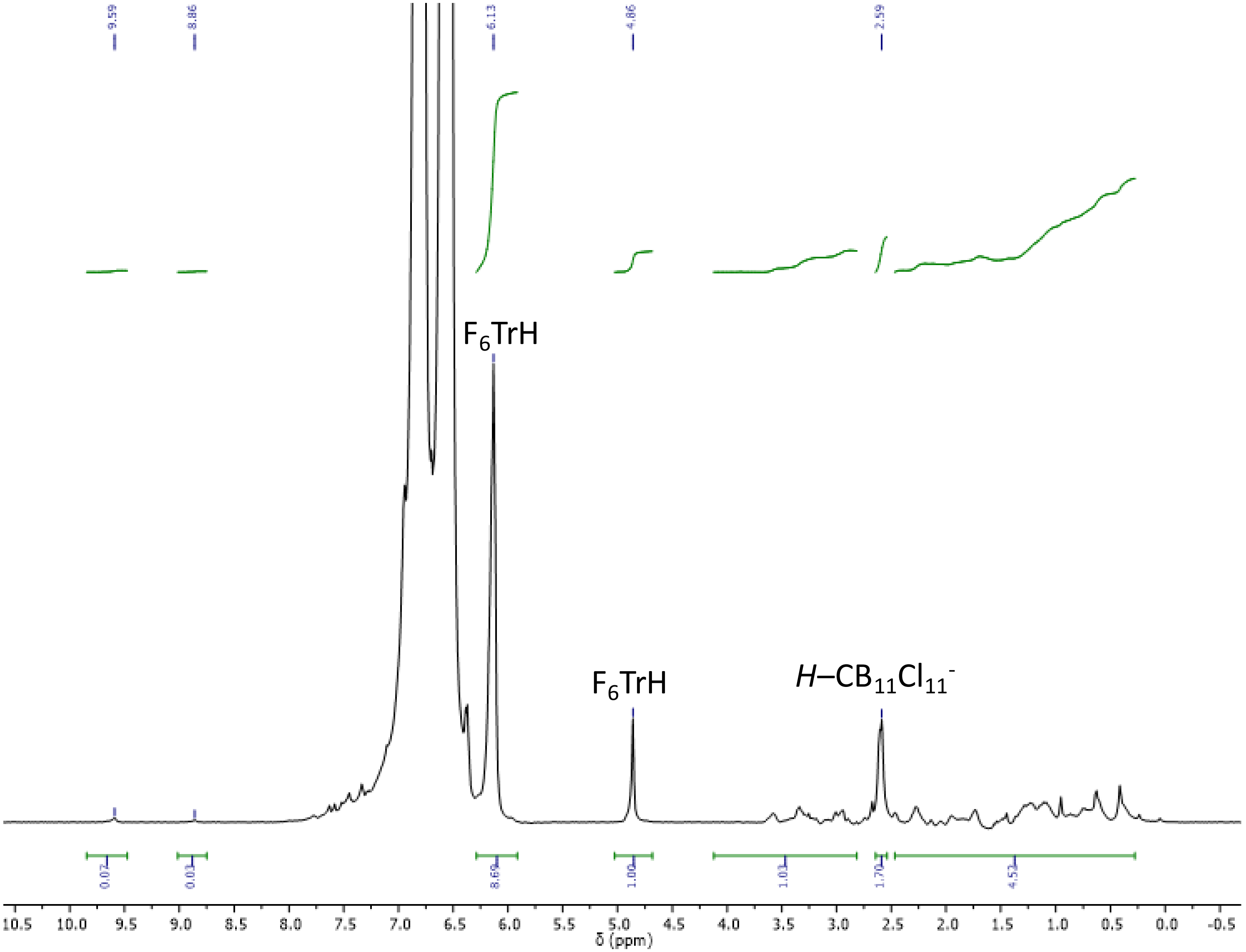

Figure S69. ^{1}H NMR (500 MHz, *o*-$C_6H_4Cl_2$) spectrum taken 4 days after reaction of **F_6Tr[Cl_{11}]** with 1 eq of methylcyclohexane (**R8**).

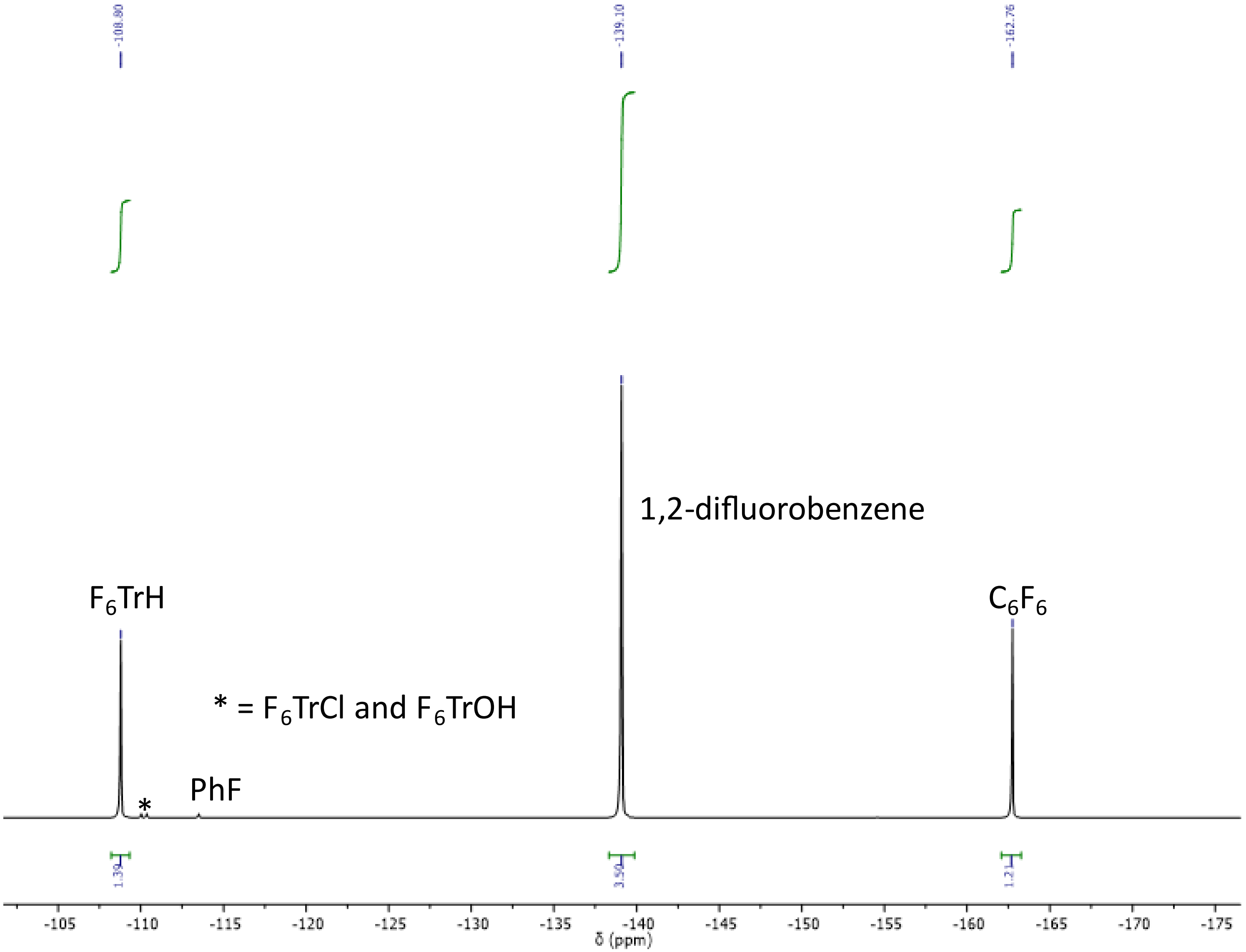

Figure S70. ^{19}F NMR (470 MHz, *o*-$C_6H_4Cl_2$) spectrum taken 4 days after reaction of **F_6Tr[Cl11]** with 1 eq of methylcyclohexane (**R8**).

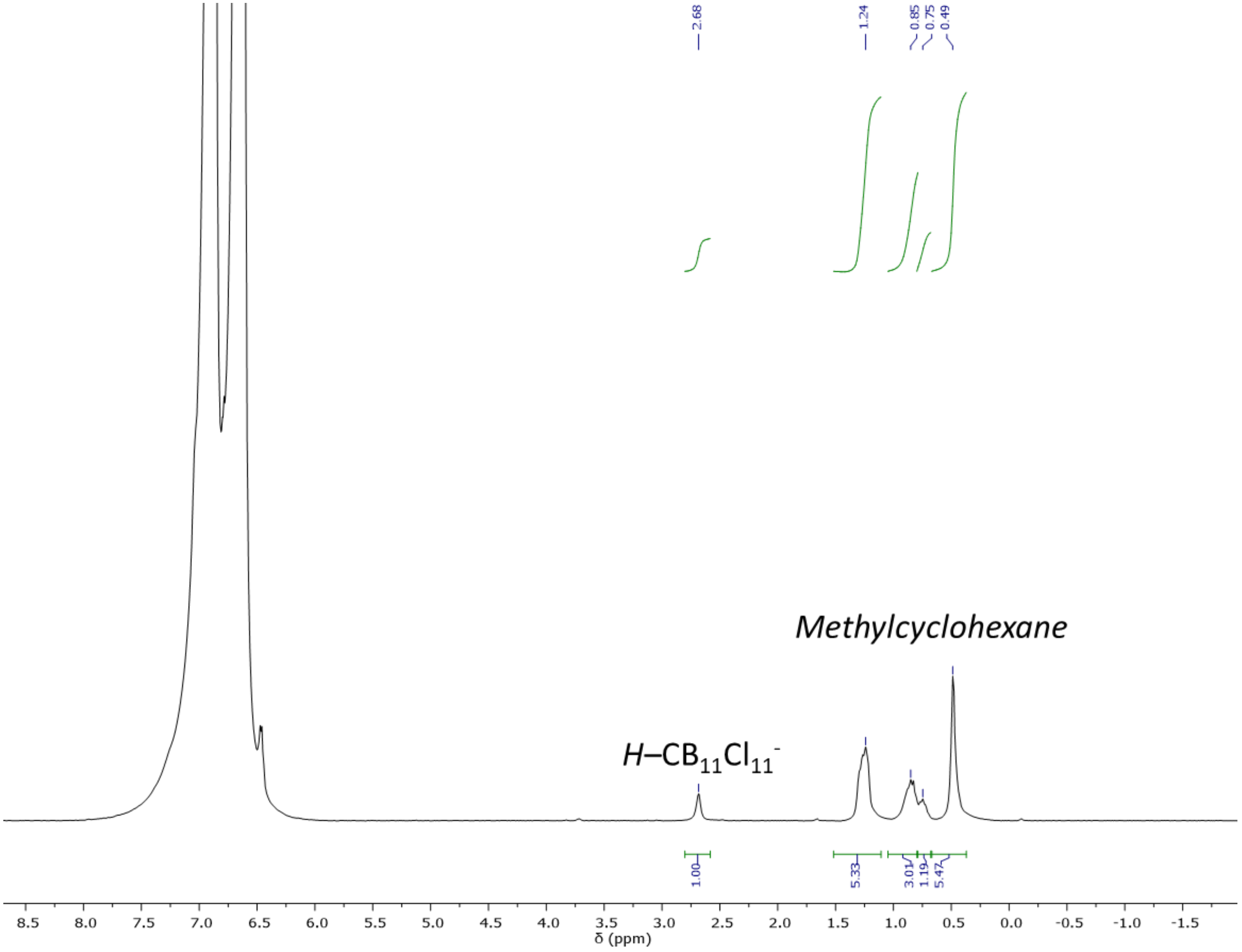

Figure S71. ^{1}H NMR (500 MHz, *o*-$C_6H_4Cl_2$) spectrum taken 4 days after reaction of **Tr[Cl11]** with 1 eq of methylcyclohexane (**R9**).

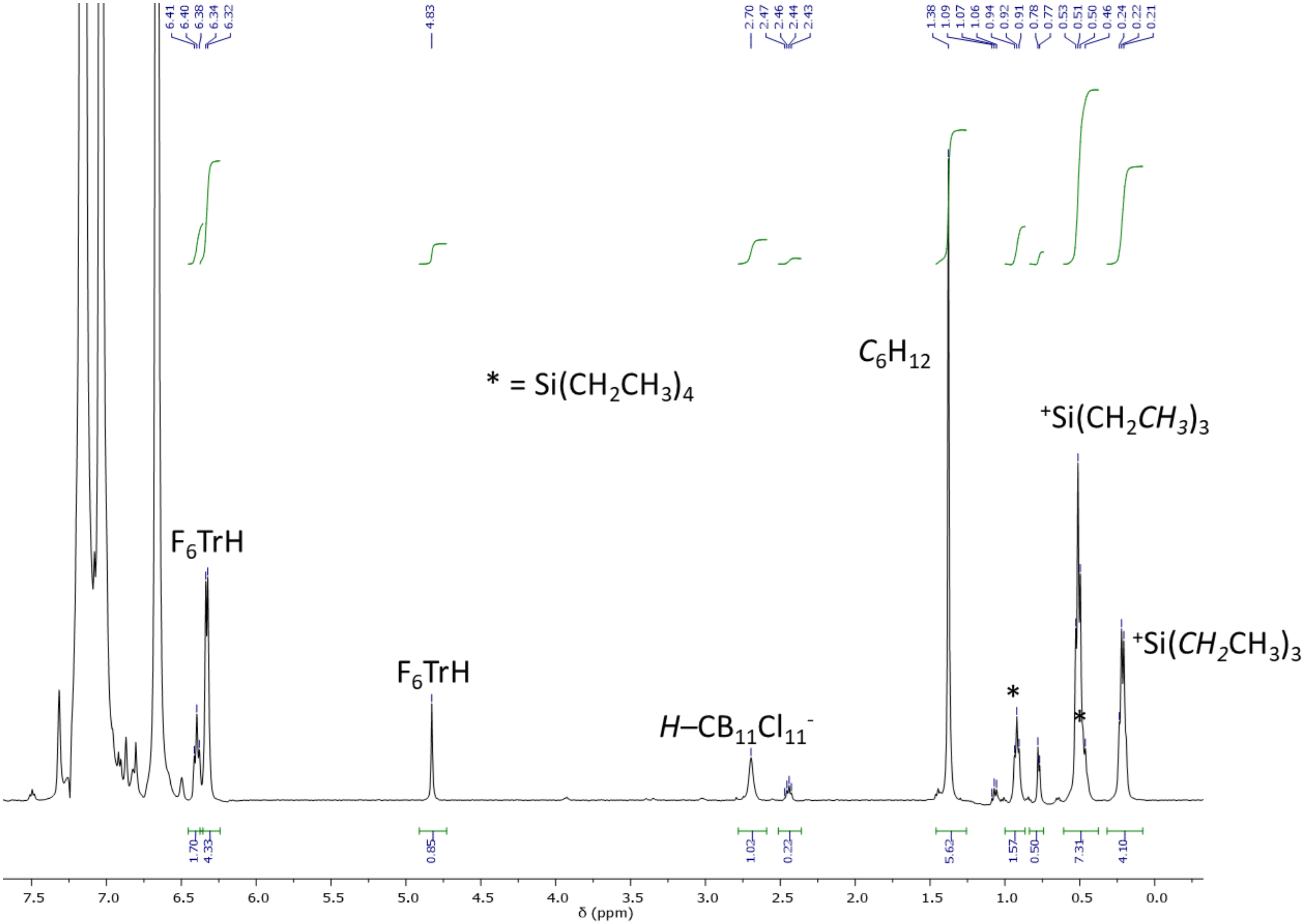

Figure S72. ^{1}H NMR (500 MHz, C_6D_6/*o*-$C_6H_4Cl_2$) spectrum taken 5 days after reaction of **F_6Tr[Cl11]** with 1 eq $SiEt_4$ (**R10**).

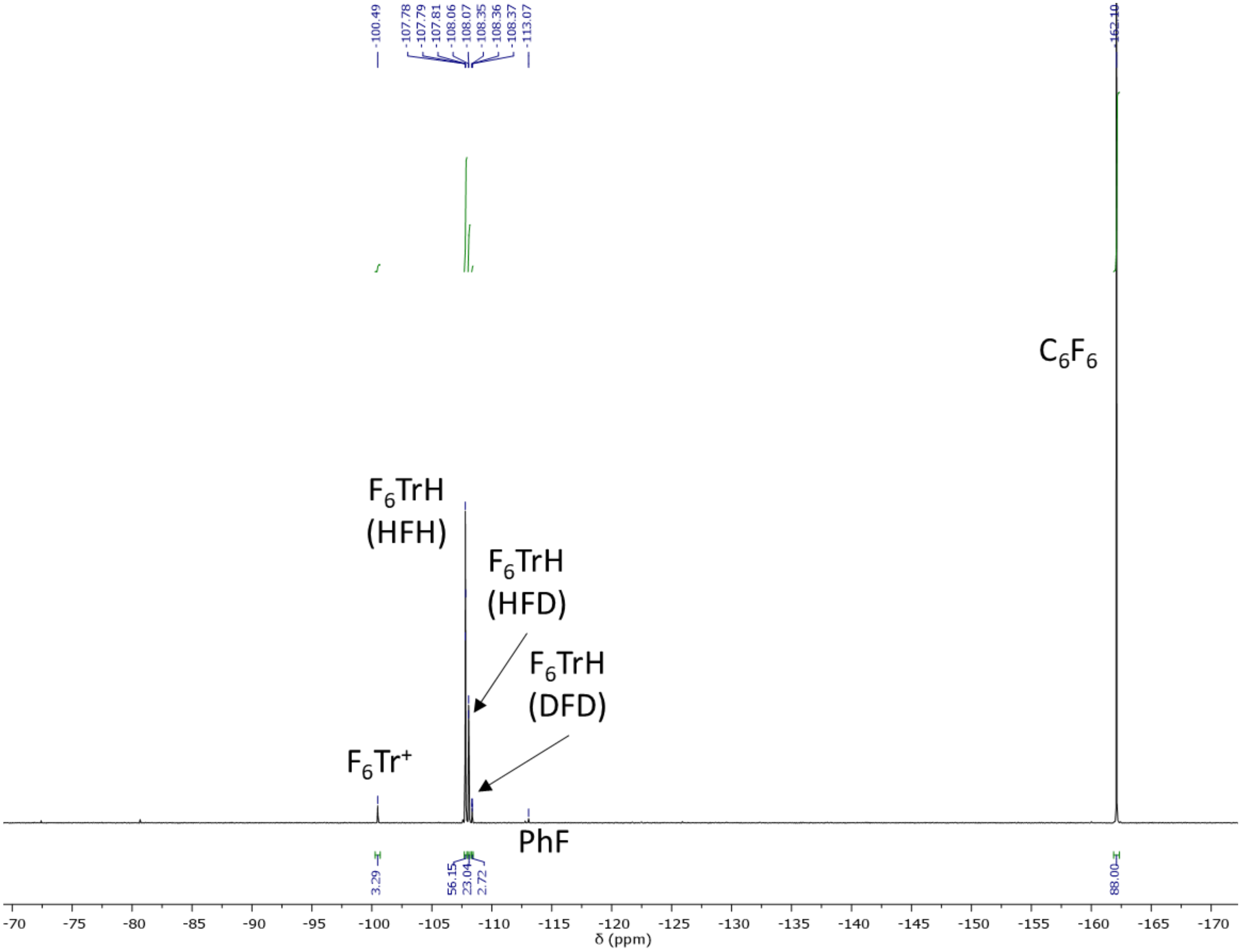

Figure S73. ^{19}F NMR (470 MHz, C_6D_6/*o*-$C_6H_4Cl_2$) spectrum taken 5 days after reaction of **F_6Tr[Cl11]** with 1 eq $SiEt_4$ (**R10**).

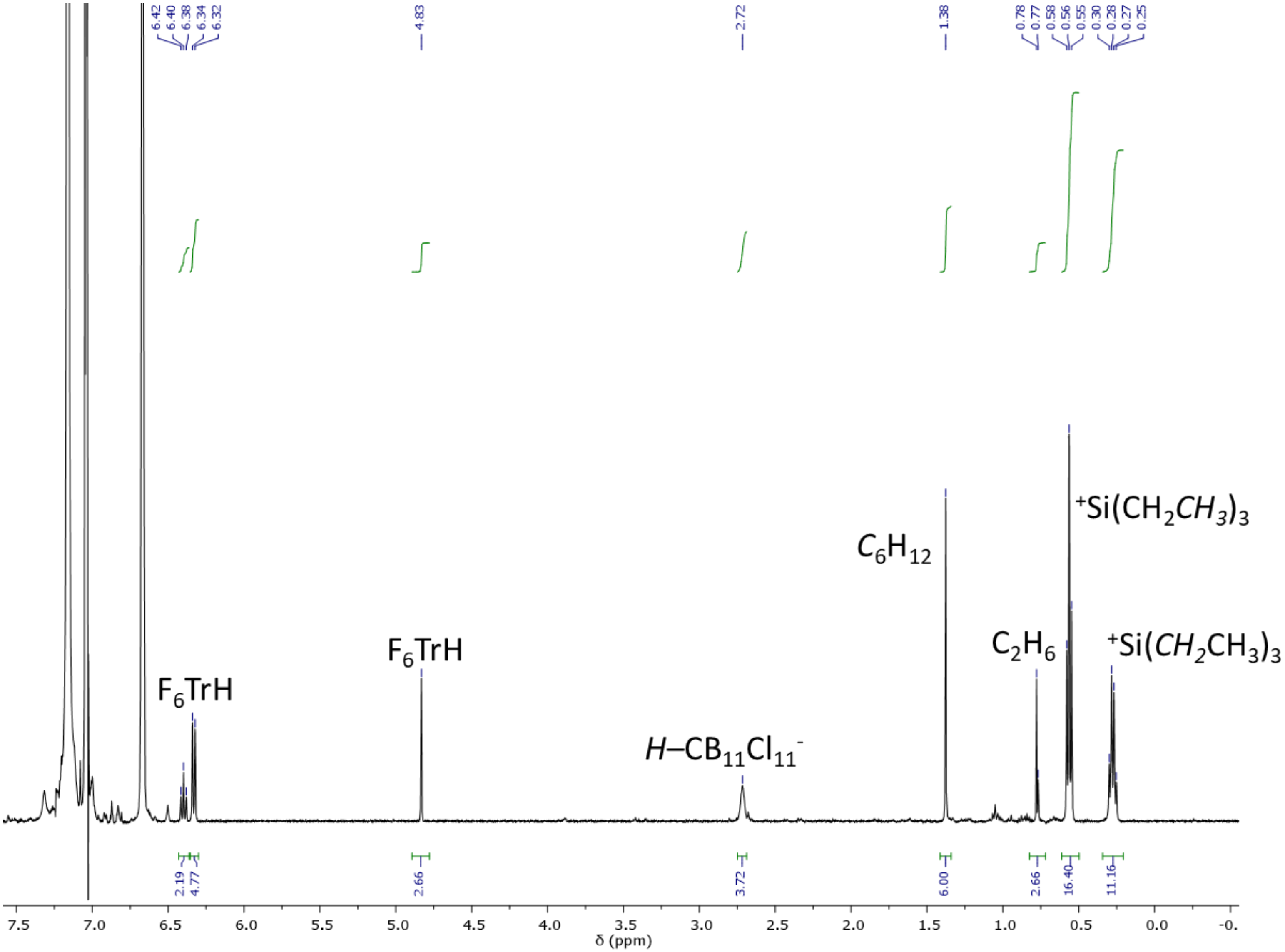

Figure S74. ^{1}H NMR spectrum (500 MHz, C_6D_6/*o*-$C_6H_4Cl_2$) of **F_6Tr[Cl11]** and $SiEt_4$ after 10 days. No $SiEt_4$ is observed, so the reaction is assumed to have gone to completion. A significant amount of ethane is also observed (**R10**).

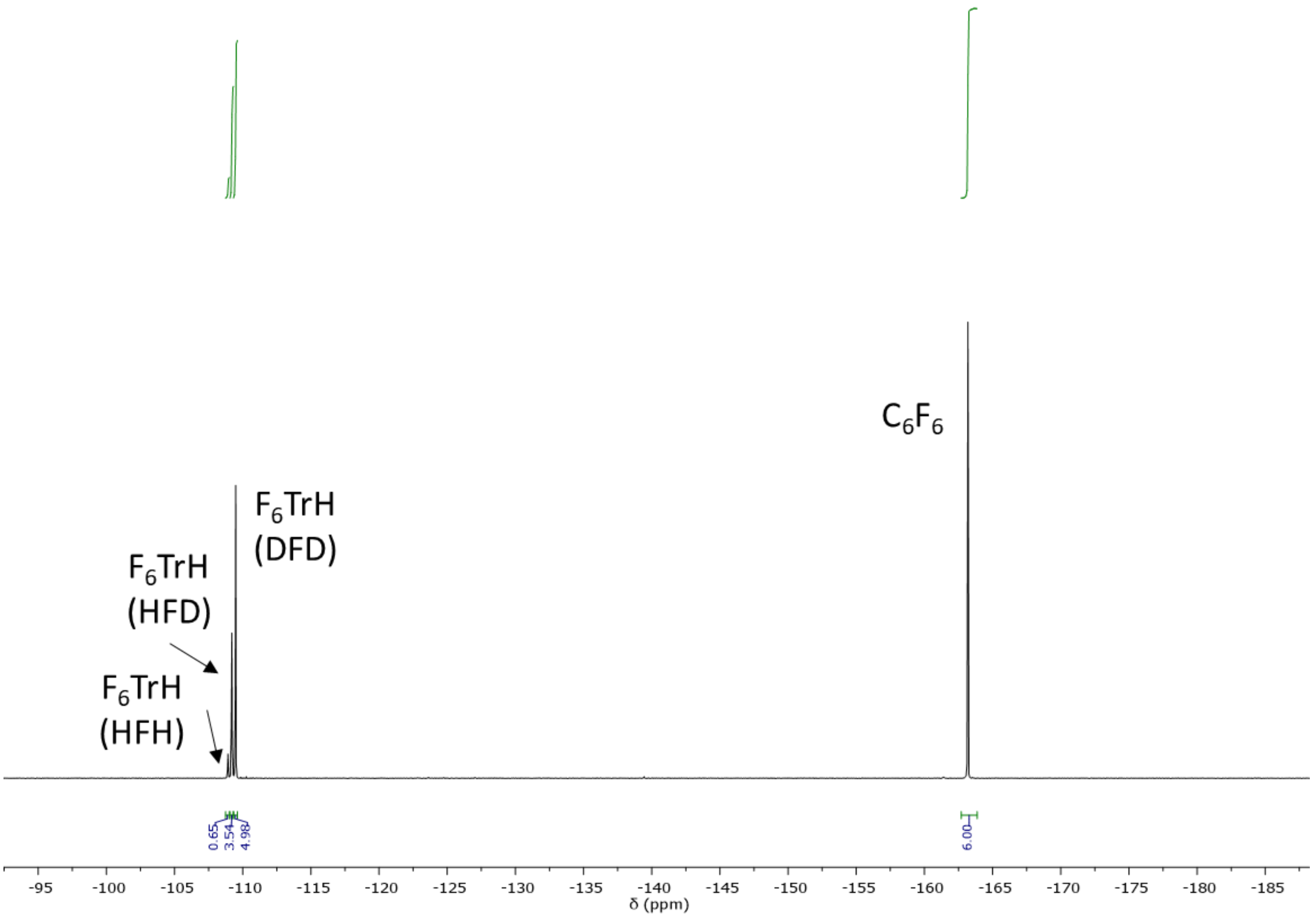

Figure S75. ^{19}F NMR (470 MHz, C_6D_6/*o*-$C_6H_4Cl_2$) spectrum of **F_6Tr[Cl11]** and $SiEt_4$ after 10 days (**R10**).

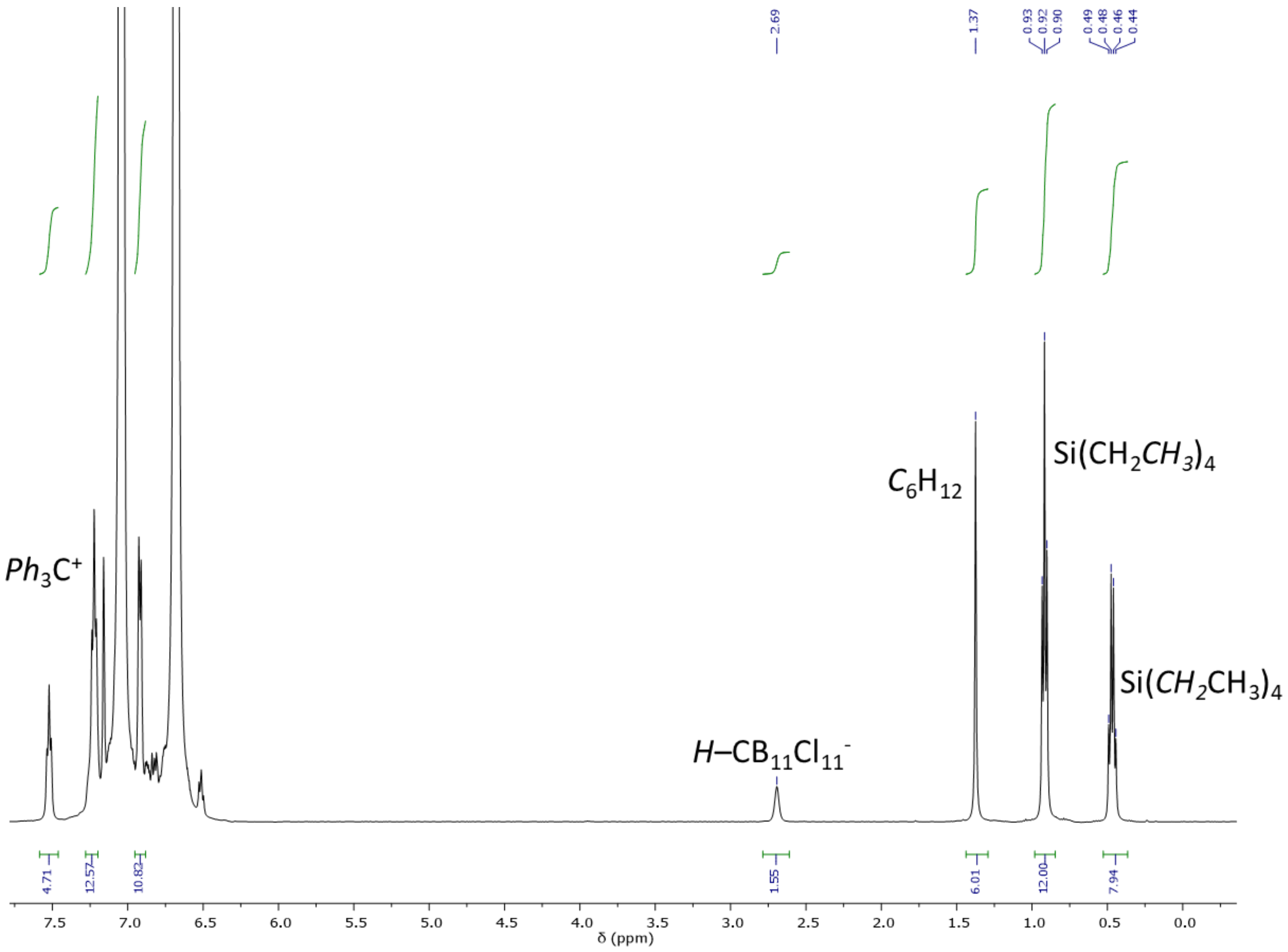

Figure S76. ^{1}H NMR (500 MHz, C_6D_6/*o*-$C_6H_4Cl_2$) spectrum taken 5 days after reaction of **Tr[Cl11]** with 1 eq $SiEt_4$ (**R11**).

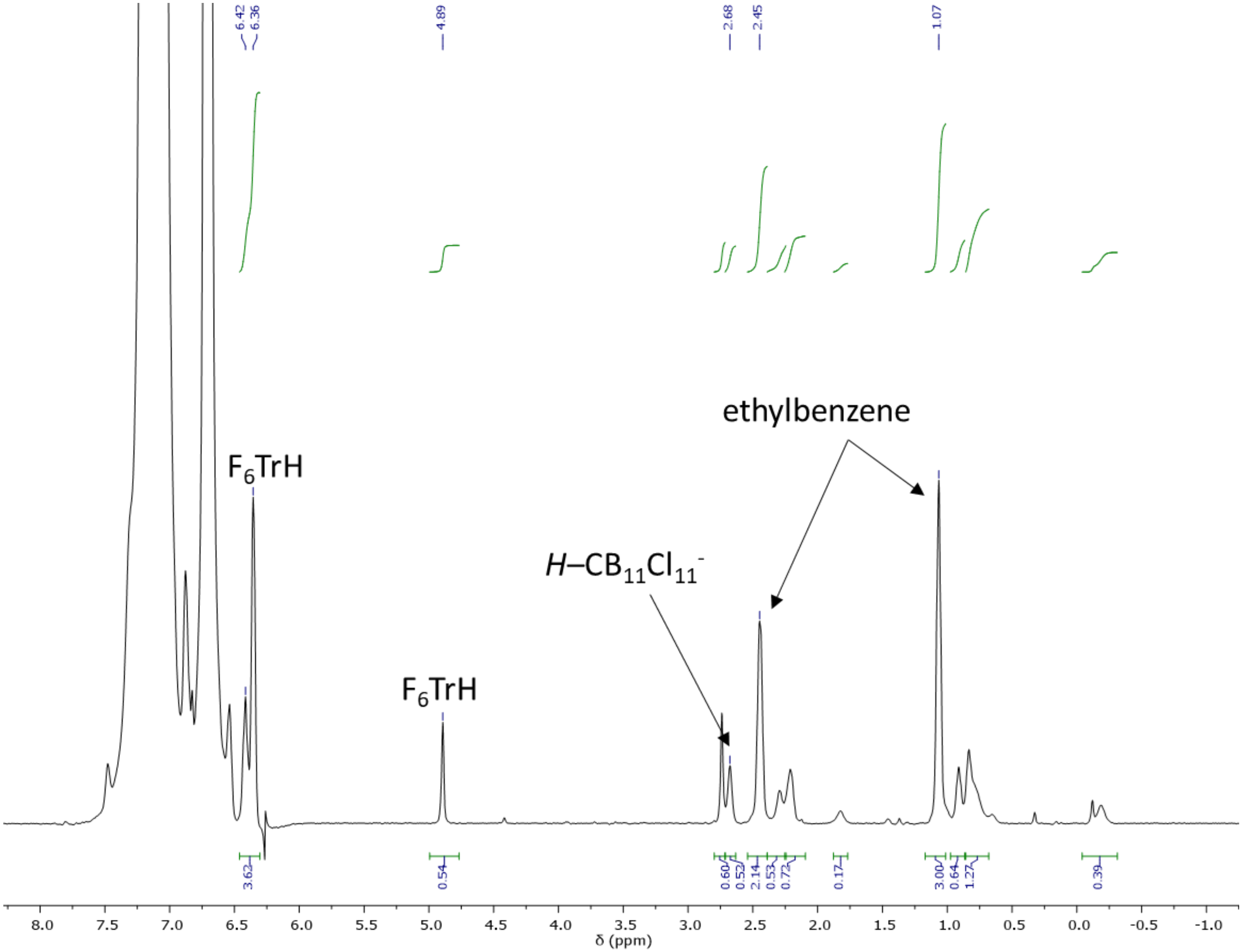

Figure S77. ^{1}H NMR (500 MHz, C_6H_6/*o*-$C_6H_4Cl_2$) spectrum taken 18 h after addition of ethylene to **F_6Tr[Cl11]** (**R12**).

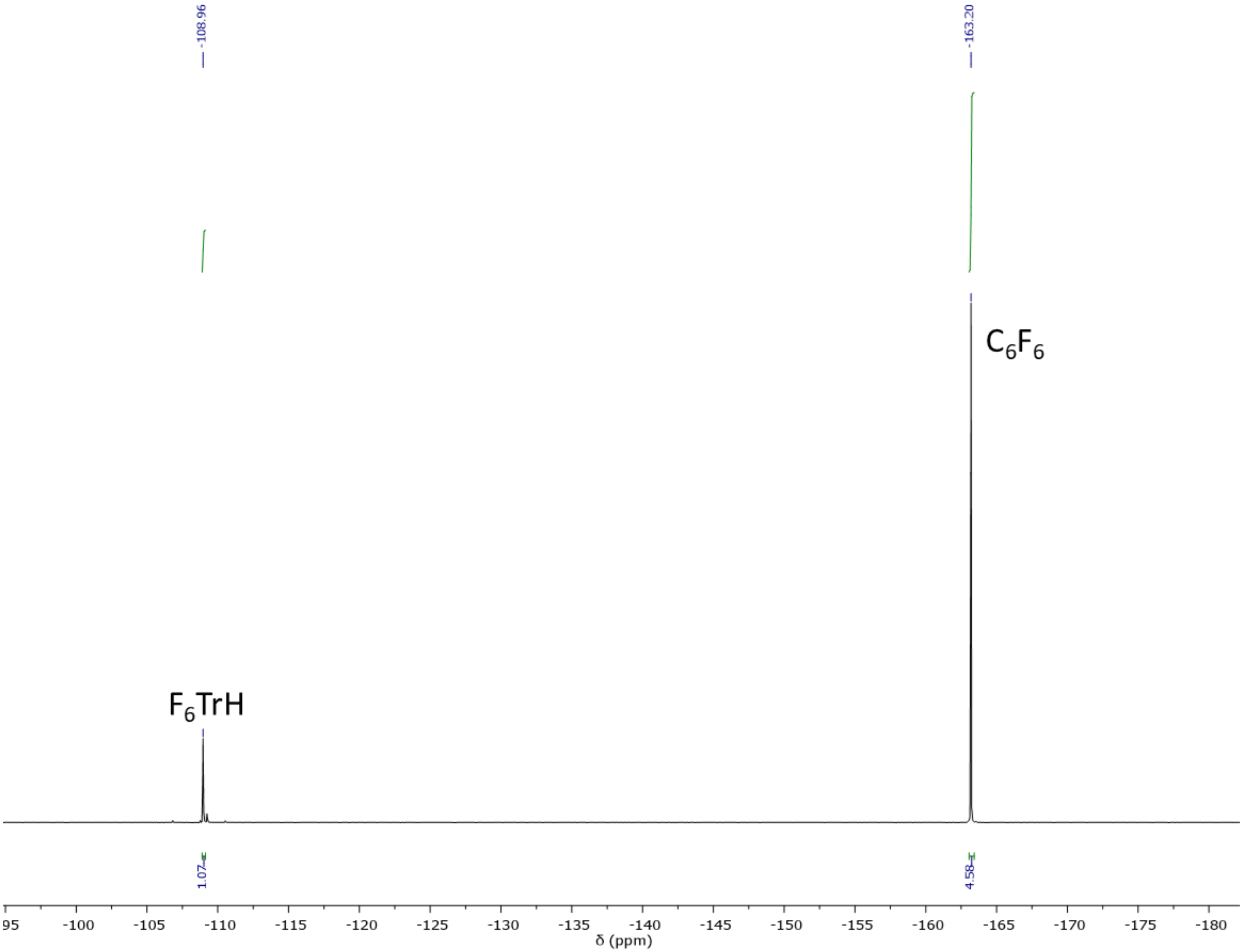

Figure S78. ^{19}F NMR (500 MHz, C_6H_6/*o*-$C_6H_4Cl_2$) spectrum taken 18 h after addition of ethylene to **F$_6$Tr[Cl11]** (**R12**).

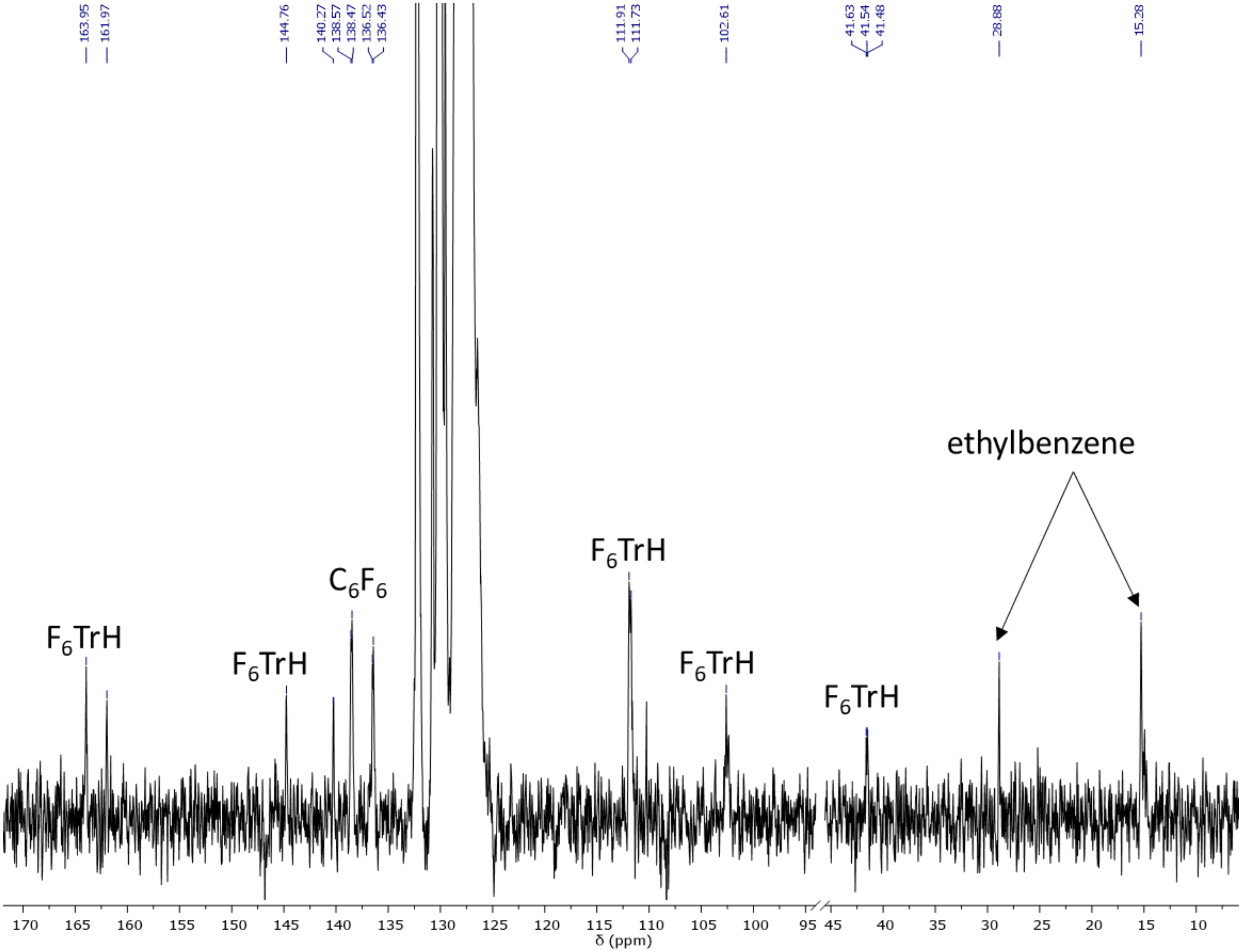

Figure S79. $^{13}C\{^1H\}$ NMR (126 MHz, C_6H_6/*o*-$C_6H_4Cl_2$) spectrum taken 18 h after addition of ethylene to **F_6Tr[Cl11]** (**R12**).

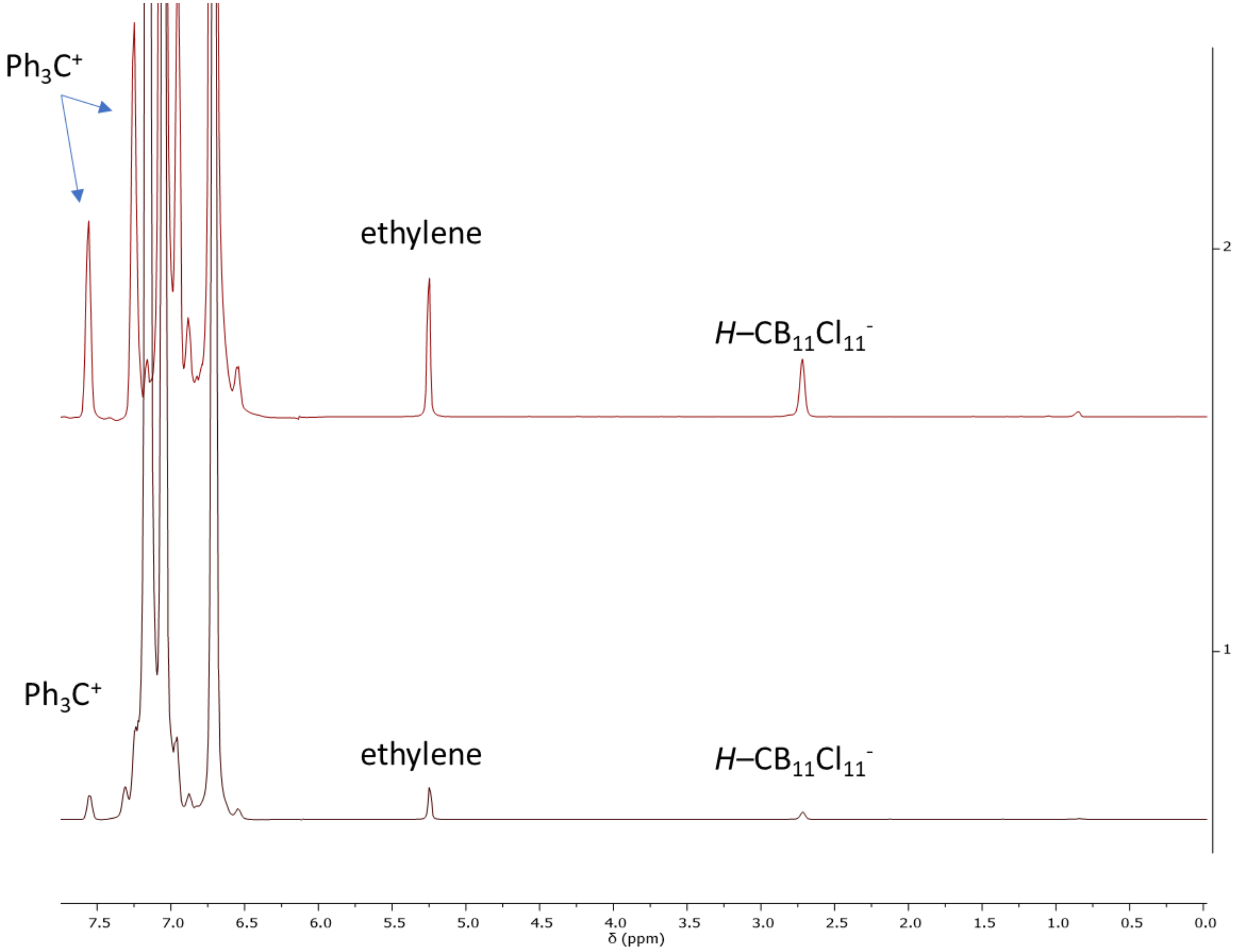

Figure S80. ^{1}H NMR (500 MHz) spectra taken 7 days after addition of ethylene to **Tr[Cl11]** in C_6D_6/*o*-$C_6H_4Cl_2$ (top) and C_6H_6/*o*-$C_6H_4Cl_2$ (bottom, **R13**).

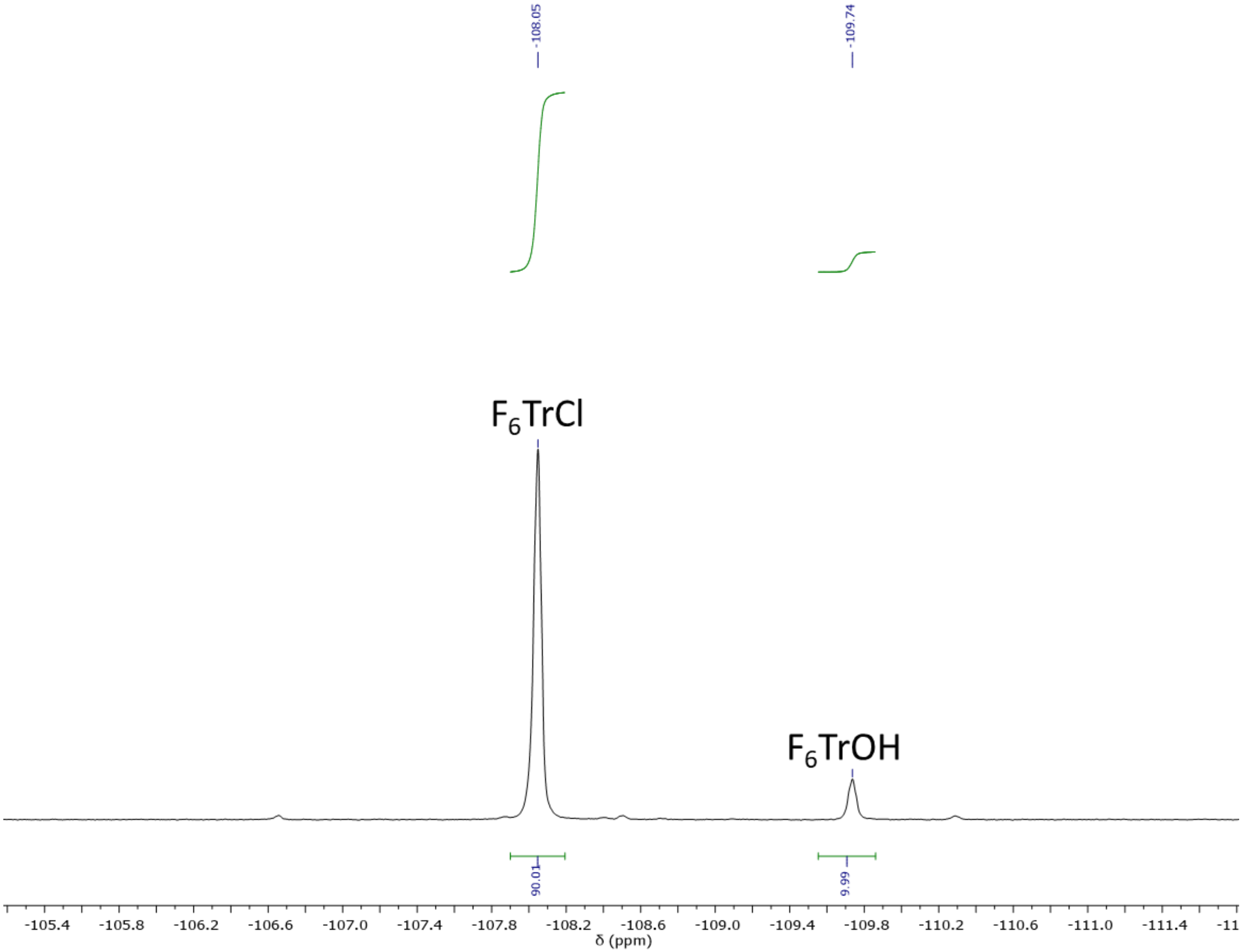

Figure S81. ^{19}F NMR (470 MHz, *o*-$C_6H_4Cl_2$) spectrum of **F_6Tr[Cl11]** and 4.6 eq of Et_4NCl (**R14**).

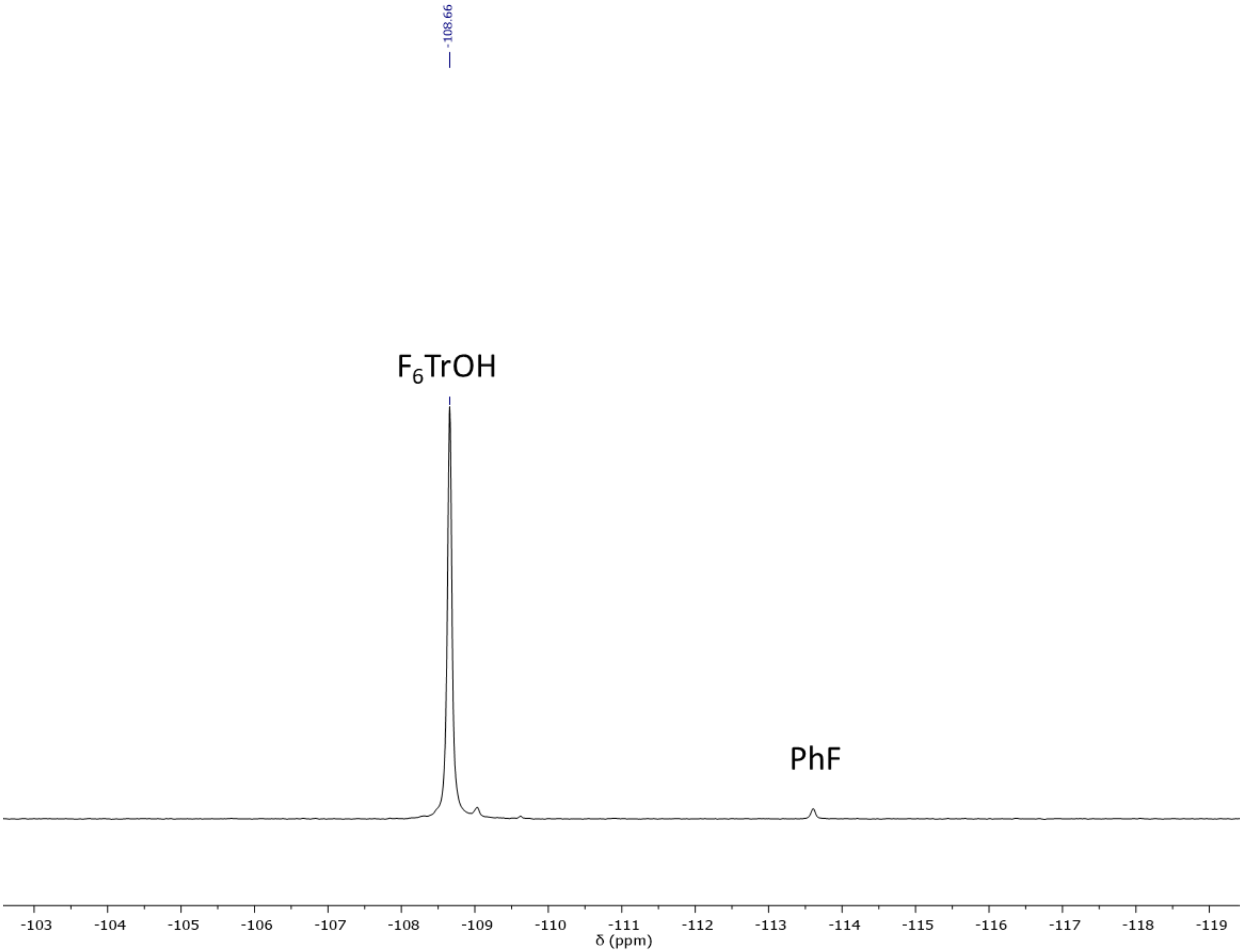

Figure S82. ^{19}F NMR (470 MHz, C_6D_6) spectra of **F_6Tr[Cl11]** in the presence of water. Residual PhF is observed (**R15**).

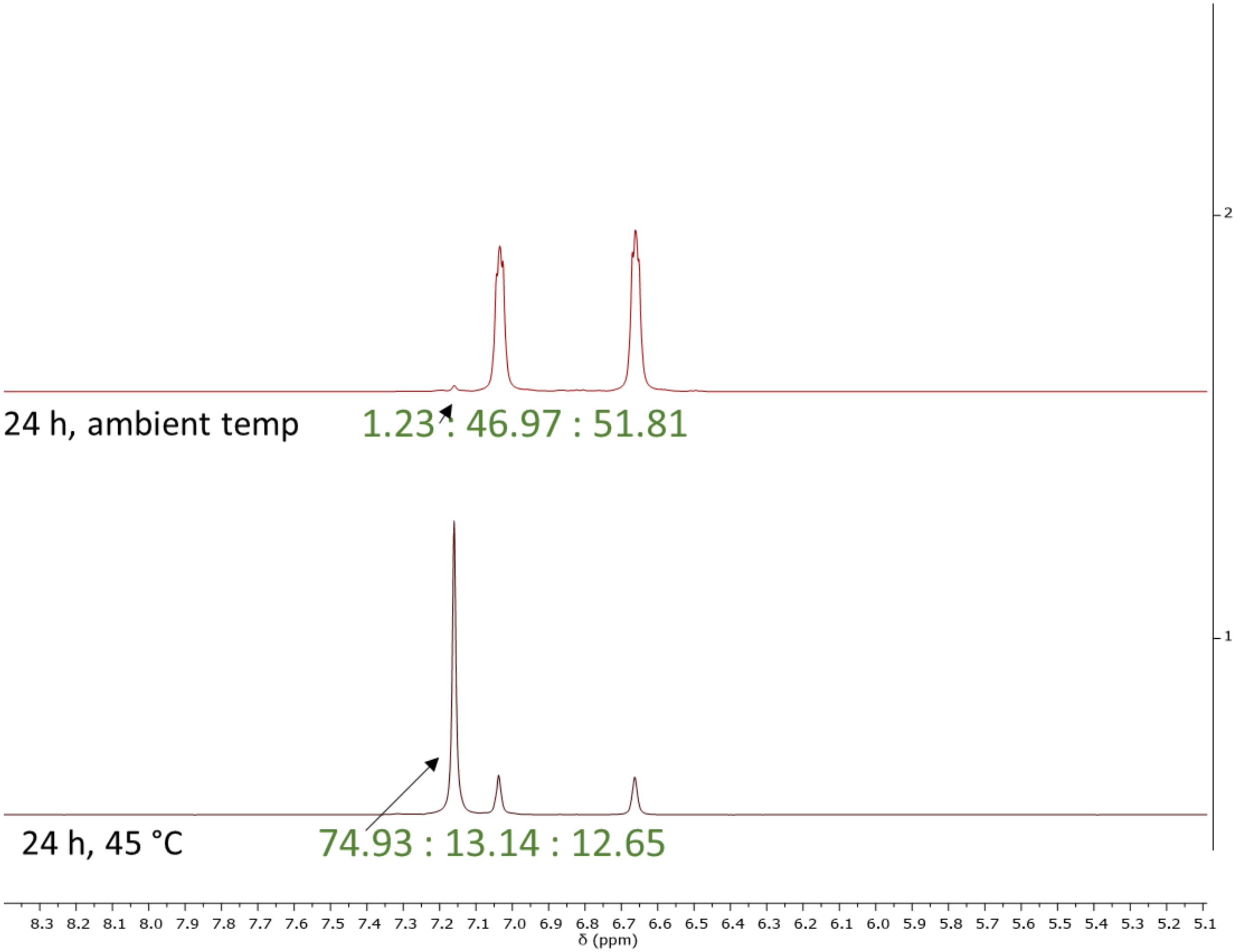

Figure S83. ^{1}H NMR (500 MHz, C_6D_6/*o*-$C_6H_4Cl_2$) spectra of **F_6Tr[Cl11]** (0.4 mol%) in the solvent mixture taken after 24 h at ambient temp (top) and 24 h at 45 °C (bottom, **R16**).

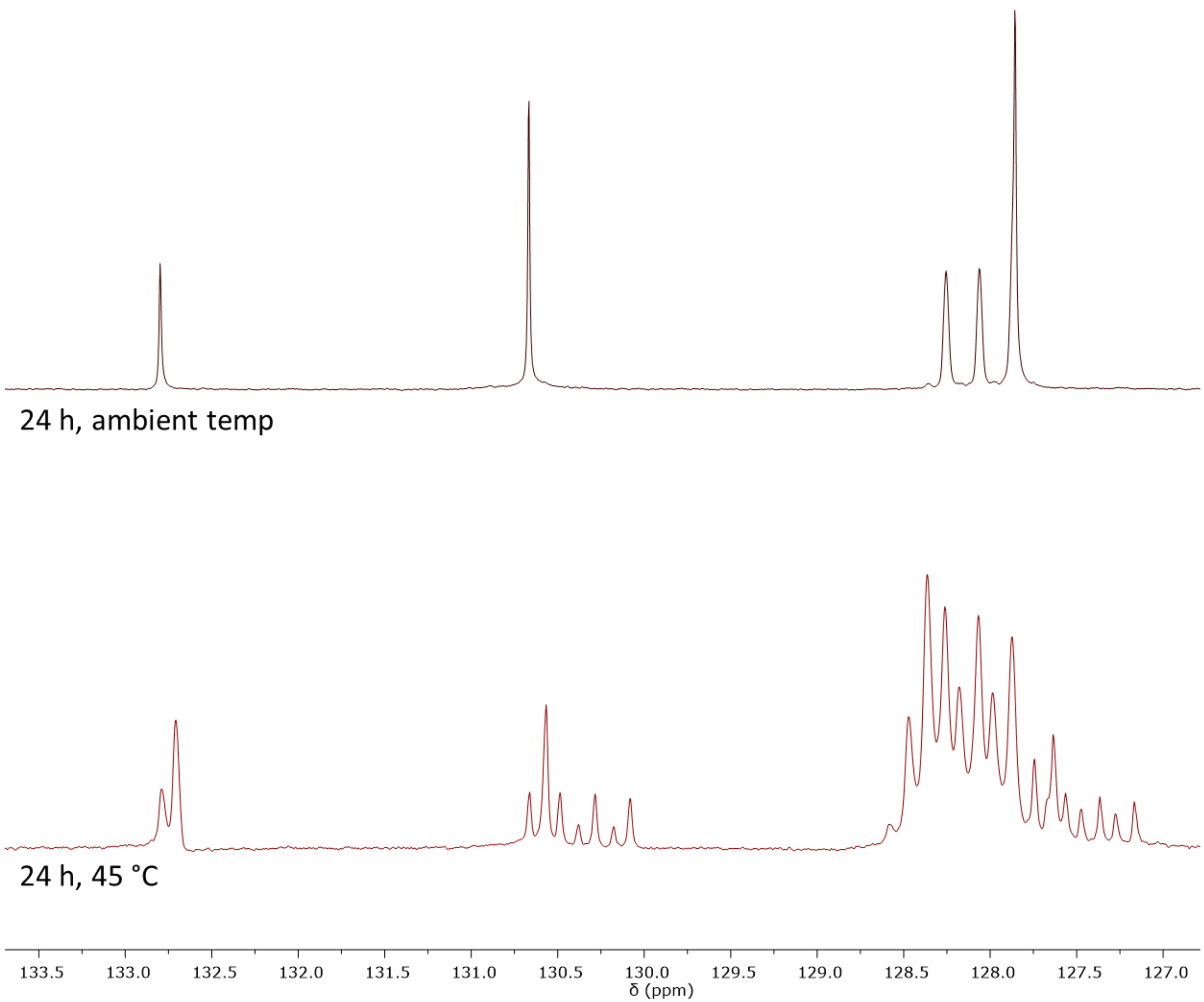

Figure S84. $^{13}C\{^1H\}$ NMR (126 MHz) spectrum of C_6D_6 and *o*-$C_6H_4Cl_2$ in the presence of **F_6Tr[Cl11]** after 24 hours at ambient temperature (top) and 24 hours at 45 °C (bottom, **R16**).

X. References

[1] W. Gu, B. J. McCulloch, J. H. Reibenspies, and O. V. Ozerov, *Chem. Commun.*, 2010, **46**, 282-2822. https://doi.org/10.1039/C001555E

[2] A. Schulz, J. Thomas and A. Villinger, *Chem. Commun.*, 2010, **46**, 3696-3698. https://doi.org/10.1039/C0CC00013B.

[3] L.P. Press, D. J. McCulloch, W. Gu, C. -H. Chen, B. M. Foxman and O. V. Ozerov, *Chem. Commun.*, 2015, **51**, 14034-14037. https://doi.org/10.1039/C5CC05047B.

[4] J. R. Bour, J. C. Green, V. J. Winton and J. B. Johnson, *J. Org. Chem.*, 2013, **78**, 1665-1669. https://doi.org/10.1021/jo302592g.

[5] M. Horn, C. Metz and H. Mayr, *Eur. J. Org. Chem.*, 2011, 6476–6485. https://doi.org/10.1002/ejoc.201100912.

[6] APEX3, Program for Data Collection on Area Detectors, BRUKER AXS Inc., 5465 East Cheryl Parkway, Madison, WI 53711-5373 USA.

[7] G. M. Sheldrick, SADABS, Program for Absorption Correction of Area Detector Frames, BRUKER AXS Inc., 5465 East Cheryl Parkway, Madison, WI 53711-5373 USA.

[8] (a) G. M. Sheldrick, *Acta Cryst.*, 2008, **A64**, 112-122. (b) G. M. Sheldrick, *Acta Cryst.* 2015**,** **A71**, 3-8. (c) G. M. Sheldrick, *Acta Cryst.*, 2015, **C71**, 3-8. (d) XT, XS, BRUKER AXS Inc., 5465 East Cheryl Parkway, Madison, WI 53711-5373 USA.

[9] (a) A. L. Spek, PLATON - A Multipurpose Crystallographic Tool. *J. Appl. Cryst.* 2003, **36**, 7-13. (b) Spek, A. L. Utrecht University, Utrecht, The Netherlands 2008.

[10] O. V. Dolomanov, L. J. Bourhis, R. J. Gildea, J. A. K. Howard and J. H. Puschmann, OLEX2: A Complete Structure Solution, Refinement and Analysis Program. *J. Appl. Cryst.*, 2009, **42**, 339-341.

[11] Persistence of Vision Raytracer, version 3.6; Computer software; Persistence of Vision Pty. Ltd.: Williamstown, Victoria, Australia, 2004. Retrieved from http://www.povray.org/download/

[12] L. Farugia, ORTEP-3 for Windows – a version of ORTEP-III with a Graphical User Interface (GUI). *J. Appl. Cryst.*, 1997, **30**, 565.

[13] M. J. Frisch, G. W. Trucks, H. B. Schlegel, G. E. Scuseria, M. A. Robb, J. R. Cheeseman, G. Scalmani,V. Barone, B. Mennucci, G. A. Petersson, H. Nakatsuji, M. Caricato, X. Li, H. P. Hratchian, A. F. Izmaylov, J. Bloino, G. Zheng, J. L. Sonnenberg, M. Hada, M. Ehara, K. Toyota, R. Fukuda, J. Hasegawa, M. Ishida, T. Nakajima, Y. Honda, O. Kitao, H. Nakai, T. Vreven, J. A. Montgomery Jr, J. E. Peralta, F. Ogliaro, M. Bearpark, J. J. Heyd, E. Brothers, K. N. Kudin, V. N. Staroverov, R. Kobayashi, J. Normand, K. Raghavachari, A. Rendell, J. C. Burant, S. S. Iyengar, J. Tomasi, M. Cossi, N. Rega, J. M. Millam, M. Klene, J. E. Knox, J. B. Cross, V. Bakken, C. Adamo, J. Jaramillo, R. Gomperts, R. E. Stratmann, O. Yazyev, A. J. Austin, R. Cammi, C. Pomelli, J. W. Ochterski, R. L. Martin, K. Morokuma, V. G. Zakrzewski, G. A. Voth, P. Salvador, J. J. Dannenberg, S. Dapprich, A. D. Daniels, O. Farkas, J. B. Foresman, J. V. Ortiz, J. Cioslowski and D. J. Fox, Gaussian 09, revision D.01, Gaussian, Inc., Wallingford, CT, 2009.

[14] Y. Zhao, N. E. Schultz and D. G. Truhlar, *J. Chem. Theory Comput.*, 2006, **2**, 364-382. https://doi.org/10.1021/ct0502763.

[15] D. G. Gusev and O. V. Ozerov, *Chem. Eur. J.*, 2011, **17**, 634-640. https://doi.org/10.1002/chem.201000696.

[16] V. Salamanca and A. C. Albeniz, *Eur. J. Org. Chem.*, 2020, **22**, 3206-3212. https://doi.org/10.1002/ejoc.202000284.

[17] S. J. Connelly, W. Kaminsky and D. M. Heinekey, D. M., *Organometallics*, 2013, **32**, 7478-7481. https://doi.org/10.1021/om400970j.
